# Novel Dibenzoazepine-Substituted Triazole Hybrids as Cholinesterase and Carbonic Anhydrase Inhibitors and Anticancer Agents: Synthesis, Characterization, Biological Evaluation, and *In Silico* Studies

**DOI:** 10.1021/acsomega.4c05804

**Published:** 2024-11-16

**Authors:** Musa Erdoğan, Alper Onder, Yeliz Demir, Ferah Comert Onder

**Affiliations:** † Department of Food Engineering, Faculty of Engineering and Architecture, Kafkas University, 36100 Kars, Türkiye; ‡ Natural Products and Drug Research Laboratory, Department of Chemistry, Faculty of Science, Çanakkale Onsekiz Mart University, 17020 Çanakkale, Türkiye; § Nihat Delibalta Gole Vocational High School, Department of Pharmacy Services, Ardahan University, 75700 Ardahan, Türkiye; ∥ Department of Medical Biology, Faculty of Medicine, Çanakkale Onsekiz Mart University, 17020 Çanakkale, Türkiye

## Abstract

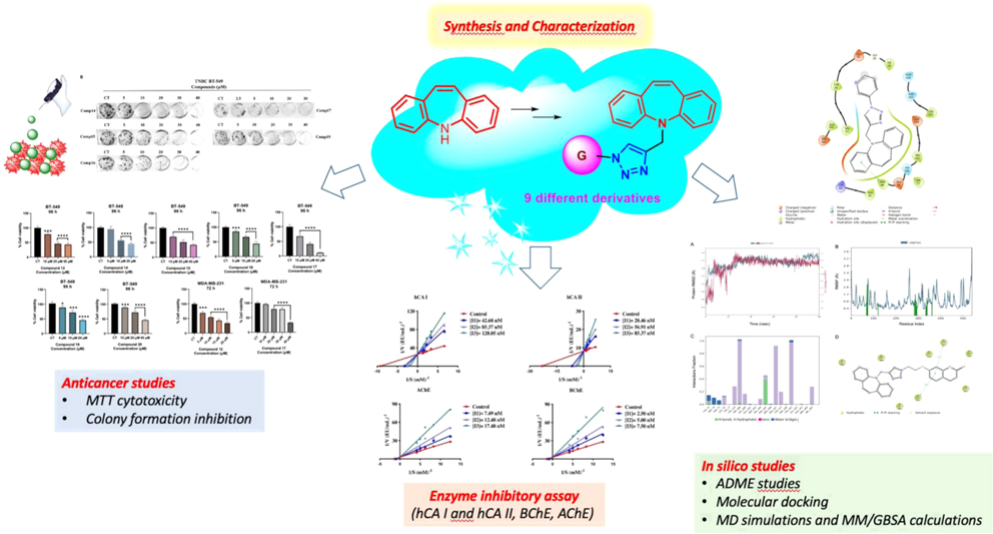

The new dibenzoazepine-substituted triazole hybrids (**12**–**20**) were designed by molecular hybridization approach and synthesized utilizing the Cu(I)-catalyzed click reaction. The hybrid structures (**12**–**20**) were obtained in high yields (74–98%) with a simple two-step synthesis strategy and fully characterized. These compounds were assessed for their influence on various metabolic enzymes including human carbonic anhydrase isoenzymes (hCA I and hCA II), acetylcholinesterase (AChE), and butyrylcholinesterase (BChE). The *K_i_* values for the compounds concerning hCA I, hCA II, AChE, and BChE enzymes were in the ranges 29.94–121.69, 17.72–89.42, 14.09–44.68, and 1.15–48.82 nM, respectively. Compound **13** was 49.70-fold more active than tacrine (standard drug) for BChE and 5.49-fold for AChE. Compound **14** was 4.16-fold more active than acetazolamide (standard drug) for hCA I and 5.79-fold for hCA II. The cytotoxic effects of the synthesized click products were investigated on human triple-negative breast cancer cell lines. The IC_50_ values of the most effective compounds were calculated between 12.51 ± 1.92 and 18.07 ± 2.14 μM in MDA-MB-231 and BT-549 cells. Molecular docking and ADME predictions were performed. Then, *in vitro* effective compounds were analyzed by molecular dynamics (MD) simulation and MM/GBSA calculation. Consequently, click products showed good cytotoxicity and inhibition potential on colony formation in cancer cells.

## 1 Introduction

Zinc metalloproteins, known as carbonic anhydrases (CAs) play a crucial role in converting carbon dioxide into bicarbonate, simultaneously releasing a proton. Their involvement extends to the transfer of CO_2_ and protons across biological membranes, contributing to diverse functions such as bone resorption, fluid secretion, pH regulation, and ion transport.^1−3^ Various illnesses have been linked to the overexpression of specific CA isoforms in humans. Conditions such as brain edema and retinal, tumor development, altitude sickness, glaucoma, and epilepsy have been associated with the hCA I and hCA II isoforms.^4^

Reducing bicarbonate-dependent depolarization through the inhibition of CAs has been shown to alleviate negative effects in cases where the function of the neuron-specific potassium-chloride cotransporter was compromised after nerve injury.^5^ For instance, inhibiting spinal CA activity, as demonstrated by using acetazolamide (AZA), led to a reduction in neuropathic allodynia.^6^ This CA inhibitor exhibits a preferable capacity against the cytosolic isoform CAs. Consequently, animal model studies have identified isoform-selective inhibitors targeting CAs as potential drug candidates for addressing several disorders.^7,8^ Simultaneously, other studies have provided insight into carbonic anhydrases (CAs) as potential novel targets for the therapy of Alzheimer’s disease (AD).^9,10^ The elevated amounts of CA II detected in both the central and peripheral systems indicate that the expression of CA II might potentially serve as a biomarker for cognitive problems.^11^

According to the 2023 report from AD International, over 55 million people worldwide are afflicted by AD, and the incidence is anticipated to dramatically rise, reaching 139 million by 2050.^12^ As a result, AD poses a significant threat to global health, presenting society with an immense challenge. In the brains of healthy adults, acetylcholinesterase (AChE) exhibits hydrolytic activity, constituting 80% of the cholinesterase (ChE) activity.^13,14^ Notably, its activity is 10 times greater than that of butyrylcholinesterase (BChE), which contributes the remaining 20%.^12^ This observation underscores the critical role played by AChE in the hydrolysis of acetylcholine under physiological conditions.^15^

Several drugs, including tacrine, donepezil, rivastigmine, and galantamine, have received approval from the US Food and Drug Administration for treating AD.^16^ However, these drugs, designed to modulate a single target, have limited efficacy in mitigating or halting AD progression due to the involvement of multiple systems and the complex pathogenesis of the disease. Prolonged use of these drugs is also associated with serious side effects, including loss of appetite, diarrhea, vomiting, and nausea, in the case of tacrine, hepatotoxicity.^17,18^ Consequently, the discovery of more effective agents holds significant value for the treatment of AD.

Cancer is a serious problem worldwide. Triple-negative breast cancer (TNBC) has a short survival and is a subtype of breast cancer.^19^ Due to the limitations of the treatment and molecular subtypes, there is needed development of new drug candidates. On the other hand, the biological functions of the targets are regulated by binding of small molecules to the active site. It resulted in the discovery of new and potent drug candidates for targeted cancer therapies.^20^ The effectiveness of AChE inhibitors has been the subject of extensive research in the treatment of various diseases including cancer.^21,22^ For this purpose, many studies on AChE inhibitors in the literature contain their traditional relationships with neurodegenerative diseases as well as cancer treatment and other therapeutic applications.^4,23^ This highlights the importance of various compounds being investigated for their AChE inhibitor and potential effects in cancer therapy. At the same time, several studies indicate the role of hCAs in human diseases such as cancer and drug discovery.^24−26^

Heterocyclic compounds containing nitrogen atoms are used in various applications, such as the pharmaceutical industry and agricultural chemicals research due to their important physical, chemical, and biological properties. Due to their biological and pharmacological activities and structural diversity, these structures have become interesting targets for synthetic chemists.^27−29^

In particular, 5*H*-dibenzo[*b*,*f*]azepine (iminostilbene) skeleton is an important heterocyclic compound formed by the fusion of two benzene rings to the seven-membered central azepine ring.^30^ It has been reported in the literature that dibenzoazepine and its analogues exhibit various pharmacological activities, including antiallergic, antihistamine, spasmolytic, serotonin antagonistic, anticonvulsive, antiemetic, antiepileptic, anti-inflammatory, sedative, fungicidal, antimicrobial, and antioxidant.^31,32^ However, there are a limited number of studies in the literature to investigate the anticancer potential of dibenzoazepine-based compounds.^33^ As far as we can see, there are no inhibition studies of dibenzoazepine-based cholinesterase and carbonic anhydrase enzymes in the literature.

On the other hand, the triazole skeleton has broad implications from drug development to chemical biology and from materials chemistry to catalysis.^34^ When this unit is combined with other heterocyclic rings, more effective hybrid structures with high biological and pharmacological activity can emerge.^35^ 1,2,3-Triazole units can form noncovalent interactions with DNA and other cell biomolecules, such as hydrogen bonds, hydrophobic interactions, van der Waals forces, and dipole–dipole interactions. This situation is important for biological activity.^36^

To identify and discover possible drug candidates, *in silico* approaches are used. Thus, it is important to brighten interactions between molecules and understand their binding ability. To discover valuable drug candidates, combined techniques including molecular modeling are used. For this purpose, many therapeutics have been found by using *in silico* methods.^37,38^

The most efficient and green route for the synthesis of 1,2,3-triazoles is copper-catalyzed azide–alkyne cycloaddition (CuAAC), a click reaction. The CuAAC reaction has played an important role in synthetic organic chemistry because of short reaction times, excellent reaction yields, insensitivity to moisture, air, or water, moderate/simple reaction conditions, *etc.* This reaction is chemo- and regiospecific, atom-economical, and highly efficient, and the resulting triazole product can be easily isolated.^39^ In addition, the click reaction is one of the most commonly used synthetic strategies to combine two important bioactive scaffolds into a single scaffold by a molecular hybridization approach. Among numerous strategies to design new drugs, this molecular hybridization approach is an effective and popular approach based on combining two independent bioactive compounds into a new structure. In this approach, two or more chemical groups with known biological or pharmacological activities are linked together through chemical reactions. As a result, a new compound emerges that can exhibit the activity of each active group in a single hybrid structure.^40−42^

**Figure 1:**
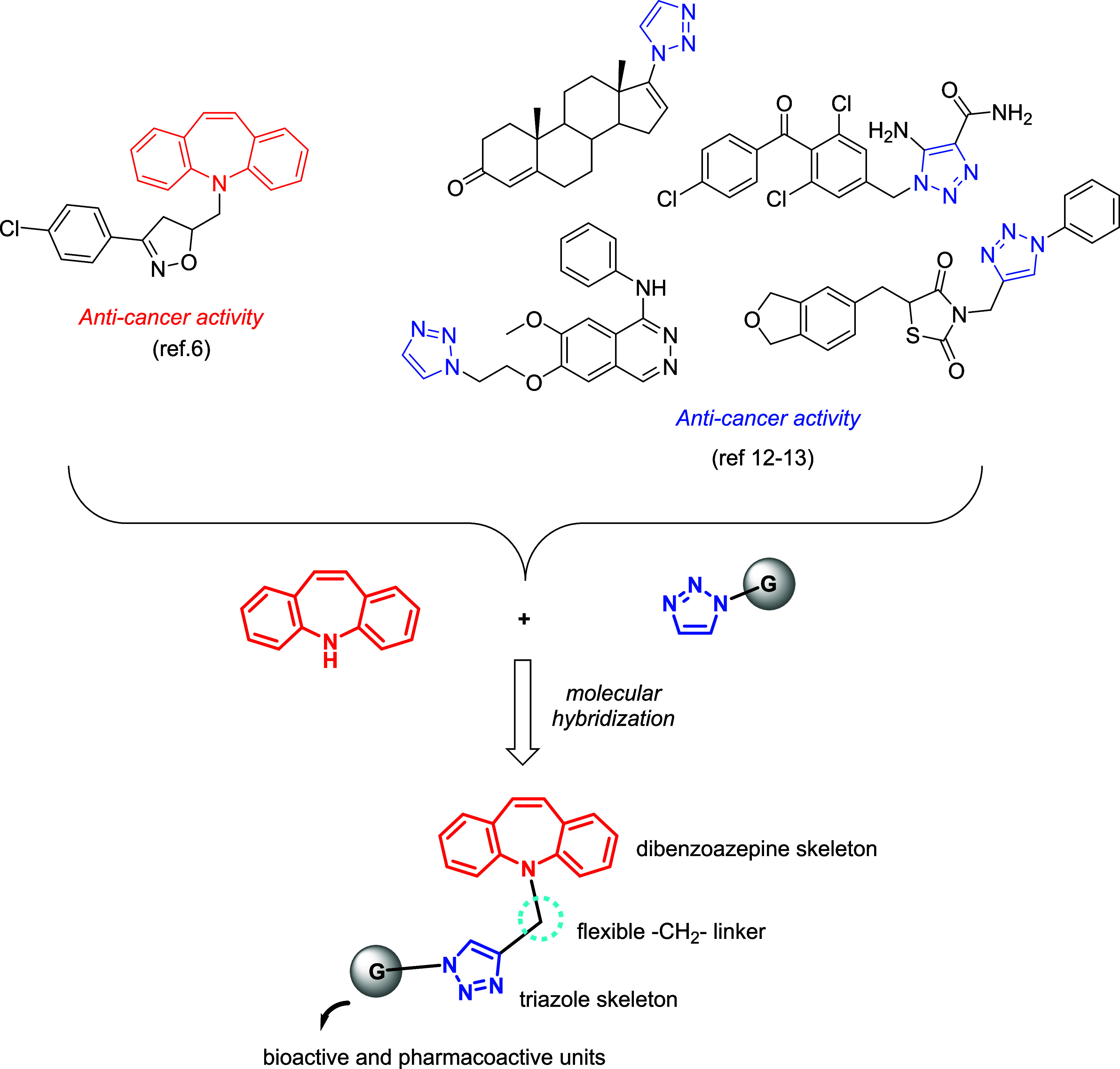
Design of target compounds by a molecular hybridization approach.

In this respect, herein we report the synthesis of dibenzoazepine (5*H*-dibenzo[*b*,*f*]azepine **1**) and 1,2,3-triazoles with several functionalities, including 7-ethoxy-coumaroyl, benzyl, 1-naphthyl, 3-pyridinyl, *p*-OMe-phenyl, *p*-SO_2_NH_2_-phenyl, *p*-NO_2_-phenyl, 3,4,5-trimethoxyphenyl, and 2-OH-phenyl, *via* click chemistry and molecular hybridization approach (Figure 1).

In this study, a set of innovative dibenzoazepine-substituted triazole hybrids were synthesized with the aim of identifying novel compounds showcasing improved activity against certain metabolic enzymes. The inhibitory effects of the newly prepared compounds were evaluated on hCA I and hCA II using esterase activity and on AChE/BChE activity using Ellman’s approach. Furthermore, triazole-linked dibenzoazepine hybrids have been successfully evaluated for their anticancer activities in TNBC cell lines. Within the scope of our study, *in vitro* effective compounds have been identified for their enzyme inhibition potentials by theoretical calculations including molecular docking, molecular dynamics (MD) simulation, molecular mechanics general Born surface area (MM/GBSA), and also ADME prediction.

## 2 Materials and Methods

### 2.1 General

The reactions were monitored by thin-layer chromatography (TLC) which was carried out on silica gel 60 HF254 aluminum plates (Fluka). Column chromatography was performed on silica gel (60 mesh, Merck). Melting points are uncorrected. ^1^H and ^13^C NMR spectra were recorded on a Bruker Ultrashield Plus Biospin (GmbH NMR) spectrometer, using TMS as the internal reference. All spectra were recorded at 25 °C and coupling constants (*J* values) are given in hertz. Chemical shifts are given in parts per million (ppm). Mass spectra were recorded on an Agilent Technologies 6530 Accurate-Mass Q-TOF-LC/MS. Infrared (IR) spectra were recorded on a PerkinElmer FT-IR spectrometer.

### 2.2 Synthesis and Characterization

#### 2.2.1 Preparation of Intermediates **2, 3–11**

5-(Prop-2-yn-1-yl)-5*H*-dibenzo[*b*,*f*]azepine (**2**)^43^ and the substituted azide derivatives **3**–**11** were prepared as in our previous study.^44^

#### 2.2.2 Preparation of the Click Products **12**–**20**

The propargyl derivative **2** (1.00 mmol), the substituted alkyl or aromatic azides **3**–**11** (1.10 mmol, 1.1 equiv), CuSO_4_·5H_2_O (0.20 mmol), and sodium-l-ascorbate (0.40 mmol) were dissolved in 45 mL of THF–water (2:1, v/v%, mL). A catalytic amount of NEt_3_ (3 drops) was added to the mixture. The reaction mixture was stirred at reflux for approximately 6–12 h until TLC indicated the reaction was complete. THF was then evaporated under reduced pressure, and the mixture was extracted with DCM or EtOAc.^45^ The crude products were purified by column chromatography on silica gel with EtOAc and petroleum ether (20–40% EtOAc in petroleum ether) to obtain click products **12**–**20**.

##### 2.2.2.1 5-((1-Benzyl-1*H*-1,2,3-triazol-4-yl)methyl)-5*H*-dibenzo[*b*,*f*]azepine (**12**)

Light yellow solid, yield 95%, mp 147–149 °C (lit.^45^ 90 °C), ^1^H NMR (400 MHz, CDCl_3_) δ 7.35–7.19 (m, 6H), 7.18–7.05 (m, 4H), 7.01 (t, *J* = 7.4 Hz, 2H), 6.92 (dd, *J* = 7.4, 1.3 Hz, 2H), 6.75 (s, 2H), 5.39 (s, 2H), 5.14 (m, 2H). ^13^C NMR (100 MHz, CDCl_3_) δ: 149.93, 146.26, 134.99, 133.56, 132.05, 129.14, 129.03, 128.89, 128.28, 127.21, 123.76, 122.94, 120.59, 53.79, 47.46. FTIR (cm^–1^): 3060, 3017, 2955, 2906, 2863, 1956, 1589, 1572, 1483, 1458, 1433, 1321, 1225, 1117, 1045, 905, 874, 798, 768, 709. HRMS (Q-TOF): *m*/*z* [M + H]^+^ calcd. for C_24_H_21_N_4_: 365.17662; found: 365.17606.

##### 2.2.2.2 5-((1-(Naphthalen-1-yl)–1*H*-1,2,3-triazol-4-yl)methyl)-5*H*-dibenzo[*b*,*f*]azepine (**13**)

Bronze solid, yield 97%, mp 76–78 °C, ^1^H NMR (400 MHz, CDCl_3_) δ 7.94 (dd, *J* = 7.5, 1.5 Hz, 1H), 7.89 (d, *J* = 8.3 Hz, 1H), 7.59 (s, 1H), 7.55–7.45 (m, 3H), 7.34 (ddd, *J* = 8.3, 6.9, 1.2 Hz, 1H), 7.31–7.23 (m, 2H), 7.19–7.12 (m, 2H), 7.08 (dd, *J* = 7.6, 1.7 Hz, 2H), 7.02 (td, *J* = 7.4, 1.1 Hz, 2H), 6.83 (dd, *J* = 8.6, 0.7 Hz, 1H), 6.72 (s, 2H), 5.28 (s, 2H). ^13^C NMR (100 MHz, CDCl_3_) δ 150.09, 145.98, 134.03, 133.80, 133.77, 132.14, 130.18, 129.21, 129.12, 128.51, 128.15, 127.73, 126.88, 125.67, 124.96, 123.77, 123.49, 121.97, 120.64, 47.51. FTIR (cm^–1^): 3145, 3052, 3020, 2850, 1595, 1574, 1485, 1459, 1436, 1376, 1327, 1234, 1117, 1037, 1016, 905, 864, 789, 765, 715. HRMS (Q-TOF): *m*/*z* [M + H]^+^ calcd. for C_27_H_21_N_4_: 401.17662; found: 401.17639.

##### 2.2.2.3 5-((1-(Pyridin-3-yl)-1*H*-1,2,3-triazol-4-yl)methyl)-5H-dibenzo[*b*,*f*]azepine (**14**)

Beige solid, yield 93%, mp 93–95 °C, ^1^H NMR (400 MHz, CDCl_3_) δ 8.84 (s, 1H), 8.64 (d, *J* = 4.0 Hz, 1H), 8.01 (ddd, *J* = 8.3, 2.5, 1.4 Hz, 1H), 7.74 (s, 1H), 7.43 (dd, *J* = 8.2, 4.8 Hz, 1H), 7.33–7.22 (m, 2H), 7.23–7.08 (m, 4H), 7.03 (t, *J* = 7.4 Hz, 2H), 6.84 (s, 2H), 5.24 (s, 2H). ^13^C NMR (100 MHz, CDCl_3_) δ: 149.86, 149.55, 147.42, 141.32, 133.70, 133.60, 132.14, 129.26, 129.19, 128.00, 124.15, 123.99, 120.49, 120.39, 47.28. FTIR (cm^–1^):3126, 3085, 3031, 2851, 2839, 1592, 1558, 1487, 1457, 1373, 1353, 1299, 1242, 1229, 1178, 1117, 1040, 1022, 987, 947, 908, 852, 801, 793, 763, 748, 703. HRMS (Q-TOF): *m*/*z* [M]^+^ calcd. for C_22_H_17_N_5_: 351.14840; found: 351.14990.

##### 2.2.2.4 5-((1-(4-Methoxyphenyl)-1*H*-1,2,3-triazol-4-yl)methyl)-5H-dibenzo[*b*,*f*]azepine (**15**)

Light yellow solid, yield 88%, mp 166–168 °C, ^1^H NMR (400 MHz, CDCl_3_) δ 7.55 (s, 1H), 7.48–7.40 (m, 2H), 7.27–7.19 (m, 2H), 7.12 (d, *J* = 7.5 Hz, 2H), 7.08 (dd, *J* = 7.6, 1.6 Hz, 2H), 7.00–6.90 (m, 4H), 6.79 (s, 2H), 5.18 (s, 2H), 3.81 (s, 3H). ^13^C NMR (100 MHz, CDCl_3_) δ: 159.69, 150.18, 146.59, 133.60, 132.15, 130.65, 129.16, 129.12, 123.79, 122.08, 120.67, 120.41, 114.65, 55.61, 47.39. FTIR (cm^–1^): 3127, 3084, 3013, 2971, 2938, 2840, 1592, 1557, 1519, 1485, 1455, 1434, 1372, 1317, 1275, 1261, 1240, 1222, 1191, 1172, 1116, 1048, 1034, 823, 794, 767, 750. LC MS/MS: *m*/*z* [M + Na]^+^ calcd. for C_24_H_20_N_4_NaO: 403.15; found: 403.20.

##### 2.2.2.5 7-(2-(4-((5*H*-Dibenzo[*b*,*f*]azepin-5-yl)methyl)-1*H*-1,2,3-triazol-1-yl)ethoxy)-2H-chromen-2-one (**16**)

Beige solid, yield 75%, mp 167–169 °C, ^1^H NMR (400 MHz, CDCl_3_) δ 7.62 (d, *J* = 9.5 Hz, 1H), 7.41–7.30 (m, 2H), 7.23–7.13 (m, 2H), 7.11–6.99 (m, 4H), 6.93 (td, *J* = 7.4, 1.0 Hz, 2H), 6.77 (s, 2H), 6.68–6.62 (m, 2H), 6.27 (d, *J* = 9.5 Hz, 1H), 5.11 (s, 2H), 4.61 (t, *J* = 5.0 Hz, 2H), 4.24 (t, *J* = 5.0 Hz, 2H). ^13^C NMR (100 MHz, CDCl_3_) δ: 160.81, 160.74, 155.68, 150.14, 146.34, 143.11, 133.55, 132.12, 129.11, 129.04, 128.88, 123.71, 123.48, 120.34, 113.83, 113.28, 112.27, 102.01, 66.75, 49.24, 47.34. FTIR (cm^–1^): 3135, 3069, 3006, 2990, 2965, 2902, 2851, 1722, 1612, 1485, 1453, 1392, 1354, 1275, 1230, 1212, 1124, 1052, 1037, 1000, 898, 844, 765, 751. HRMS (Q-TOF): *m*/*z* [M + H]^+^ calcd. for C_28_H_23_N_4_O_3_: 463.17702; found: 463.17645.

##### 2.2.2.6 4-(4-((5*H*-Dibenzo[*b*,*f*]azepin-5-yl)methyl)-1H-1,2,3-triazol-1-yl)benzenesulfonamide (**17**)

Light yellow solid, yield 76%, mp 117–119 °C, ^1^H NMR (400 MHz, DMSO-*d*_6_) δ 8.61 (s, 1H), 8.03 (d, *J* = 8.8 Hz, 2H), 7.97 (d, *J* = 8.7 Hz, 2H), 7.51 (s, 2H), 7.32–7.24 (m, 4H), 7.11 (d, *J* = 7.6 Hz, 2H), 7.07–6.92 (m, 2H), 6.80 (s, 2H), 5.09 (s, 2H). ^13^C NMR (100 MHz, DMSO-*d*_6_) δ: 149.84, 145.85, 143.73, 138.43, 133.18, 132.00, 128.92, 128.84, 127.47, 123.55, 122.02, 120.56, 120.03, 45.81. FTIR (cm^–1^): 3355, 3250, 3006, 2988, 1716, 1592, 1486, 1459, 1331, 1277, 1259, 1156, 1040, 903, 767, 749. LC MS/MS: *m*/*z* [M – H]^+^ calcd. for C_23_H_18_N_5_O_2_S: 428.12; found: 428.25.

##### 2.2.2.7 5-((1-(4-Nitrophenyl)-1*H*-1,2,3-triazol-4-yl)methyl)-5*H*-dibenzo[*b*,*f*]azepine (**18**)

Light brown solid, yield 91%, mp 161–163 °C, ^1^H NMR (400 MHz, CDCl_3_) δ 8.30 (d, *J* = 9.0 Hz, 2H), 7.76 (d, *J* = 9.0 Hz, 2H), 7.75 (s, 1H), 7.30–7.20 (m, 2H), 7.17–7.05 (m, 4H), 6.99 (t, *J* = 7.4 Hz, 2H), 6.81 (s, 2H), 5.20 (s, 2H). ^13^C NMR (100 MHz, CDCl_3_) δ: 149.88, 147.88, 147.87, 147.06, 141.15, 133.60, 132.14, 129.27, 129.23, 125.36, 124.01, 120.37, 120.31, 47.21. FTIR (cm^–1^): 3093, 3014, 2988, 2844, 1596, 1519, 1503, 1487, 1458, 1336, 1276, 1259, 1232, 1116, 1108, 1039, 1021, 854, 765, 749. HRMS (Q-TOF): *m*/*z* [M + H]^+^ calcd. for C_23_H_18_N_5_O_2_: 396.14605; found: 396.14548.

##### 2.2.2.8 5-((1-(3,4,5-Trimethoxyphenyl)-1*H*-1,2,3-triazol-4-yl)methyl)-5*H*-dibenzo[*b*,*f*]azepine (**19**)

Light yellow solid, yield 98%, mp 133–135 °C, ^1^H NMR (400 MHz, CDCl_3_) δ 7.60 (s, 1H), 7.29–7.20 (m, 2H), 7.13 (dd, *J* = 8.0, 0.7 Hz, 2H), 7.08 (dd, *J* = 7.6, 1.6 Hz, 2H), 6.98 (td, *J* = 7.4, 1.1 Hz, 2H), 6.79 (s, 2H), 6.77 (s, 2H), 5.18 (s, 2H), 3.87 (s, 6H), 3.84 (s, 3H). ^13^C NMR (100 MHz, CDCl_3_) δ: 153.83, 150.08, 146.66, 138.41, 133.59, 132.97, 132.13, 129.15 (2C), 123.82, 120.92, 120.43, 98.76, 61.01, 56.46, 47.32. FTIR (cm^–1^): 3158, 2987, 2957, 2930, 2831, 1603, 1510, 1458, 1304, 1228, 1122, 1069, 1045, 1007, 936, 865, 817, 800, 765, 716. HRMS (Q-TOF): *m*/*z* [M + H]^+^ calcd. for C_26_H_25_N_4_O_3_: 441.19267; found: 441.19238.

##### 2.2.2.9 2-(4-((5*H*-Dibenzo[*b*,*f*]azepin-5-yl)methyl)-1*H*-1,2,3-triazol-1-yl)phenol (**20**)

Brown solid, yield 74%, mp 99–101 °C, ^1^H NMR (400 MHz, DMSO-*d*_6_) δ 10.50 (bs, 1H), 8.09 (s, 1H), 7.55 (dd, *J* = 7.9, 1.0 Hz, 1H), 7.34–7.19 (m, 5H), 7.11 (d, *J* = 7.3 Hz, 2H), 7.05 (d, *J* = 8.0 Hz, 1H), 7.03–6.97 (m, 2H), 6.92 (t, *J* = 7.6 Hz, 1H), 6.79 (s, 2H), 5.10 (s, 2H). ^13^C NMR (100 MHz, DMSO-*d*_6_) δ 150.08, 148.88, 133.17, 131.98, 129.71, 128.89, 124.77, 124.74, 124.70, 124.37, 124.30, 123.49, 120.54, 119.55, 117.10, 46.09. FTIR (cm^–1^): 3187, 3064, 3011, 2840, 2795, 2745, 2623, 1736, 1594, 1572, 1476, 1459, 1436, 1332, 1278, 1258, 1234, 1117, 1060, 991, 932, 904, 855, 833, 816, 771, 745, 716. HRMS (Q-TOF): *m*/*z* [M]^+^ calcd. for C_23_H_18_N_4_O: 366.14806; found: 366.14562.

### 2.3 Activity of CAs, AChE, and BChE

The inhibition effects of the compounds *versus* the esterase activity of the hCAs were determined by following the change in absorbance at 348 nm according to the assay defined by Verpoorte et al.^46^ hCAs activities were measured using 4-nitrophenyl acetate as in a previous study.^47^ All of the measurements were repeated thrice. AChE and BChE activity were assessed using a modified version of the Ellman method.^48^ The measurement of AChE/BChE activity was conducted with acetylthiocholine iodide/butyrylthiocholine iodide as substrates along with 5,5-dithiobis (2-nitrobenzoic) acid. Substrate utilization was monitored spectrophotometrically at 412 nm.^49^

### 2.4 *In Vitro* Enzyme Inhibition Studies

The inhibitory effects of the newly synthesized compounds were assessed using a minimum of five different inhibitor concentrations for hCAs and AChE/BChE. The IC_50_ values for these synthesized derivatives were determined from the graphs of Activity (%) *versus* [Synthesized derivatives] for each compound.^49,50^ Inhibition types and *K_i_* values were determined using Lineweaver and Burk’s curves.

### 2.5 *In Vitro* Anticancer Activity

Two different cell lines were used in this study. TNBC cell lines (MDA-MB-231 and BT-549) were a gift from Erciyes University (Türkiye). The cells were cultured in DMEM/F12, 10% FBS, 1% PS, and incubated under 5% CO_2_ in an incubator.^44,51^

#### 2.5.1 MTT Cytotoxicity Assay

MTT reagent was used to determine the cytotoxicity of the synthesized compounds for 72 and 96 h treatments. The same protocol was given in our previously reported studies.^44,51^

#### 2.4.2 Colony Formation Inhibition Assay

Colony inhibition assay was performed in a 24-well plate for each compound in increasing concentrations according to our reported study.^44^

#### 2.5.3 Statistical Analysis

GraphPad Prism 8.0 was used for the statistical analysis. *P*-values less than 0.05 were considered statistically significant and indicated with an asterisk.

### 2.6 *In Silico* Studies

#### 2.6.1 ADME Prediction

*In silico* prediction of pharmacokinetic and physicochemical properties (ADME) of the synthesized compounds was performed by using QikProp module of Maestro (Schrödinger Release 2023–1).^44^ The compounds were drawn by ChemDraw and used as.sdf.

#### 2.6.2 Molecular Docking

Molecular docking studies were carried out by the Glide/SP method of Maestro (Schrödinger Release 2023–1). The crystal structures of hCA I (PDB: 2FW4), hCA II (PDB: 1G45), hAChE (PDB: 4EY7), hBChE (PDB: 6I0C), SphK1 (PDB: 4V24), and CDK6 (PDB: 5L2I) were downloaded from RCSB (https://www.rcsb.org/). Protein and ligand preparation and docking protocol were performed according to our previous studies.^44,51,52^

#### 2.6.3 MD Simulations and MM/GBSA Calculations

MD simulation studies were performed by using the Desmond module of Maestro (Schrödinger Release 2023–1) with top docking poses of the *in vitro* effective compounds for a period of 100 ns simulations. The protocol was adjusted under default settings given our recent studies.^44,51,52^ Prime module was used in Maestro to determine binding free energies of all 10 ns trajectories.^52^

## 3 Results

### 3.1 Chemistry

**Scheme 1:**
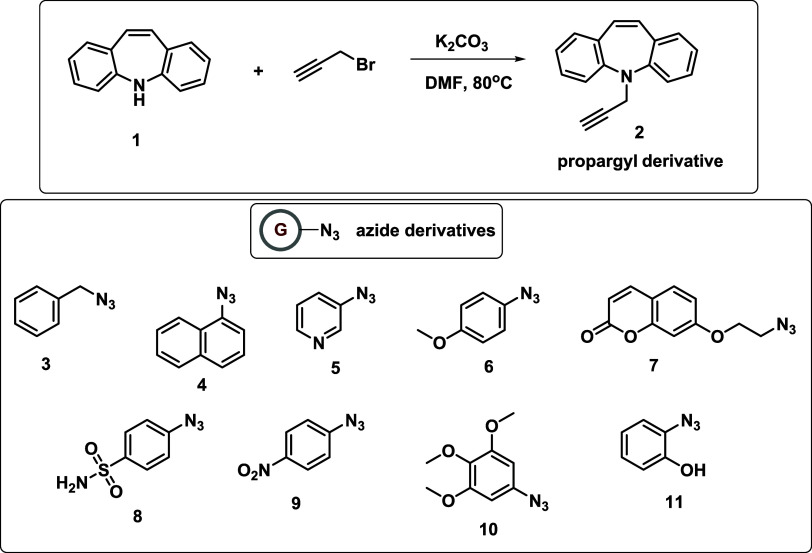
Synthesis of Propargyl Derivative **2** and Structures of Azide Derivatives **3**–**11**

**Scheme 2:**
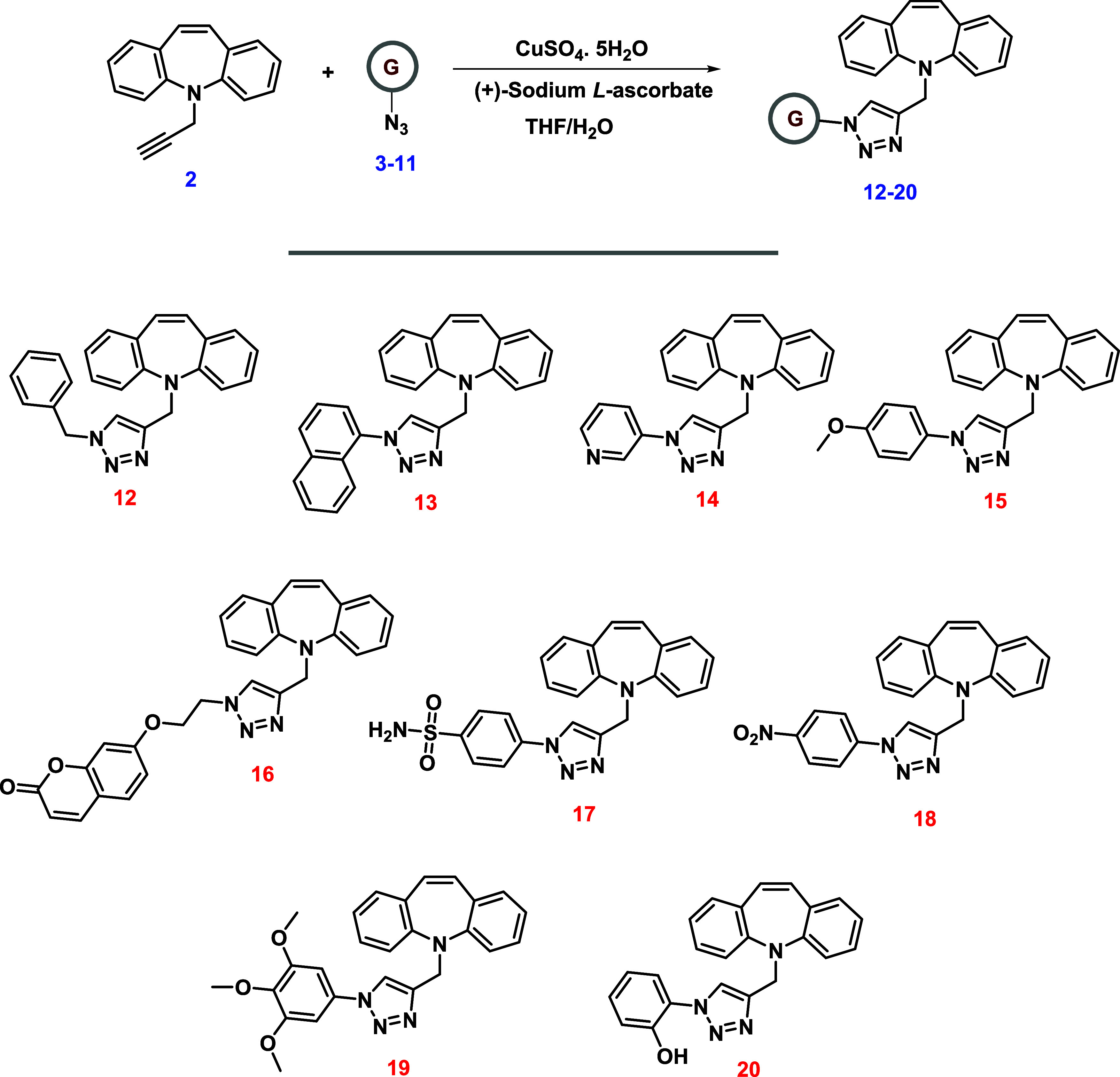
Synthesis of Dibenzoazepine-triazole Hybrid Derivatives **12**–**20**

The dibenzoazepine-substituted triazole hybrid derivatives **12**–**20** were synthesized with a two-step process consisting of a nucleophilic substitution reaction and Cu-catalyzed azide–alkyne 1,3-dipolar cycloaddition reaction (CuAAC). The synthesis of propargyl derivative and targeted Click products **12**–**20** is shown in Schemes 1 and 2. The azide intermediate structures used in the click reaction are given in Scheme 1. First, KDBA salt was obtained *in situ* by treating DBA with K_2_CO_3_ in anhydrous DMF. The propargyl derivative **2** was synthesized by adding propargyl bromide to this mixture after 45 min.^43^ Subsequently, 9 different azide derivatives were synthesized using the methods in our previous work.^44^ Two key azides **3** and **7** were prepared *via* nucleophilic substitution reaction of their benzyl bromide and 7-(2-bromoethoxy)-2*H*-chromen-2-one precursors, respectively, using NaN_3_ in DMF or acetone.^44^ Other aromatic azides **4**–**6** and **8**–**11** were obtained from their commercially available amine derivatives using the classical Sandmeyer reaction.^44^ Finally, dibenzoazepine-triazole hybrid derivatives were produced by the click chemistry reactions of propargyl derivative **2** and 9 different substituted azide derivatives **3**–**11**. Click reactions were initially performed at room temperature and monitored by thin-layer chromatography (TLC). It was observed that the yields of the reactions increased when the reaction temperature was increased (refluxed temperature), and the catalytic amount of triethylamine (NEt_3_) was added to the mixture. Structural characterizations of the click products were performed using standard spectroscopic techniques such as ^1^H and ^13^C NMR as well as high-resolution mass spectrometry (HRMS) and Fourier transform infrared (FT-IR) spectroscopy.

As a result of the click reactions, the signal of the acetylene proton at 2.21 ppm of propargyl derivative **2** disappeared in the ^1^H NMR spectrum, while the triazole ring proton (C–H) showed a characteristic signal in the range of 7.20–8.61 ppm (1H, s, triazole ring). In addition, the signals of the −CH_2_– bridge connecting the dibenzoazepine ring and the triazole ring appeared between 5.28 and 5.09 ppm. Furthermore, the FTIR spectra of Click products **12**–**20** did not show the azido group vibration band found in the precursor azides. In ^13^C NMR, the carbon signals of all compounds resonated in the expected regions, consistent with their structures. Furthermore, HRMS or LC-MS/MS confirmed the integrity of the obtained Click products, since in all cases the molecular ions conformed to the proposed structures.

### 3.2 Enzyme Inhibition Studies

**Figure 2:**
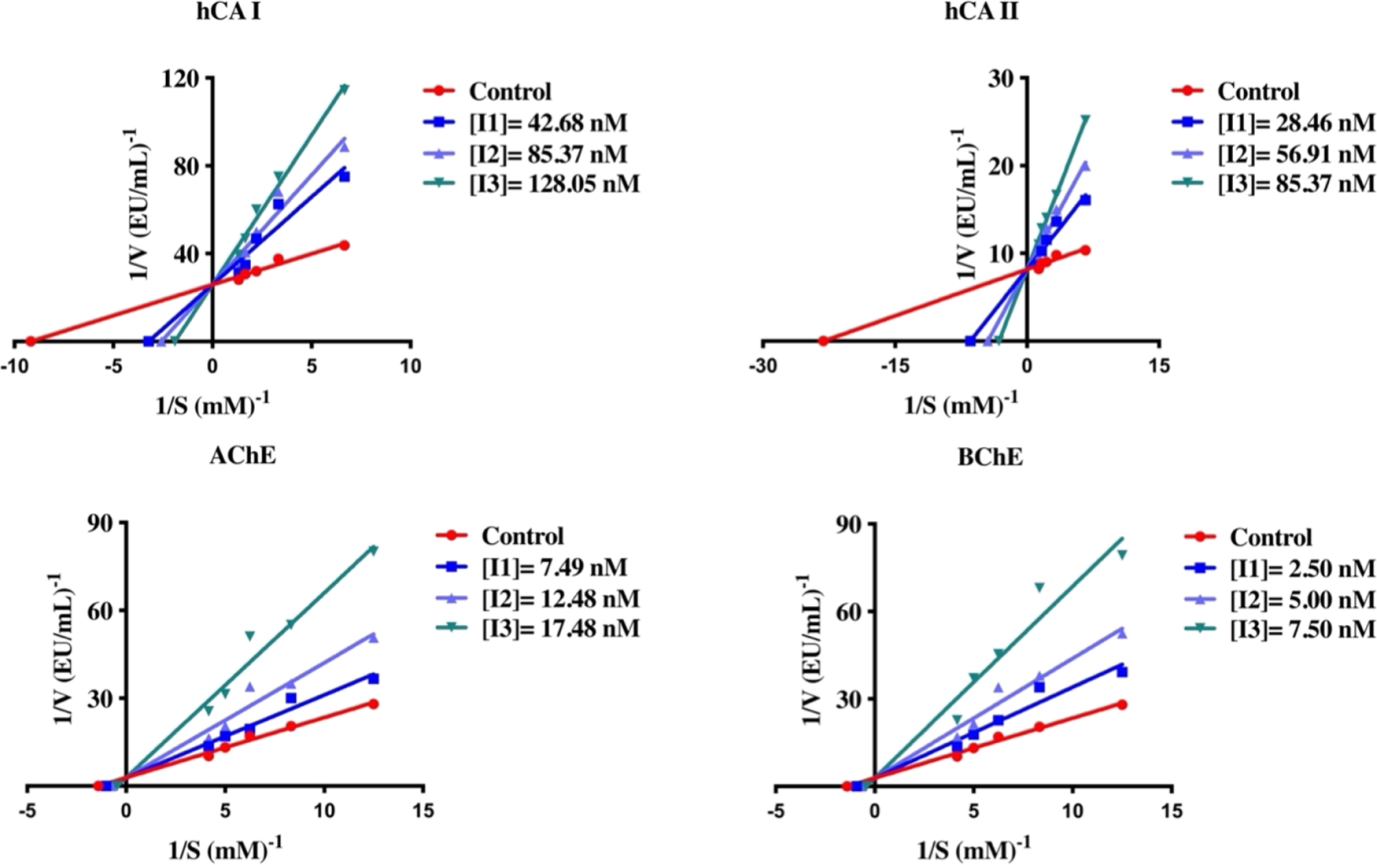
Lineweaver–Burk plots of the compound showing the best inhibition in newly synthesized dibenzoazepine-substituted triazole hybrids (**12**–**20**) (**14** for hCA I and hCA II; **13** for AChE and BChE).

All of the 9 newly synthesized dibenzoazepine-substituted triazole hybrid compounds **12**–**20** were tested against two CA isoforms, the cytosolic hCA I and hCA II, applying the esterase assay AZA as the standard drug.^53,54^ In addition, the effects of these compounds on the activity of the cholinesterase enzymes AChE and BChE were determined using the Ellman method, and TAC was used as the standard drug. The inhibition data is summarized in Table 1 and Figure 2.

**Table 1:** Inhibition Data of hCA I, II Isoforms, AChE, and BChE with the Novel Dibenzoazepine-Substituted Triazole Hybrids (**12**–**20**)

	*K*_i_ (nM)			
compounds	hCA I	hCA II	AChE	BChE
**12**	48.84 ± 4.68	40.52 ± 5.85	28.94 ± 3.52	48.82 ± 8.62
**13**	62.05 ± 8.53	28.78 ± 4.64	14.09 ± 3.81	1.15 ± 0.15
**14**	29.94 ± 5.93	17.72 ± 1.54	21.21 ± 5.87	7.65 ± 1.49
**15**	54.73 ± 9.63	39.09 ± 2.17	24.12 ± 0.45	4.11 ± 0.46
**16**	121.69 ± 20.16	54.58 ± 1.99	17.49 ± 2.19	11.24 ± 1.55
**17**	40.49 ± 11.07	56.65 ± 11.45	19.02 ± 5.44	15.26 ± 1.12
**18**	118.39 ± 20.95	69.67 ± 5.93	44.68 ± 9.61	39.15 ± 8.82
**19**	93.97 ± 15.54	61.44 ± 9.11	34.46 ± 8.54	2.22 ± 0.17
**20**	80.40 ± 15.63	89.42 ± 4.92	22.83 ± 4.78	16.22 ± 2.43
**AZA**	124.55 ± 8.11	102.56 ± 7.34		
**TAC**			77.41 ± 3.12	57.16 ± 6.45

Compound **14** incorporating 1-(pyridin-3-yl) moiety showed the highest inhibitory effect against hCA I. Modification or replacement of this moiety to 1-(4-nitrophenyl), sharply decreases the activity of hCA I. As we noticed in the inhibition data against hCA I, compounds **14**, **17**, **12**, and **15** showed *K*_Is_ of 29.94, 40.49, 48.84, and 54.73 nM, respectively. Also, compounds bearing 2-(4-((5*H*-dibenzo[*b*,*f*]azepin-5-yl)methyl)-1*H*-1,2,3-triazol-1-yl)ethoxy showed a sharp decrease in the inhibition effect against hCA I with a *K_i_* of 121.69 nM. The hCA I enzyme inhibition effects of the groups attached to the 5H-dibenzo[*b*,*f*]azepine group are examined as follows: 1-(pyridin-3-yl) > 1-benzyl >1-(4-methoxyphenyl) > 1-(naphthalen-1-yl) > 1-(3,4,5-trimethoxyphenyl) > 1-(4-nitrophenyl). All of the compounds were more effective than AZA (*K_i_*: 124.55 ± 8.11 nM) for hCA I.

The **14** incorporating 1-(pyridin-3-yl) moiety was mainly active upon hCA II isoform, with a mean inhibition activity constant *K_i_* = 17.72 nM. The other compounds were mainly active against hCA II with a mean inhibition constant *K_i_* range of 28.78–89.42 nM. When the results were examined, 1-(naphthalen-1-yl) group showed approximately 1.41 times more inhibition effect than 1-benzyl group, while 1-(4-nitrophenyl) group showed 2.42 times more inhibition effect. In compounds **17** and **20**, the change in the positions to which the groups were attached caused a 1.58-fold change in hCA II enzyme activity (**17**, *K_i_*: 56.65 nM; **20**, *K_i_*: 89.42 nM). The substitution of 1-(4-methoxyphenyl) group instead of 1-benzyl group did not cause much change in the *K_i_* value (**12**, *K_i_*: 40.52 nM; **15**, *K_i_*: 39.09 nM). All of the compounds were more effective than AZA (*K_i_*: 102.56 ± 7.34 nM) for hCA II.

According to the results reported in Table 1, all compounds showed high potency against AChE with a *K_i_* value ranging from 14.09 to 44.68 nM. From the screening data, it was revealed that **13** having a 1-(naphthalen-1-yl) moiety showed better inhibition effect. The potency of studied compounds indicated the following order for AChE **13** (*K_i_*: 14.09 ± 3.81 nM, Figure 1) > **16** (*K_i_*: 17.49 ± 2.19 nM) > **17** (*K_i_*: 19.02 ± 5.44 nM) > **14** (*K_i_*: 21.21 ± 5.87 nM) > **20** (*K_i_*: 22.83 ± 4.78 nM) > **15** (*K_i_*: 24.12 ± 0.45 nM) > **12** (*K_i_*: 28.94 ± 3.52 nM) > **19** (*K_i_*: 34.46 ± 8.54 nM) > **18** (*K_i_*: 44.68 ± 9.61 nM). When the results were examined, 1-(pyridin-3-yl) group showed approximately 1.62 times more inhibition effect than 1-(3,4,5-trimethoxyphenyl), while the 1-(4-nitrophenyl) group showed 2.11 times more inhibition effect.

The inhibitory potential of the target compounds against BChE from equine serum was evaluated by using the Ellman method, with TAC serving as the positive reference standard. Table 1 demonstrates that all derivatives exhibited a significant inhibitory effect on the BChE activity. We found the 1-(naphthalen-1-yl)- and 1-(4-methoxyphenyl)-substituted exhibited greater inhibitory effect against BChE than with (1-benzyl) substitution. Looking at the results, compound **13** showed approximately 42.48 times more inhibition effect than compound **12**, and the compound **18** group showed 34.04 times more inhibition effect. Compared to AChE, the compounds inhibited the BChE enzyme better. Even compound **12**, which showed less inhibition effect compared to the others, showed a better inhibition effect than TAC (**12**, *K_i_*: 48.82 ± 8.62 nM; TAC, *K_i_*: 57.16 ± 6.45 nM).

### 3.3 *In Vitro* Cytotoxic Activity of the Synthesized Compounds

#### 3.3.1 Cell Cytotoxicity

**Figure 3:**
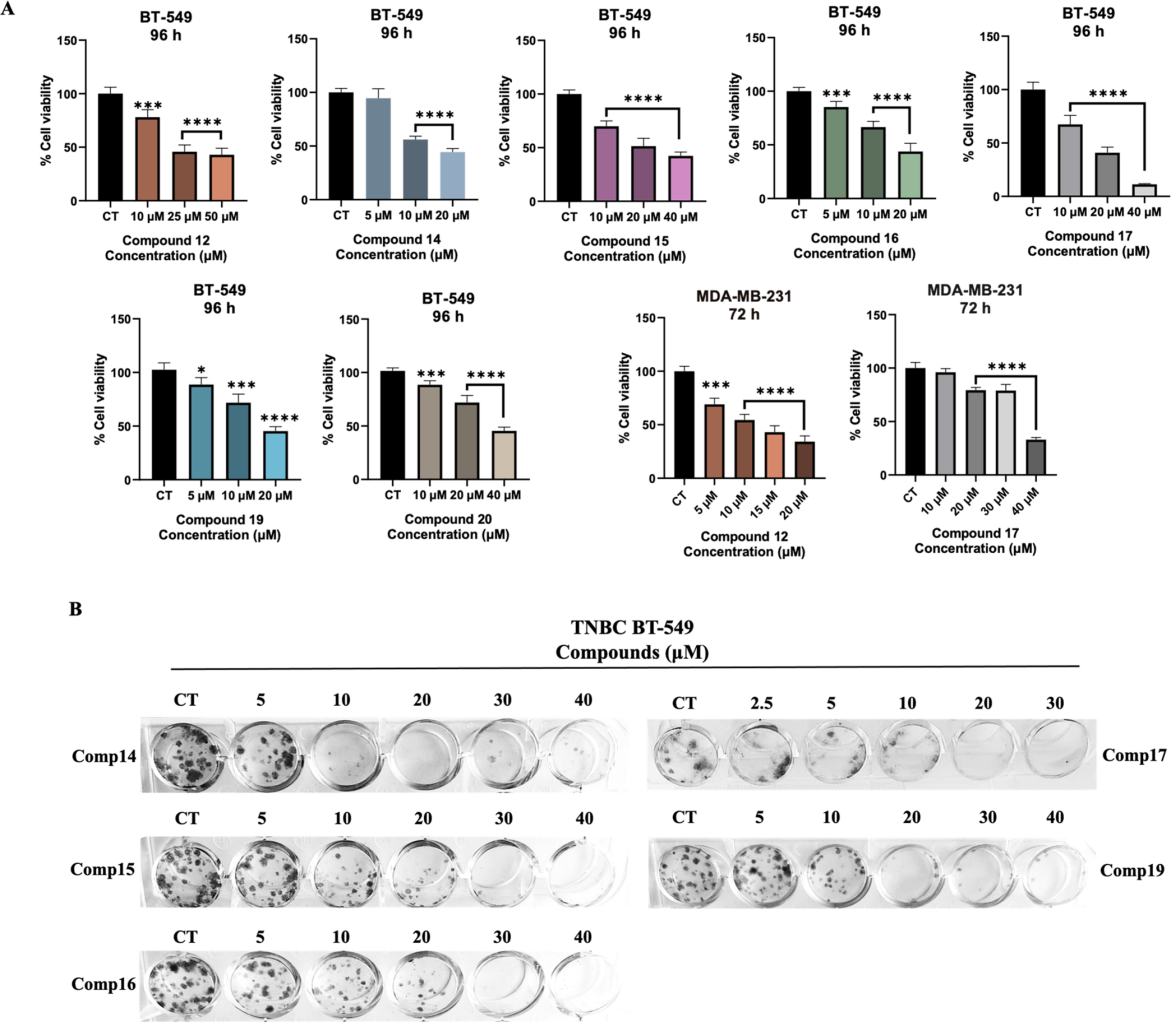
(A) *In vitro* cytotoxicity for the treatments of 96 and 72 h on TNBC BT-549 and MDA-MB-231 cell lines, respectively. (B) Colony formation inhibition profiles of the compounds on BT-549 cells in increasing concentrations.

All compounds were tested in terms of the cytotoxic activities against two TNBC cell lines, including MDA-MB-231 and BT-549. The IC_50_ values of the compounds are given in Table 2. According to these results, the highest cytotoxic activity was obtained with compound **14** at 16.59 ± 0.97 μM in BT-549 cells for 96 h treatments. It was followed by the compounds **17**, **16**, and **19** below <20 μM, respectively. As mentioned above, the compounds except of **13** and **18** decreased the percentage of cell viability in BT-549 cells at low concentrations between 16.59 and 36.16 μM (Figure 3a). In MDA-MB-231 cells, the IC_50_ values of the compounds **12** and **17** for 72 h treatments were found at 12.51 ± 1.92 and 36.50 ± 1.62 μM concentrations, respectively. Although the treatments with compounds **13**, **15**, **16**, and **18–20** were performed up to 125 and 150 μM, the cell viability did not reduce at low concentrations. Based on the IC_50_ values and due to their cytotoxic profiles, these tested compounds may be potential anticancer agents for targeted cancer therapy with further *in vitro* analysis.

**Table 2:** Cytotoxicity of the Synthesized Compounds in Cancer Cell Lines

	IC_50_ (μM)	
compounds	BT-549	MDA-MB-231
**12**	33.47 ± 3.03	12.51 ± 1.92
**13**	>40	>125
**14**	16.59 ± 0.97	>75
**15**	26.79 ± 4.60	>150
**16**	17.69 ± 2.54	>150
**17**	17.40 ± 3.09	36.50 ± 1.62
**18**	>40	>150
**19**	18.07 ± 2.14	>150
**20**	36.16 ± 3.77	>100

#### 3.3.2 Colony Inhibition Assay

The compounds were analyzed on TNBC BT-549 cells to determine their effects on colony formation inhibition. The cells were inhibited by the compounds **14**, **15**, **16**, **17**, and **19** in 7–10 days. The compounds **14, 15, 16, 17**, and **19** suppressed the colonies at 10 μM, 20 μM, and 30 μM concentrations (Figure 3b).

### 3.4 *In Silico* Studies

#### 3.4.1 *In Silico* ADME Prediction

According to *in silico* prediction of pharmacokinetic and drug-like properties of the synthesized click products, the predicted water/gas distribution coefficients were calculated in the expected range. The predicted octanol/water distribution coefficient, that is, the physicochemical properties of the parameter for the behavior of the synthesized compounds, are not in the recommended range.^55^ The predicted aqueous solubility was found at high values for compounds **13, 15, 16**, and **19** with −6.885, −6.592, −6.893, and −6.754, respectively. The predicted Caco-2 cell permeability was found as very good for the compounds except for compounds **17** and **18**. The predicted brain/blood distribution and skin permeability values were found in the expected range. The prediction of binding to human serum albumin value was calculated in the expected range for the compounds. Human oral absorption values are high for compounds **14, 17**, and **20**. The percentage of human oral absorption of the compounds is high. The compounds are acceptable for Lipinski’s rule of five (Table 3). The electrical activity of the heart is related to HERG K+ channels as a molecular target for cardiac toxicity. Therefore, these channel blockers are known as toxic, and the IC_50_ values are recommended in the expected value.^56,57^ The compounds may induce HERG-related toxicity. Optimum ranges for 95% of known drugs are used by this method.^58^ In summary, Caco-2 cell permeability describes intestinal absorption, and thus, appropriate human intestinal absorption and bioavailability values are recommended for the development of new drug candidates.^59^

**Table 3:** *In Silico* ADME Prediction

comp.	a*Q* log Pw	bQP log Po/w	cQP log *S*	dQP log HERG	eQPP Caco	fQP log BB	gQP log *K*_p_	hQP log *K*_hsa_	ihuman oral absorption	jpercent human oral absorption	krule of five
**12**	7.149	5.921	–6.542	–6.850	3884.747	–0.101	–0.127	1.110	1	100.000	1
**13**	7.313	6.375	–6.885	–6.536	3724.424	0.031	–0.234	1.411	1	100.000	1
**14**	8.471	4.697	–5.540	–6.415	2057.140	–0.237	–0.959	0.705	3	100.000	0
**15**	7.284	5.803	–6.592	–6.548	3777.563	–0.049	–0.424	1.118	1	100.000	1
**16**	10.845	5.380	–6.893	–7.683	836.226	–1.048	–1.151	0.824	1	100.000	1
**17**	14.629	3.413	–5.741	–6.712	173.444	–1.552	–2.983	0.494	3	87.006	0
**18**	8.179	4.995	–6.502	–6.589	451.337	–1.012	–2.227	1.046	1	100.000	0
**19**	7.786	6.033	–6.754	–6.262	4207.162	–0.132	–0.485	1.094	1	100.000	1
**20**	9.117	5.110	–6.150	–6.497	1585.726	–0.418	–1.070	0.989	3	100.000	1

a Predicted octanol/water distribution coefficient: −2.0–6.5.

b Predicted water/gas distribution coefficient: 4.0–45.0.

c Predicted aqueous solubility, log *S*. mol dm^–3^ in *S* is the concentration of solute in a saturated solution in equilibrium with the crystalline solid: −6.5–0.5.

d Estimated IC_50_ value for blockade of HERG K+ channels concern below −5.

e Estimated apparent Caco-2 cell permeability in nm/sec; Caco-2 cells are a model for the intestinal blood barrier; QikProp estimates are for inactive migration <25 poor, >500 very good.

f Predicted brain/blood distribution coefficient. Note: QikProp estimates are for oral medications, so dopamine and serotonin, for example, are CNS-negative because they are too polar to cross the very blood-brain barrier: −3.0–1.2.

g Projected skin permeability, log *K*p: −8.0 to −1.0.

h Prediction of binding to human serum albumin −1,5–1,5.

i Predicted qualitative human oral absorption: 1, 2, or 3 for low, medium, or high.

j Estimated human oral absorption on a scale of 0 to 100%. This property is generally well correlated with HumanOralAbsorption, as both measure the same property. >80% high, <25% weak.

k The number of violations of Lipinski’s rule of five. The guidelines are mol *M*_W_ < 500, QP log Po/w < 5, donorHB ≤ 5, acceptHB ≤ 10. Compounds meeting these guidelines are considered drug-like (“Five” means limits in multiples of 5) (maximum 4).

#### 3.4.2 Molecular Docking

**Figure 4:**
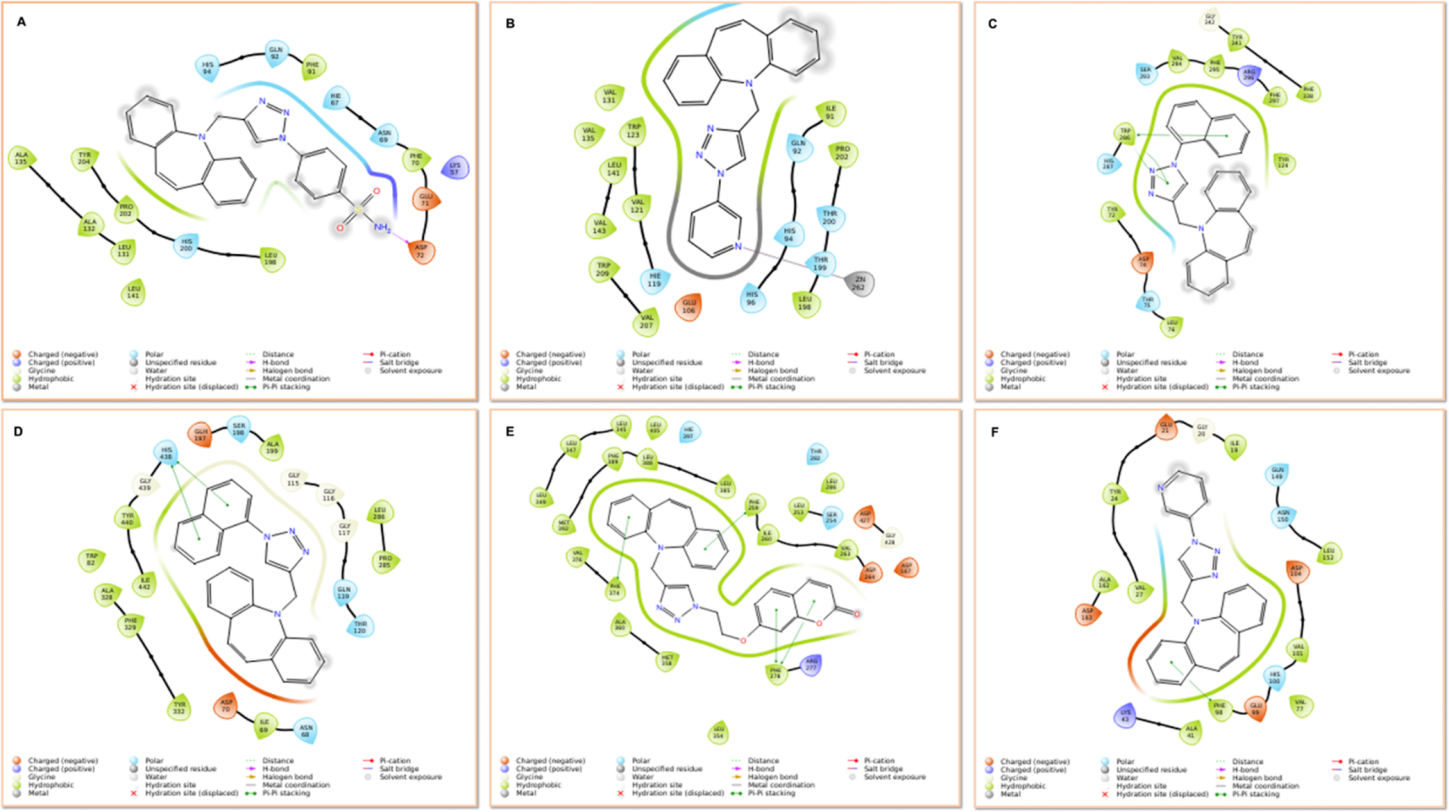
2D interaction diagram of potential enzyme inhibitors. (A) Compound **17**-hCA I. (B) Compound **14**-hCA II. (C) Compound **13**-hAChE. (D) Compound **13**-hBChE. (E) Compound **16**-SphK1. (F) Compound **14**-CDK6.

To predict the binding mode analysis of the synthesized compounds, a molecular docking study was performed by using the Glide/SP method. The two-dimensional (2D) ligand interactions are shown in Figure 4. For this purpose, all compounds were docked against hCA I, hCA II, hAChE, hBChE, and human kinases such as SphK1 and CDK6. The docking scores of compounds **13, 14**, and tacrine were calculated as −8.555, −8.644, and −8.554 kcal/mol, respectively. The hydrogen atom of the pyridine ring of compound **14** interacted with Asn69 in the binding pocket of hCA I. The pyridine nitrogen atom of compound **14** interacted with the zinc ion against hCA II. The sulfonamide oxygen and thiadiazol ring of the AZA known inhibitor exhibited H-bond and π–π interactions with Thr199 and His294, respectively. The docking scores of the *in vitro* effective compounds were calculated as near a known inhibitor of hCAs. For instance, compound **14** showed the binding ability with the docking scores of −4.424 kcal/mol for hCA I and −5.268 kcal/mol for hCA II, whereas the docking score was determined as −4.461 and −5.864 kcal/mol for AZA.

According to our calculations, the docking scores of the potent compounds (**16** and **13)** were determined by *in silico* analysis against human disease targets (AChE and BChE) as −7.148 and −8.555 kcal/mol, respectively, compared to the docking score of known inhibitor TAC. The H-bond and π–π interactions with the key residues including Phe295 and Trp286 at the active site of the AChE were observed between the carbonyl group of coumarin and phenyl rings of compound **16**, respectively. On the other hand, *in vitro* effective compound **13** exhibited the π–π interaction between Trp286 and phenyl and triazole rings. The π–π interaction between the naphthalene ring of compound **13** and His438 residue was observed in the active site of BChE, and also -NH group of TAC interacted with Trp82.

The most significant drug targets for cancer therapy were used to identify the inhibitor potency of the click products (**12**–**20**). The docking scores targeting SphK1 were calculated as −10.505–9.873, −9.512, and −9.079 kcal/mol for compounds **16, 12, 20**, and **14**, respectively. Among the molecular docking study, coumarin-substituted dibenzoazepine-triazole-linked hybrid compound exhibited high binding ability in this study. Cyclin-dependent kinases such as CDK6 regulate the cell cycle, and it is known that the development of effective and safe small-molecule CDK inhibitors as anticancer therapeutics has been difficult for many years. Thus, in this study, the compounds were evaluated *viain silico* studies against CDK6. The results indicate that compounds **14** and **20** have good docking scores of −8.031 and −8.022 kcal/mol, respectively.

#### 3.4.3 Molecular Dynamics (MD) Simulations and Molecular Mechanics Generalized Born Surface Area (MM/GBSA) Calculations

**Figure 5:**
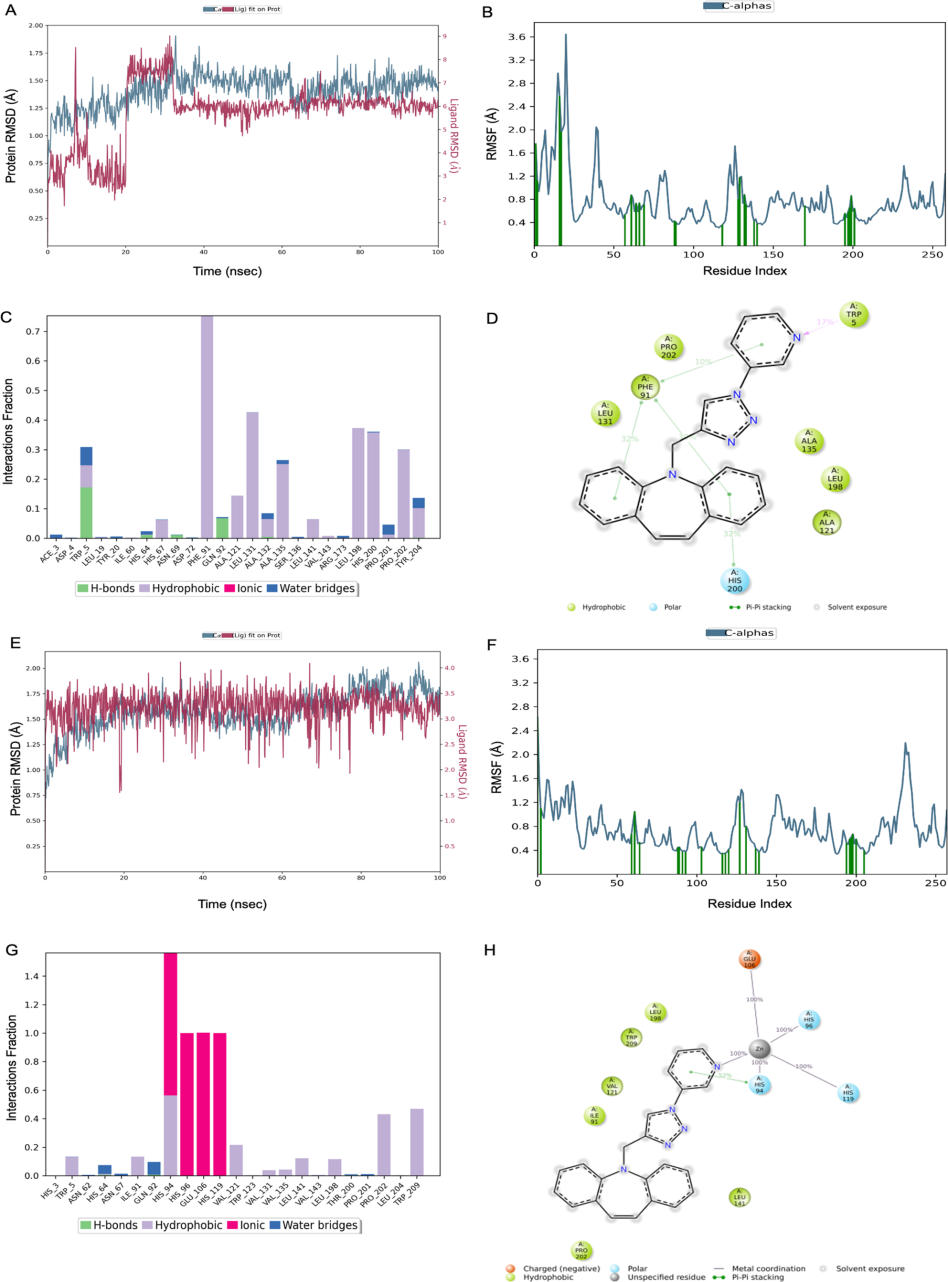
(A–D) Compound **14** hCA I (RMSD protein = 1.41 ± 0.15 Å, RMSD ligand = 5.67 ± 1.35 Å, Δ*G* = −47.15 ± 5.38 kcal/mol). (E–H) Compound **14** hCA II (RMSD protein = 1.57 ± 0.20 Å, RMSD ligand = 3.23 ± 0.33 Å, and Δ*G* = −33.33 ± 5.94 kcal/mol).

In this study, the 100 ns MD simulations of *in vitro* and *in silico* effective compounds (**12, 13, 14, 16, 17, 19, 20)** for each target were analyzed by using Desmond to investigate the interactions in detail. Then, MM/GBSA calculations were performed by each 10 ns trajectories of the compounds through the Prime module of Maestro. The MD simulation study of the compound **14**–2FW4 complex showed that the RMSD value of ligand-bound protein Cα was found as 1.41 ± 0.15 Å (Figure 5a). The RMSF values were obtained for most of the fluctuations below 2 Å (Figure 5b). The strong hydrophobic interaction was obtained with Phe91, Leu131, and Leu198 in the bar graph. The H-bonding interaction was observed with Trp5, His64, and Gln92. (Figure 5c). It was seen that the dibenzoazepine scaffold exhibited hydrophobic interactions with Phe and His residues (Figure 5d). The binding free energy was calculated as −47.15 ± 5.38 kcal/mol for compound **14** targeting hCA I. The MD simulation study of the compound **14**-1G45 complex showed that the RMSD value of ligand-bound protein Cα was found as 1.57 ± 0.20 Å (Figure 5e). The RMSF values were obtained for most of the fluctuations below 1.6 Å (Figure 5f). The strong ionic interactions with His94, His96, Glu106, and His119 residues were seen in the interaction bar diagram (Figure 5g,h). The binding free energy was calculated as −33.33 ± 5.94 kcal/mol for compound **14** targeting hCA II.

**Figure 6:**
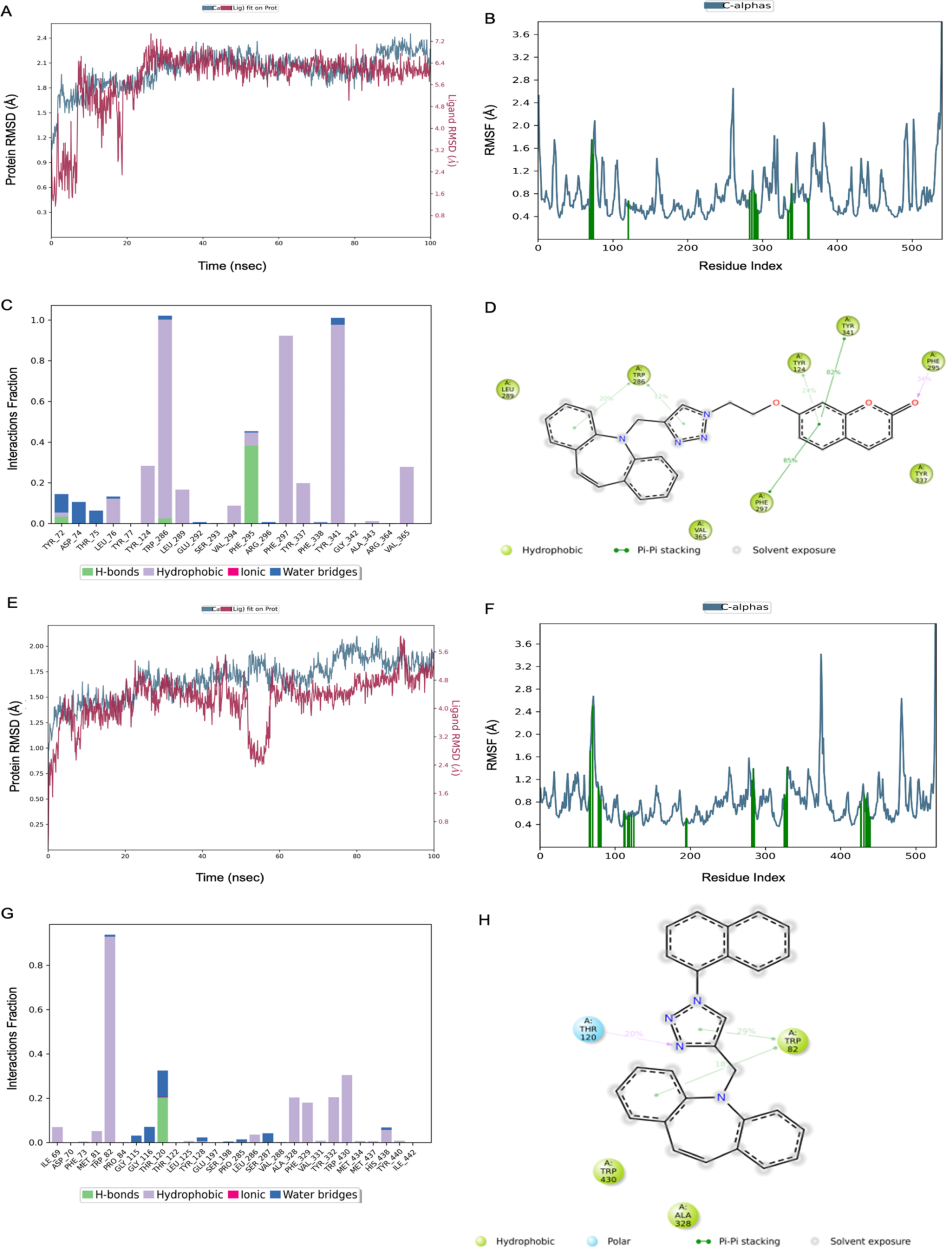
(A–D) Compound **16** hAChE (RMSD protein = 2.02 ± 0.22 Å, RMSD ligand = 5.81 ± 1.13 Å, and Δ*G* = −107.84 ± 4.58 kcal/mol). (E–H) Compound **13** hBChE (RMSD protein = 1.67 ± 0.20 Å, RMSD ligand = 4.26 ± 0.68 Å, and Δ*G* = −63.59 ± 4.59 kcal/mol).

The MD simulation study of the compound **16**–4EY7 complex showed the RMSD value of ligand-bound protein Cα as 2.02 ± 0.22 Å (Figure 6a). It was seen that the RMSF values were observed to be stable, with fluctuations of most of the residues approximately and below 2.2 Å (Figure 6b). The strong interactions with Trp286 and Phe295 contain H-bonds, hydrophobic, and water bridges. The most hydrophobic interactions were observed with Phe297, Try124, Tyr337, Leu289, Val365, and Val294 (Figure 6c). The π–π and hydrogen bonding interactions of the coumarin scaffold with Phe297, Tyr341, and Phe295 were retained for 85, 82, and 34% of the total simulation time, respectively (Figure 6d). The binding free energy was calculated as −107.84 ± 4.58 kcal/mol for compound **16** targeting AChE. The MD simulation study of the compound **13**–6I0C complex revealed that the RMSD value of ligand-bound protein Cα was found as 1.67 ± 0.20 Å (Figure 6e). The RMSF values were obtained for most of the fluctuations below 1.6 Å (Figure 6f). The strong hydrophobic interaction was obtained with Trp82 in bar graph. The H-bonding interaction was observed with Thr120 (Figure 6g). The H-binding of the nitrogen atom on the triazole ring with Trp82 was retained for 20% of the total trajectory. The π–π stacking between the triazole and dibenzoazepine rings and Trp82 were observed (Figure 6h). The binding free energy was calculated as −63.59 ± 4.59 kcal/mol for compound **13** targeting BChE.

**Figure 7:**
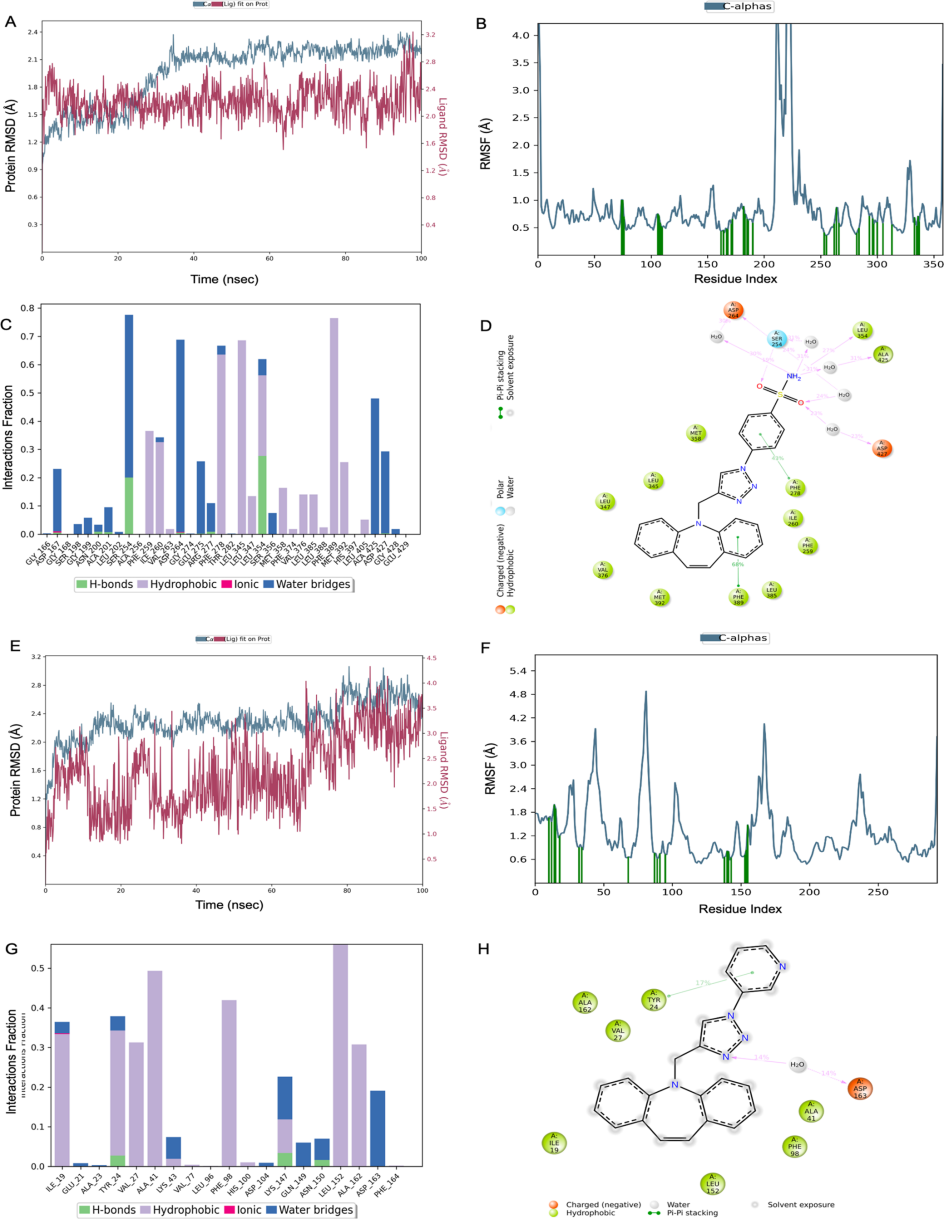
(A–D) Compound **17** SphK1 (RMSD protein = 1.96 ± 0.34 Å, RMSD ligand = 2.22 ± 0.24 Å, and Δ*G* = −78.82 ± 5.82 kcal/mol). (E–H) Compound **14** CDK6 (RMSD protein = 2.30 ± 0.27 Å, RMSD ligand = 2.22 ± 0.74 Å, and Δ*G* = −59.12 ± 4.91 kcal/mol).

The MD simulation study of the compound **17**-4V24 complex showed that the RMSD value of ligand-bound protein Cα was found as 1.96 ± 0.34 Å (Figure 7a). The RMSF values were obtained for most of the fluctuations below 2 Å (Figure 7b). The strong hydrophobic interaction was obtained with Phe278 and Phe389 in the interaction bar graph. The H-bonding interaction was observed with Trp5, His64, and Gln92 (Figure 7c). It was seen that the dibenzoazepine scaffold exhibited hydrophobic interactions with the Phe residue (Figure 7d). The binding free energy was calculated as −78.82 ± 5.82 kcal/mol for compound **14**. The MD simulation study of the compound **14**-1G45 complex showed that the RMSD value of ligand-bound protein Cα was found as 2.30 ± 0.27 Å (Figure 7e). The RMSF values were obtained for most of the fluctuations below 2.4 Å (Figure 7f). The H-bonds with Lys, Gln, Asn, and Asp residues are seen in the interaction bar diagram (Figure 7g,h). The binding free energy was calculated as −59.12 ± 4.91 kcal/mol for compound **14**.

According to MD simulations, the compound **12**-4 V24 complex showed that the RMSD value of ligand-bound protein Cα was found as 2.12 ± 0.27 Å (Figure S29a, in Supporting Information, SI). The RMSF values were obtained for most of the fluctuations below 2 Å (Figure S29b, SI). The strong hydrophobic interaction was obtained with Phe278 and Leu345 in the interaction bar graph. The H-bonding was observed with Thr282 (Figure S29c, SI). It was seen that the dibenzoazepine scaffold exhibited hydrophobic interactions with Phe278 and Phe389 (Figure S29d, SI). The Prime MM/GBSA calculations were carried out following the 100 ns MD simulations. The binding free energy was calculated as −78.23 ± 5.37 kcal/mol for compound **12**. The MD simulation of the compound **16**-5L2I complex showed that the RMSD value of ligand-bound protein Cα was found as 1.79 ± 0.23 Å (Figure S29e, SI). The RMSF values were obtained for most of the fluctuations below 3.5 Å (Figure S29f, SI). H-bonds with Asp264 were seen in the interaction diagram (Figure S29g,h SI). The binding free energy was calculated as −91.81 ± 3.86 kcal/mol for compound **16**.

The compound **19**-4V24 complex showed that the RMSD value of ligand-bound protein Cα was found as 1.94 ± 0.22 Å (Figure S30a, SI). The RMSF values were obtained for most of the fluctuations below 2 Å (Figure S30b, SI). The strong hydrophobic interaction was obtained with Phe278, Leu345, and Phe389 in the bar graph (Figure S30c, SI). It was seen that methoxy substituents contributed to the hydrophobic interactions of Asp167 and Leu354 with water bridges (Figure S30d, SI). The binding free energy was calculated as −88.10 ± 5.37 kcal/mol for compound **19**. The MD simulation study of the compound **20**-5L2I complex showed that the RMSD value of ligand-bound protein Cα was found as 2.94 ± 0.35 Å (Figure S30e, SI). The RMSF values were obtained for most of the fluctuations below 2.5 Å (Figure S30f, SI). H-bonds with Lys147 and Asn150 were seen in the interaction diagram (Figure S30g,h SI). The binding free energy was calculated as −77.06 ± 2.69 kcal/mol for compound **20**.

## 4 Discussion

We previously reported that novel substituted triazole-linked tetrafluoronaphthalene hybrid derivatives as click products were effective anticancer agents for breast cancer.^44^ Two of the compounds bearing 4-methoxy and sulfonamide groups exhibited cell cytotoxicity at 20 μM and 30 μM concentrations in TNBC BT-549 cells, and also, one of the other compounds blocked the migration of MDA-MB-231 cells.^44^ Herein, among dibenzoazepine-substituted triazole hybrids, pyridine, coumarin, sulfonamide, and trimethoxybenzene moieties contributed to cell cytotoxicity on BT-549 cells under 20 μM. In one of our previously reported studies, *in silico* ADME prediction was performed by QikProp for the synthesized triazole-linked tetrafluoronaphthalene hybrid compounds.^44^ Similarly, this study includes *in silico* prediction for newly synthesized triazole-linked dibenzoazepine hybrid compounds. Our findings revealed that although the most functional groups are the same, the triazole-linked substituted tetrafluoronaphthalene and dibenzoazepine click products had different toxicity properties due to the structural differences. For instance, triazole-linked dibenzoazepine compounds containing sulfonamide and nitro group were determined with below 500 Caco-2 cell permeability values compared to our previous study.^44^

AZA was reported as a standard inhibitor of CA that interacts zinc ion, His, Asn, and Thr residues in the active sites of hCAs.^60^ According to the recently reported studies, the docking scores were calculated as 6.6 kcal/mol and −5.25 kcal/mol for known inhibitor AZA against hCA II (3HS4) and hCA I (1AZM) isoenzymes, respectively.^61,62^ Synthesized various sulfonamides were determined as potential candidates to inhibit of hCAs.^62−64^ Triazole-ring-incorporated benzenesulfonamide compounds were reported as hCAs inhibitors with H-bond and π–π stacking interactions. It is understood that the triazole ring contributes to the biological activity. As seen in Table 1, compound **17** having benzenesulfonamide group inhibited hCA isoforms. It is mentioned that the sulfonamide groups bind to active site through zinc ion.^65^ As expected, compound **14** binds to zinc ion by a salt bridge in this study. Overall, most of the compounds showed good and moderate binding scores to targeted hCAs compared to the AZA standard.

It is mentioned that AChE and BChE enzyme inhibition is a therapeutic approach to be used in the treatment of AD.^66,67^ It is seen that coumarin ring increases the inhibitor activity against AChE.^68^ Based on the literature findings, coumarins are the effective scaffolds to be used in the treatment of cholinesterases.^69,70^ We reported that substituted coumarin carboxamides showed strong inhibition against AChE and BChE.^70^ TAC is known as a potent inhibitor of both enzymes.^71,72^ As reported in the literature, the binding energies of TAC were reported as −7.926, −8.01, and −7.007 kcal/mol for AChE and BChE, respectively.^73,74^ It is indicated that the tacrine-coumarin hybrids have been used for multitarget drug design strategy.^67,75^ 1,2,3-Triazole-linked coumarin-containing TAC compounds were reported as cholinesterase inhibitors.^76^ The H-bonding interactions between coumarin and Trp279, Tyr121, Tyr70, and Trp286 active site residues,^75,76^ and also anion-pi interaction between the triazole ring and Trp279 were determined in the active site of AChE.^76^ Among the reported tacrine conjugates, the molecular docking study showed that H-bonding was observed between the nitrogen atom and Trp86 against AChE.^71^ The substituted compounds containing triazole ring and coumarin were reported for their inhibitor activities targeting hCAs, AChE, and BChE, in which active site residues were identified.^60^ Pi-cation interaction was observed for coumarin and phenyl rings on the click products, and triazole ring contributed to the interaction with active site residues of hCA I, hCA II, AChE, and BChE.^60^ Similarly, newly synthesized triazole-linked click products have been reported with good docking scores as −10.4 and −9.4 kcal/mol for AChE inhibition, and also they have been indicated as promising dual AChE/Aβ aggregation inhibitors to design new anti-Alzheimer agents.^77^ In one of the reported studies, it was indicated that carbonyl oxygen on coumarin ring interacted with the –NH group of Phe295 in the active site of AChE.^78^ Active site residues such as His438 and Trp82 were reported for BChE.^61,79^

We previously reported triazole-linked naphthalene hybrids as potential SphK1 inhibitor candidates that is highly upregulated in TNBC cells, *viain silico* analysis.^44^ According to our reported findings, *in vitro* effective compounds **12**–**14** and **18** showed good binding ability with key residues in the active site of SphK1.^44^ On the contrary, in this study, it is seen that the top docking score was found for coumarin-substituted triazole-linked hybrid compound **16**.

Throughout the 100 ns MD simulations of the potent triazole-linked naphthalene click products, the protein RMSD values were calculated between 1.0 and 3.1 Å for five ligand-SphK1 complexes compared to known SphK1 inhibitor PF543.^44^*In vitro* anticancer activity and *in silico* results showed that pyridine-, methoxy-, sulfonamide-, and thiazole-containing triazole-linked click products were determined as *in silico* effective functional groups.^44^

### 4.1 Structure–Activity Relationship (SAR)

Among the designed and synthesized compounds, most compounds exhibited strong biological activity. Although thiadiazol-containing benzamide compound AZA has a sulfonamide group, dibenzodiazepine- and pyridine-substituted triazole hybrid compound **14** has been found 4.16-fold more active than AZA. During the theoretical studies, the docking score of the most effective AChE and BChE inhibitor compound **14** was higher than that of TAC. Compound **14** interacted with Phe91, Trp5, and His200 for hCA I, and pyridine ring directly interacted with the active site Zn^2+^ ion for hCA II. MM/GBSA was calculated as −47.15 ± 5.38 and −33.33 ± 5.94 kcal/mol for compound **14** targeting hCA I and hCA II, respectively. It was supported by cancer cell culture studies, and therefore, compound **14** showed the most cytotoxic activity in the TNBC BT-549 cell line with the IC_50_ value of 16.59 ± 0.97 μM, and also it suppressed the colonies of BT-549 cells at 10 μM. In addition, this compound exhibited high binding ability (−9.079 kcal/mol) against cancer drug target SphK1. In the previous studies, SphK1 inhibitors such as PF543 and SKI-II were reported with their docking scores as 9.1 kcal/mol and −8.8 kcal/mol, respectively.^80^

However, in our previously reported study, we found the docking score of synthesized tetrafluoronaphthalene- and benzensulfonamide-substituted triazole-linked hybrid compound (−9.956 kcal/mol).^44^ It was supported in this study and our previous study that the pyridine ring contributed to the inhibition of colony formation in BT-549 cells. It was observed that dibenzoazepine- and pyridine-substituted triazole-linked hybrid compound **14** was the most effective for the inhibition of colonies compared to previously synthesized tetrafluoronaphthalene- and pyridine-substituted triazole-linked hybrid compound.^44^ Therefore, it has been shown here that the dibenzoazepine scaffold also increases the anticancer activity of the target compounds.

During MD simulations, the top docking pose of coumarin-substituted compound **16** interacted with the reported key residues including Trp286, Phe297, Tyr341, Tyr124, Phe295, and Phe297 in the active site of AChE (Figure 6d).^81^ The binding energy was calculated as −107.84 ± 4.58 kcal/mol. We also previously reported that coumarin contributed to AChE inhibition activity in *in silico* and *in vitro* studies.^70^ Triazole and benzyl moieties of the most effective compound **13** (IC_50_ = 1.15 ± 0.15 nM for BChE) interacted with Thr120 and Trp82. In addition, a strong correlation was obtained between the benzenesulfonamide for compound **17** from this study and compound **14** from our previously reported study due to the increased docking score against SphK1.^44^ As a result, it is thought that the benzenesulfonamide group is important to design potential inhibitor candidates for targeting SphK1. In addition, the reported studies suggested sulfonamide moiety as potential CA inhibitors.^82,83^

Compounds **14**, **16**, **17**, and **19** were determined as highly cytotoxic up to 20 μM in TNBC BT-549 cells. Compound **12** showed the highest cytotoxic activity at 12.51 ± 1.92 μM in TNBC MDA-MB-231 cells; however, other compounds had no cytotoxicity up to 100 μM except compound **14**. Overall, benzyl, pyridinyl, 7-ethoxy-coumaroyl, *p*-SO_2_NH_2_-phenyl, and 3,4,5-trimethoxyphenyl functionalities showed higher anticancer activity in TNBC cells.

**Figure 8:**
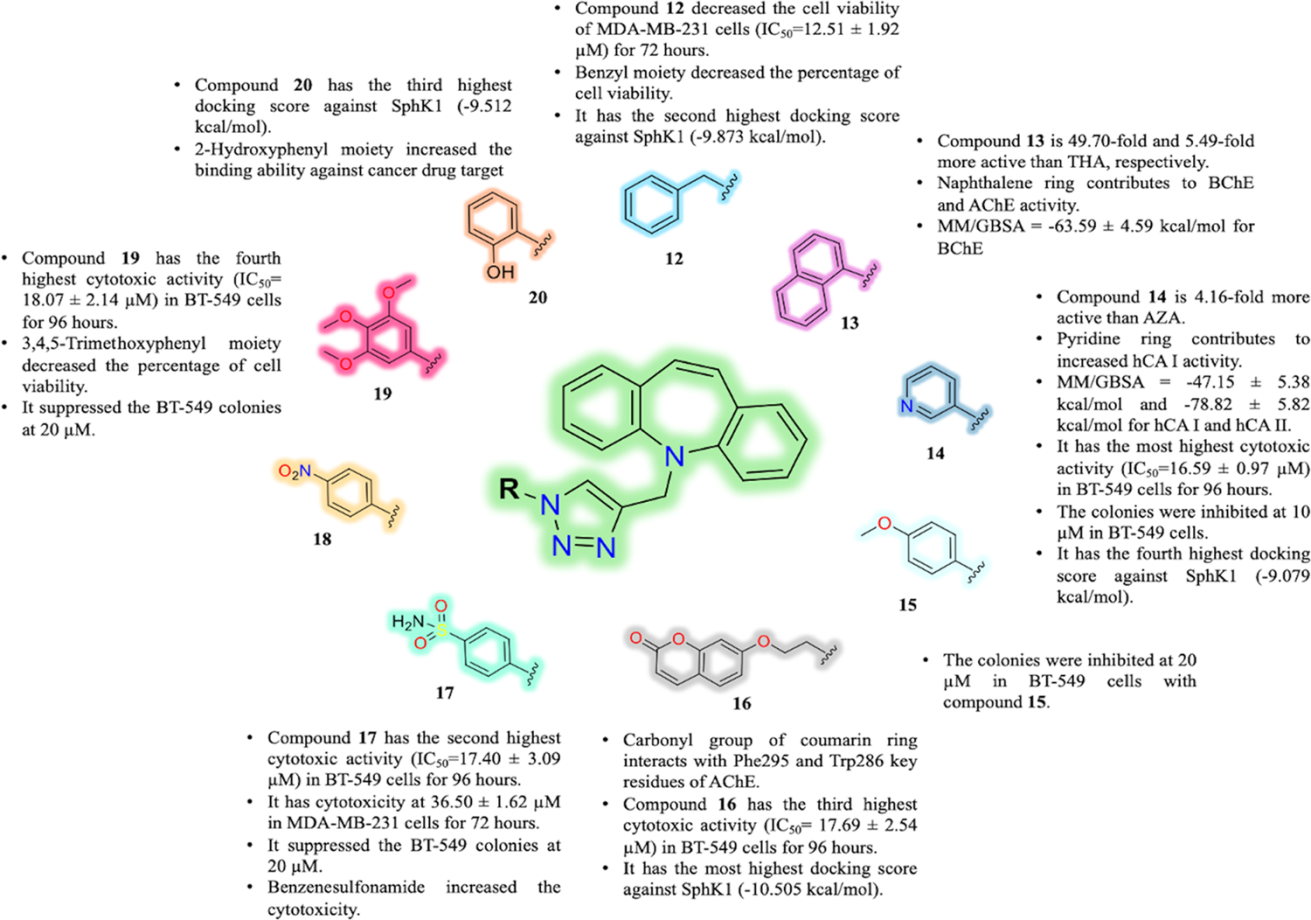
SAR study for this study.

Consequently, herein, we summarize the inhibition potentials of the synthesized novel hybrid compounds against CAs and ChEs, and also their anticancer activities in TNBC cells. Therefore, this SAR study will contribute to discovering more potent inhibitor candidates for further studies (Figure 8).

## 5 Conclusions

This study describes the design and synthesis of a novel series of dibenzoazepine-triazole hybrids for the development of bioactive and pharmacologically active compounds for potential use as anticancer agents and enzyme inhibitors. Compound **14** was 4.16-fold more active than acetazolamide (standard drug) for hCA I and 5.79-fold for hCA II. Compound **13** was 49.70-fold more active than THA (standard drug) for BChE and 5.49-fold for AChE

All of the compounds were tested for their *in vitro* cytotoxic effects on TNBC cells. The findings revealed that most compounds had good inhibition profiles during cell viability and colony formation and inhibition assays. According to this, compounds (**12, 14–17, 19, 20)** may be proposed as potent anticancer therapeutics for further *in vitro* investigations. Moreover, *in silico* techniques were used to determine the binding pattern of the compounds in the active site of the proteins, including hCAs and hChEs, and the selected cancer drug targets such as SphK1 and CDK6. Throughout the MD simulations, the compounds (**13** and **16)** for AChE and BChE, respectively, and compound **14** for hCA isoenzymes interacted with the key residues. Additionally, due to the *in vitro* cytotoxic effects, high docking scores of compounds **12, 14, 17, 19**, and **20** were evaluated by MD simulation analysis. It was seen that H-bonds and π–π interactions were observed with crucial residues that indicate the significance of the binding pattern of the targets. Overall, this study shows significant findings for the discovery and development of new enzyme inhibitors and anticancer agents.

## Supporting Information Available

^1^H NMR and ^13^C NMR (Figures S1–S10); HRMS and LC MS/MS (Figures S11–S19); and FTIR spectra (Figures S20–S28) of the synthesized compounds; *in silico* studies of the compounds **12, 16, 19, 20** (Figures S29 and S30) (PDF)
