# Supplementary material for: Novel Dibenzoazepine-Substituted Triazole Hybrids as Cholinesterase and Carbonic Anhydrase Inhibitors and Anticancer Agents: Synthesis, Characterization, Biological Evaluation, and In Silico Studies
Source: ACS Omega. 2024 Nov 16;9(47):46860–78. doi: 10.1021/acsomega.4c05804 (PMC11603219; doi:10.1021/acsomega.4c05804)
Supplement: Supplementary file 1 — ao4c05804_si_001.pdf [file ao4c05804_si_001.pdf]

Supporting Information

Novel Dibenzoazepine-Substituted Triazole Hybrids as Cholinesterase and Carbonic Anhydrase Inhibitors and Anticancer Agents: Synthesis, Characterization, Biological Evaluation and *In Silico* Studies

Musa Erdoğan[1,*], Alper Onder[2], Yeliz Demir[3], Ferah Comert Onder[4,*]

[1]*Department of Food Engineering, Faculty of Engineering and Architecture, Kafkas University, Kars, Türkiye*
[2]*Natural Products and Drug Research Laboratory, Department of Chemistry, Faculty of Science, Çanakkale Onsekiz Mart University, Çanakkale, Türkiye*
[3]*Ardahan University, Nihat Delibalta Gole Vocational High School, Department of Pharmacy Services, 75700 Ardahan, Türkiye*
[4]*Department of Medical Biology, Faculty of Medicine, Çanakkale Onsekiz Mart University, Çanakkale, Türkiye*

***Correspondence to**

Musa Erdoğan, Department of Food Engineering, Faculty of Engineering and Architecture, Kafkas University, 36100 Kars, Türkiye. Email: musaerdogan0@gmail.com

Ferah Comert Onder, Department of Medical Biology, Faculty of Medicine, Çanakkale Onsekiz Mart University, 17020 Çanakkale, Türkiye. Email: ferahcomertonder@comu.edu.tr

Table of contents

^{1}H NMR and ^{13}C NMR spectra…………………………………………..................2-11.

HRMS or LC MS/MS spectra ………………………………………………………12-15.

FTIR spectra.….....……………………………………………………………….16-20.

In silico studies of the compounds **12, 16, 19, 20**……………………………...……21-22.

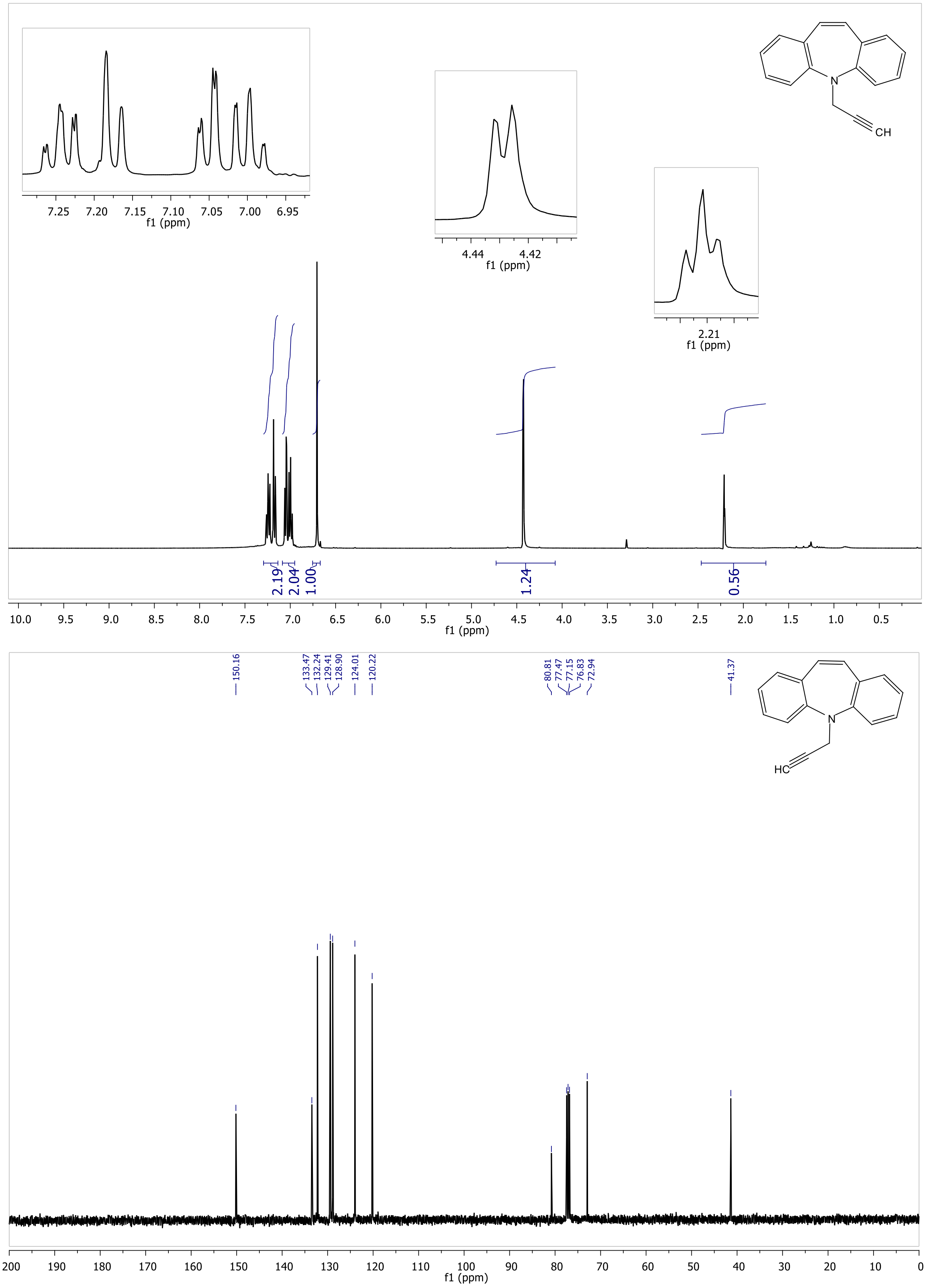

Fig. S1. ^{1}H-NMR (400 MHz, $CDCl_3$) and ^{13}C-NMR (100 MHz, $CDCl_3$) spectrum of **2.**

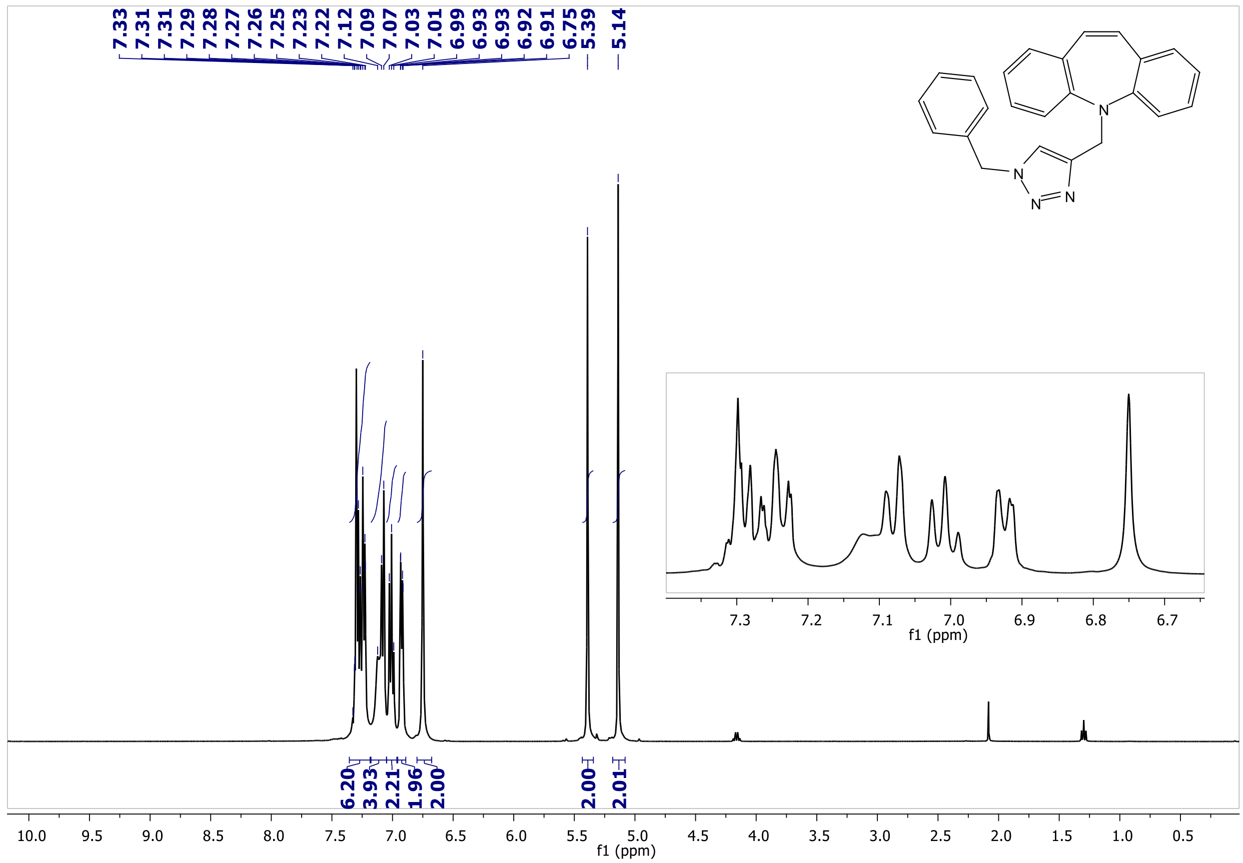

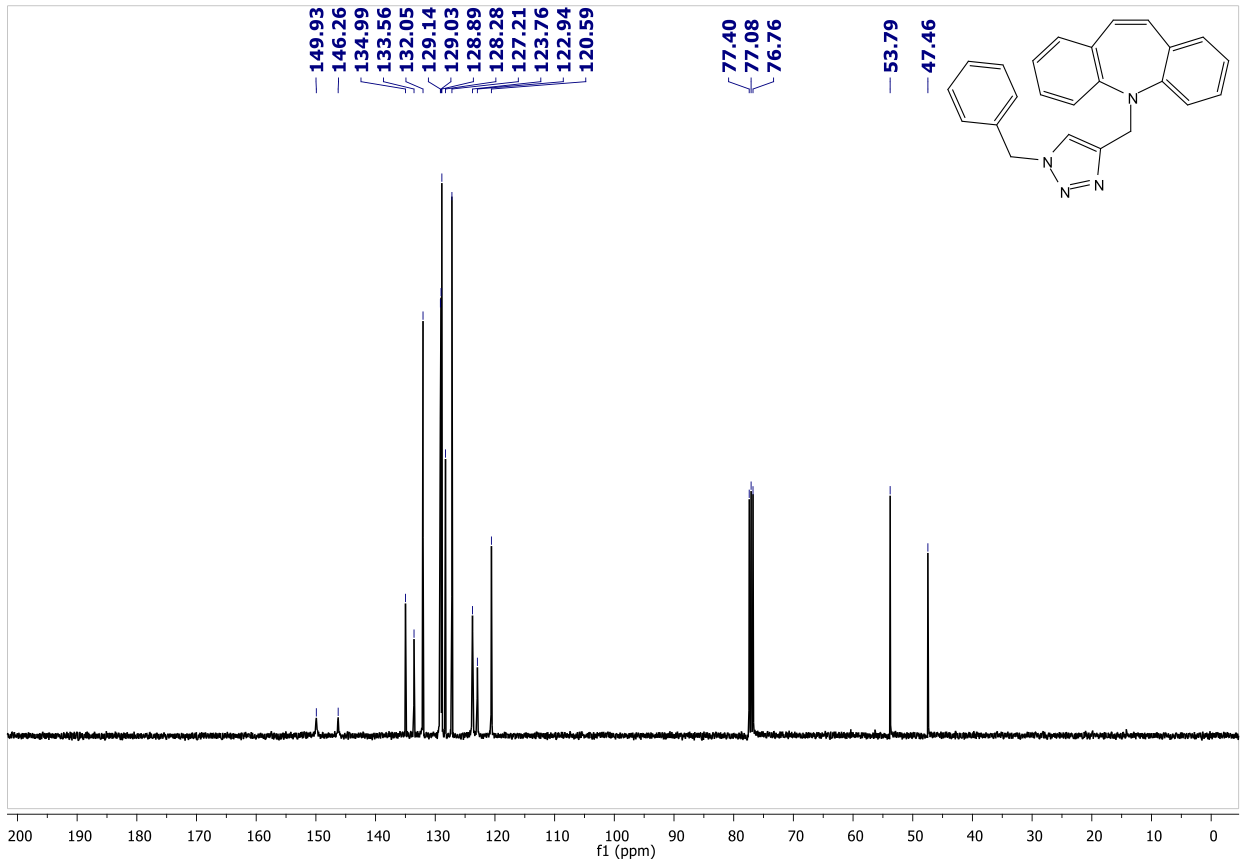

Fig. S2. ^{1}H-NMR (400 MHz, $CDCl_3$) and ^{13}C-NMR (100 MHz, $CDCl_3$) spectrum of **12.**

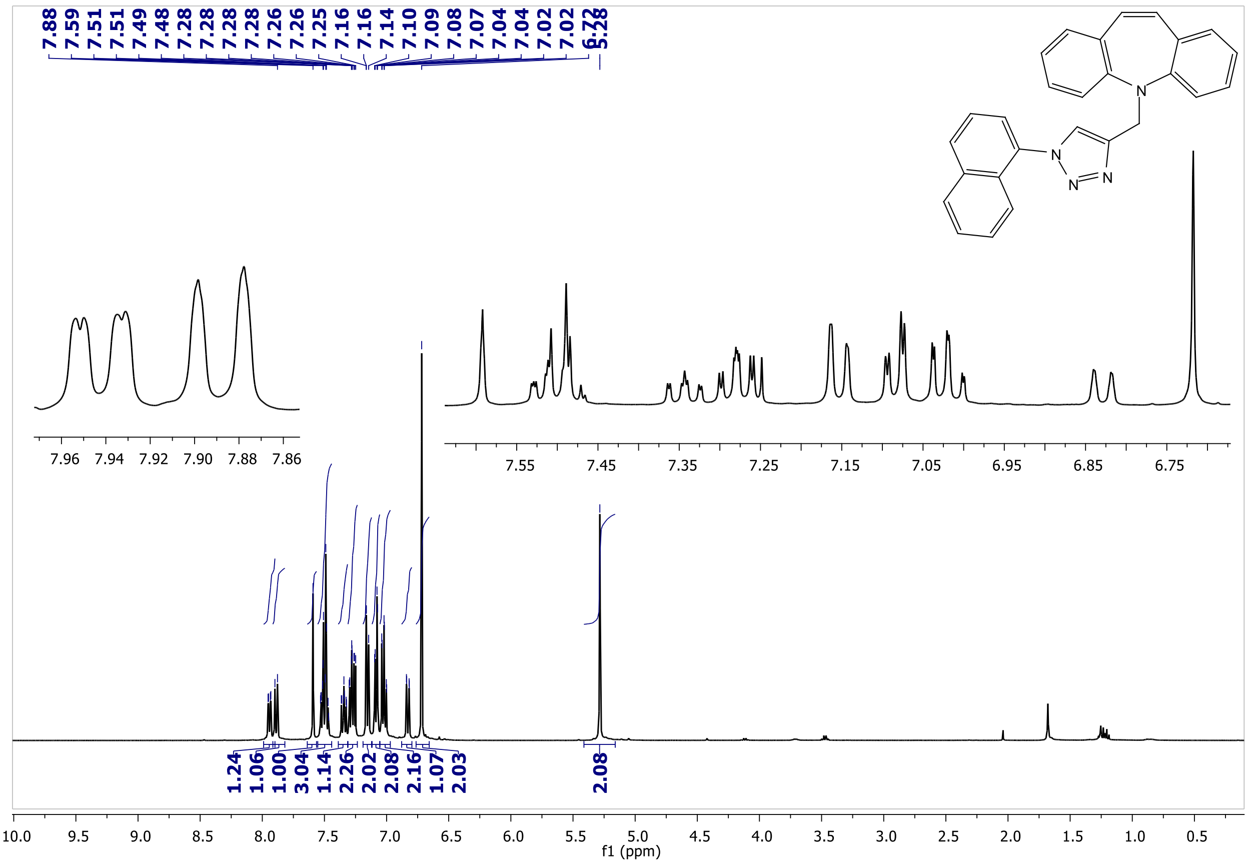

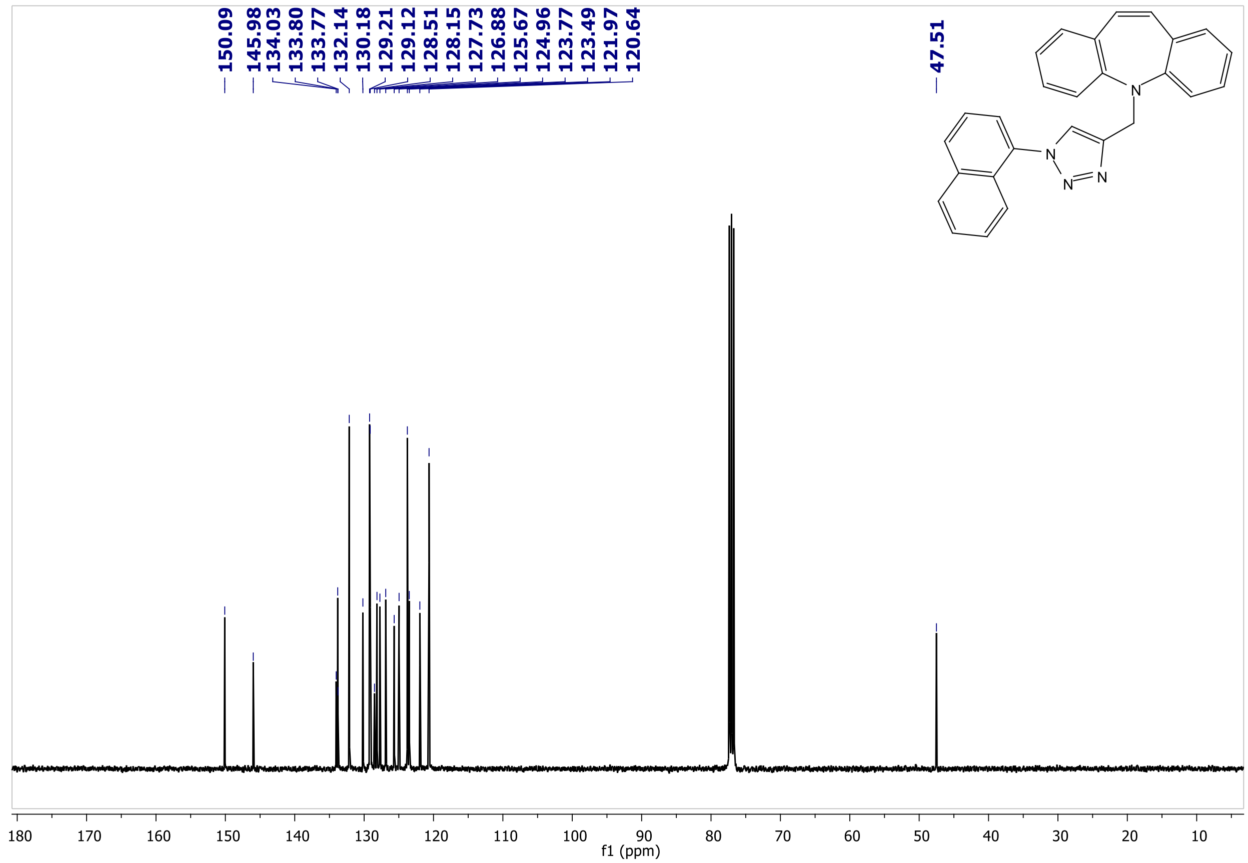

Fig. S3. ^{1}H-NMR (400 MHz, $CDCl_3$) and ^{13}C-NMR (100 MHz, $CDCl_3$) spectrum of **13.**

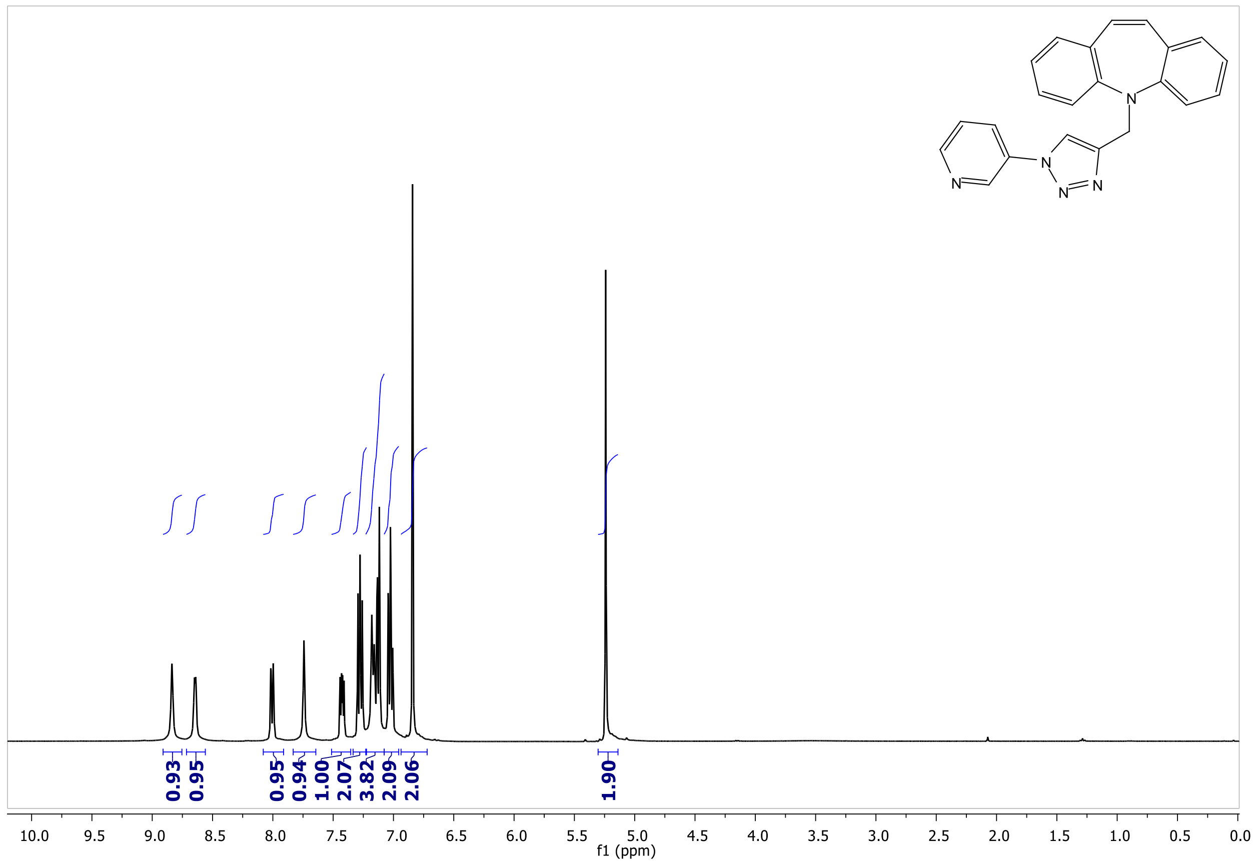

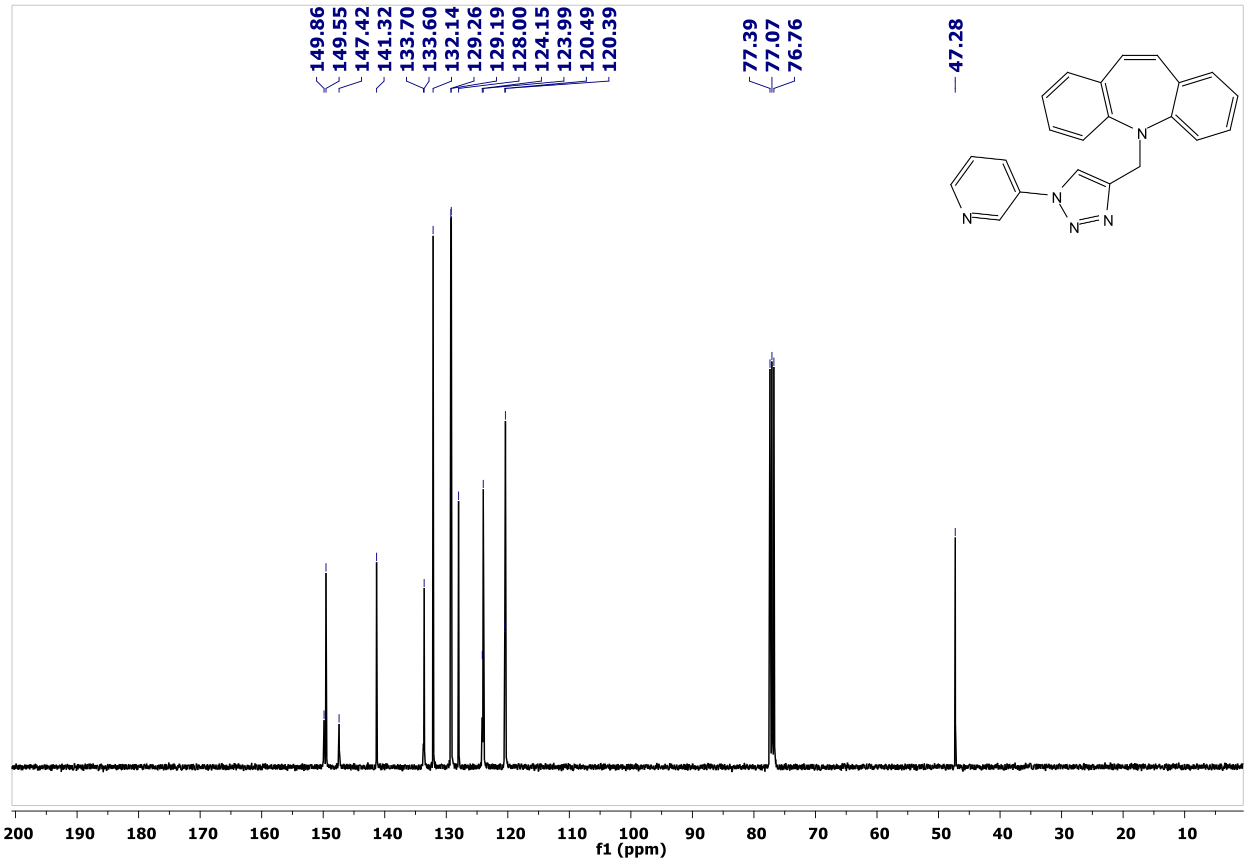

Fig. S4. ^{1}H-NMR (400 MHz, $CDCl_3$) and ^{13}C-NMR (100 MHz, $CDCl_3$) spectrum of **14.**

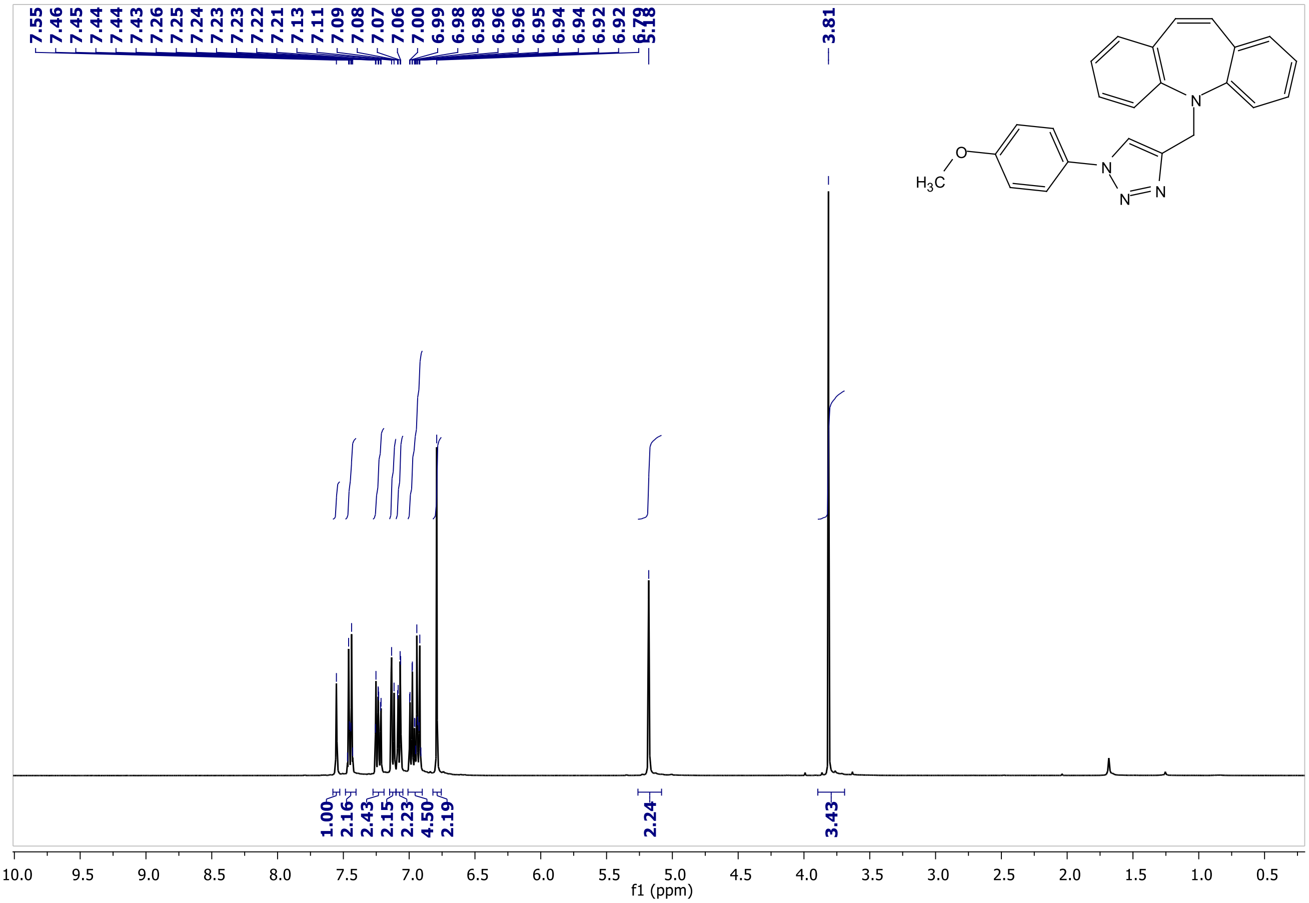

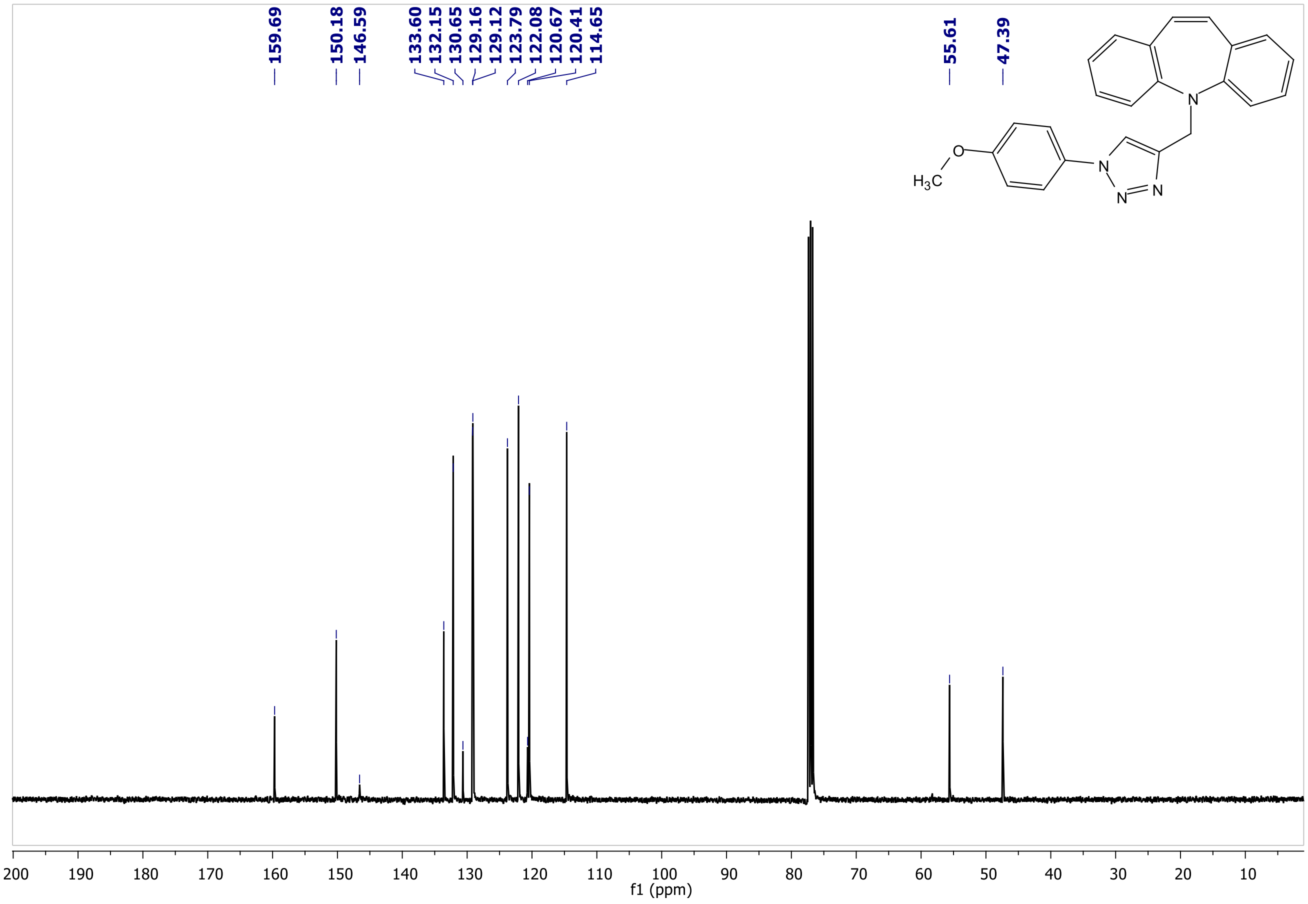

Fig. S5. ^{1}H-NMR (400 MHz, $CDCl_3$) and ^{13}C-NMR (100 MHz, $CDCl_3$) spectrum of **15.**

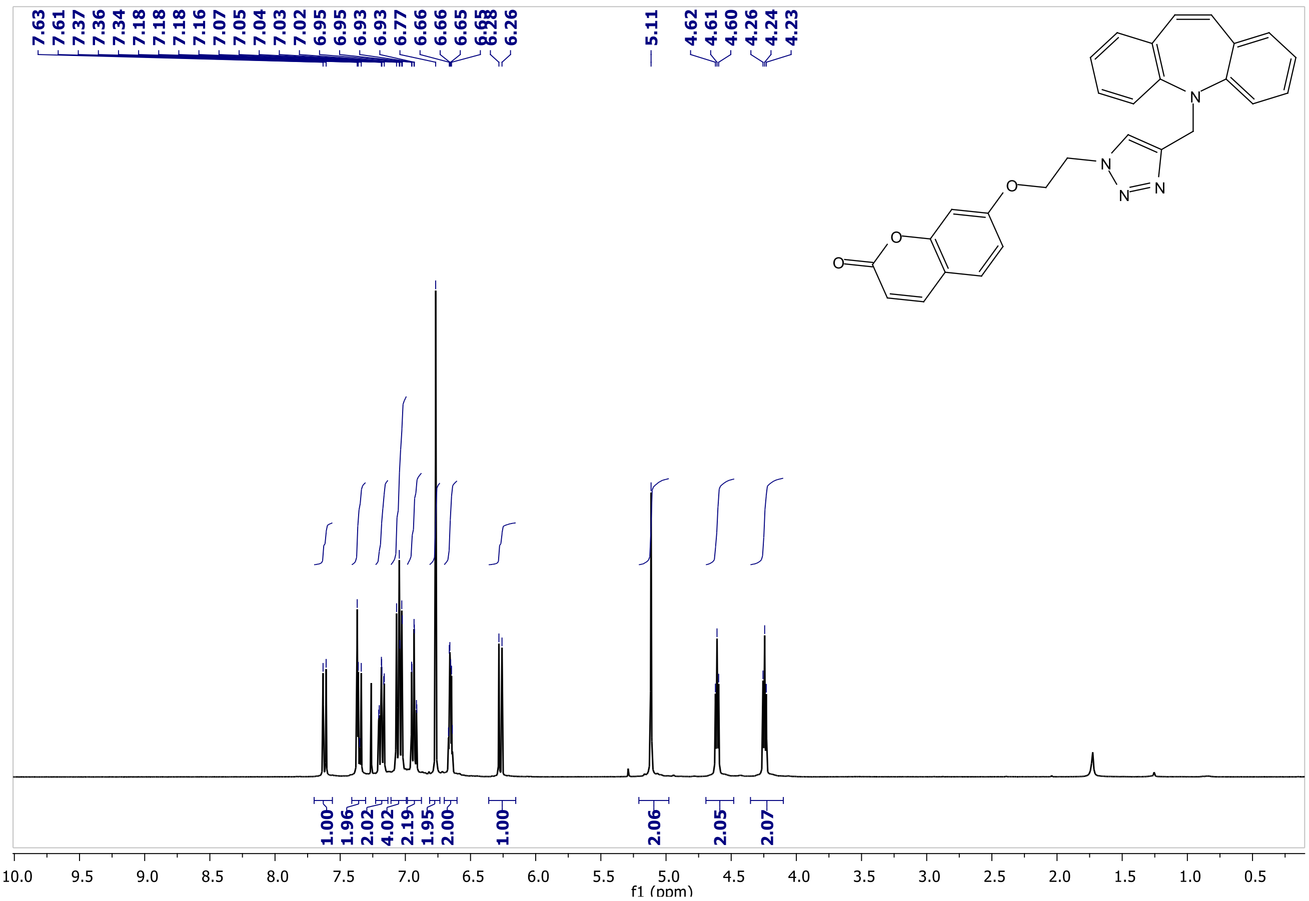

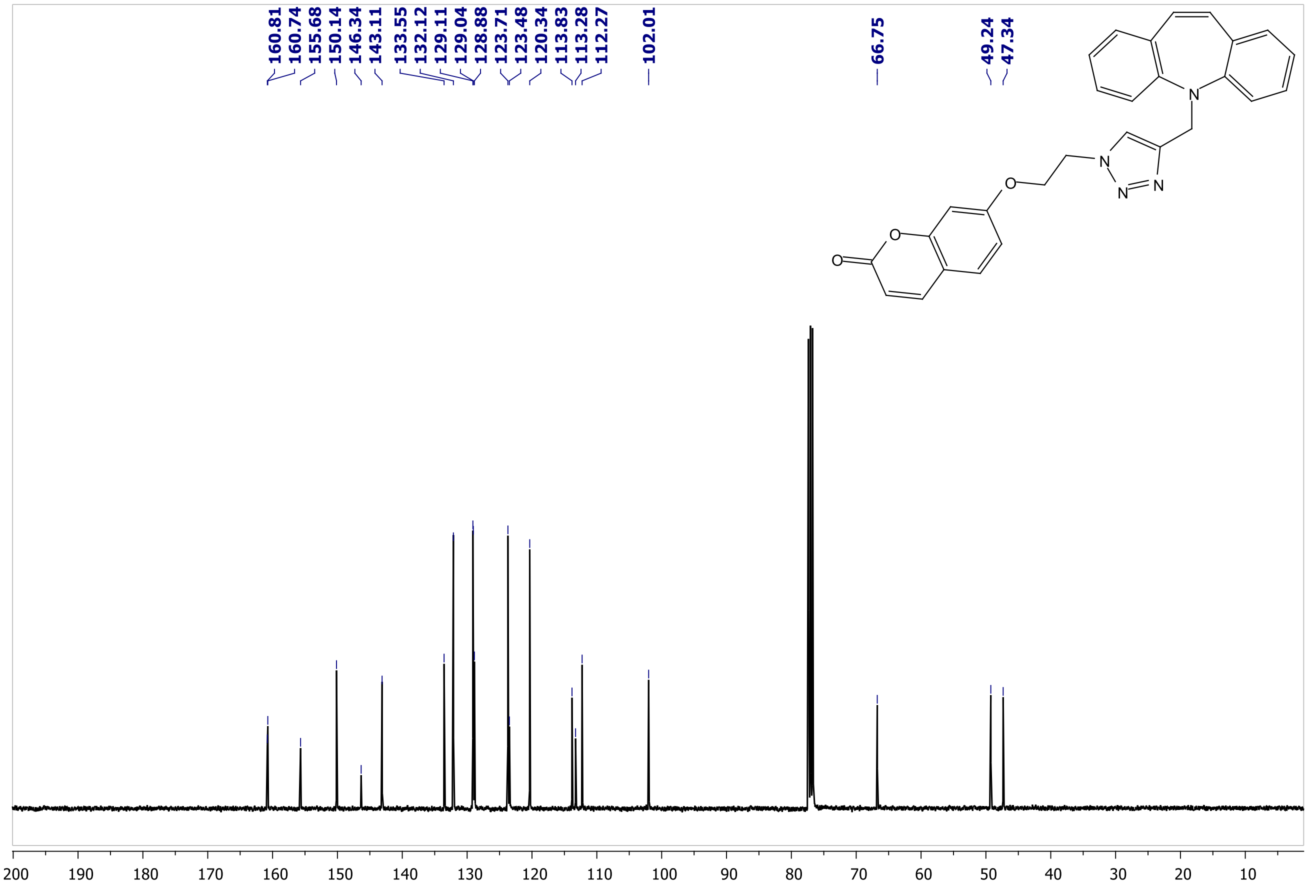

Fig. S6. ^{1}H-NMR (400 MHz, $CDCl_3$) and ^{13}C-NMR (100 MHz, $CDCl_3$) spectrum of **16.**

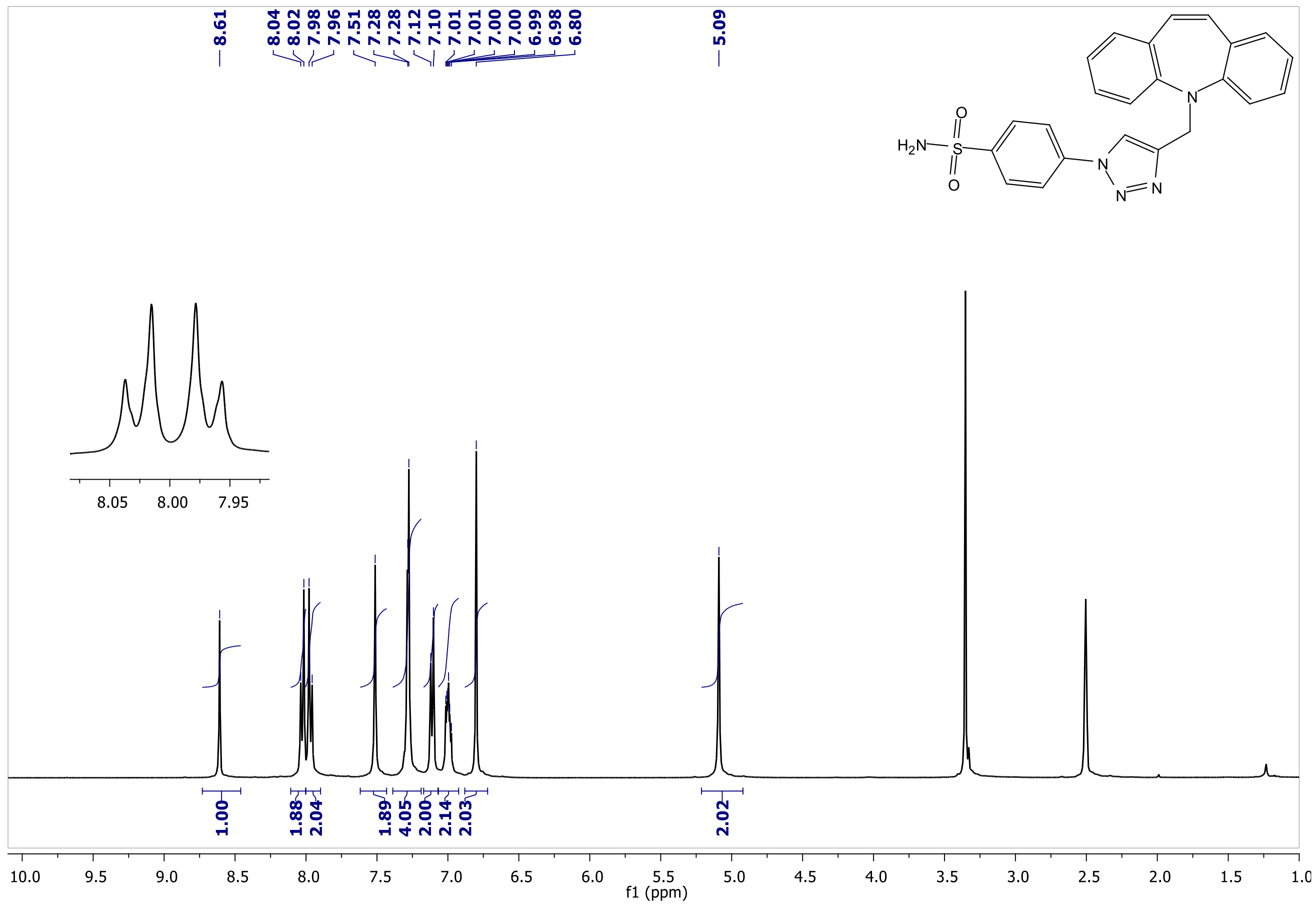

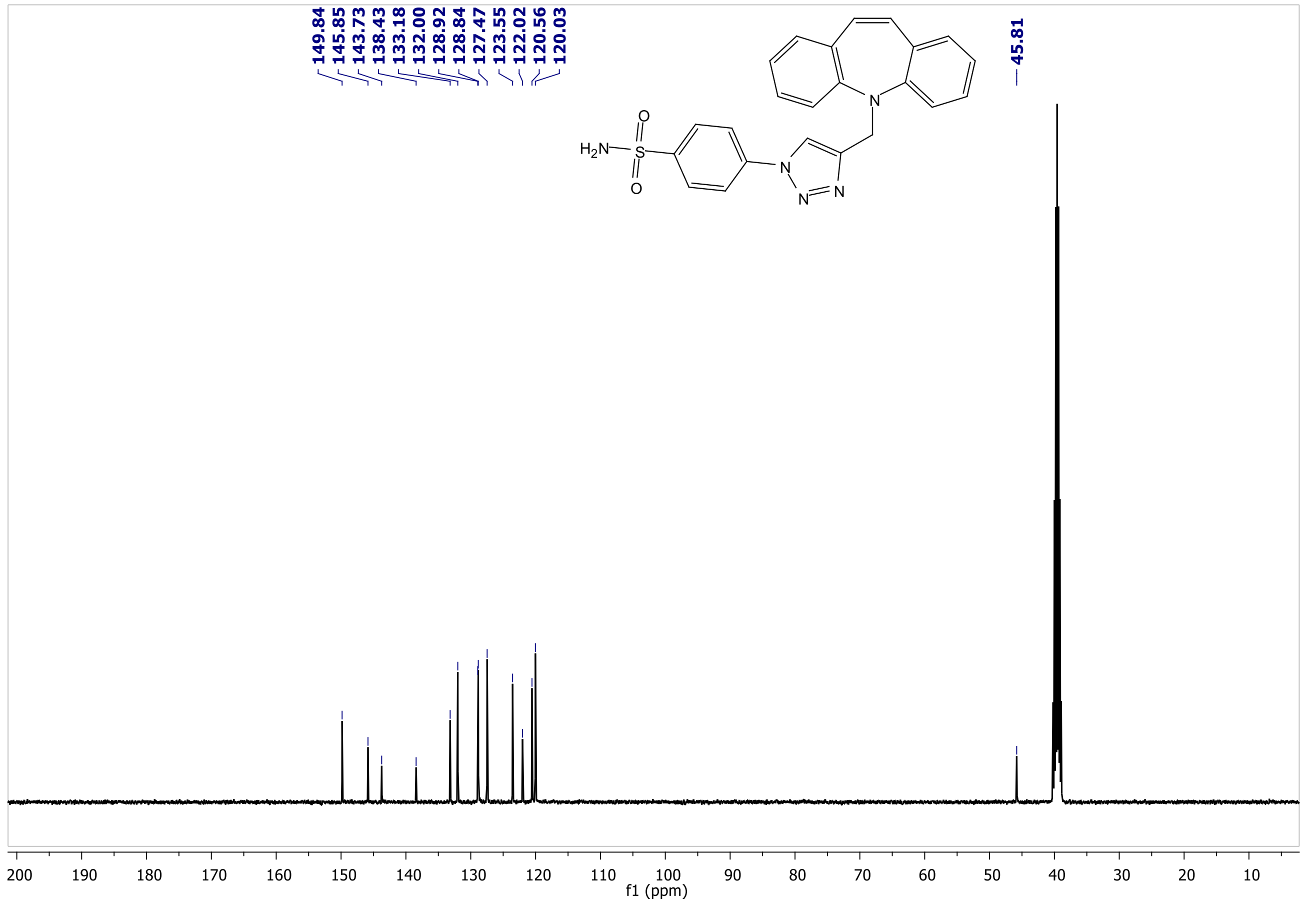

Fig. S7. ^{1}H-NMR (400 MHz, $CDCl_3$) and ^{13}C-NMR (100 MHz, $CDCl_3$) spectrum of **17.**

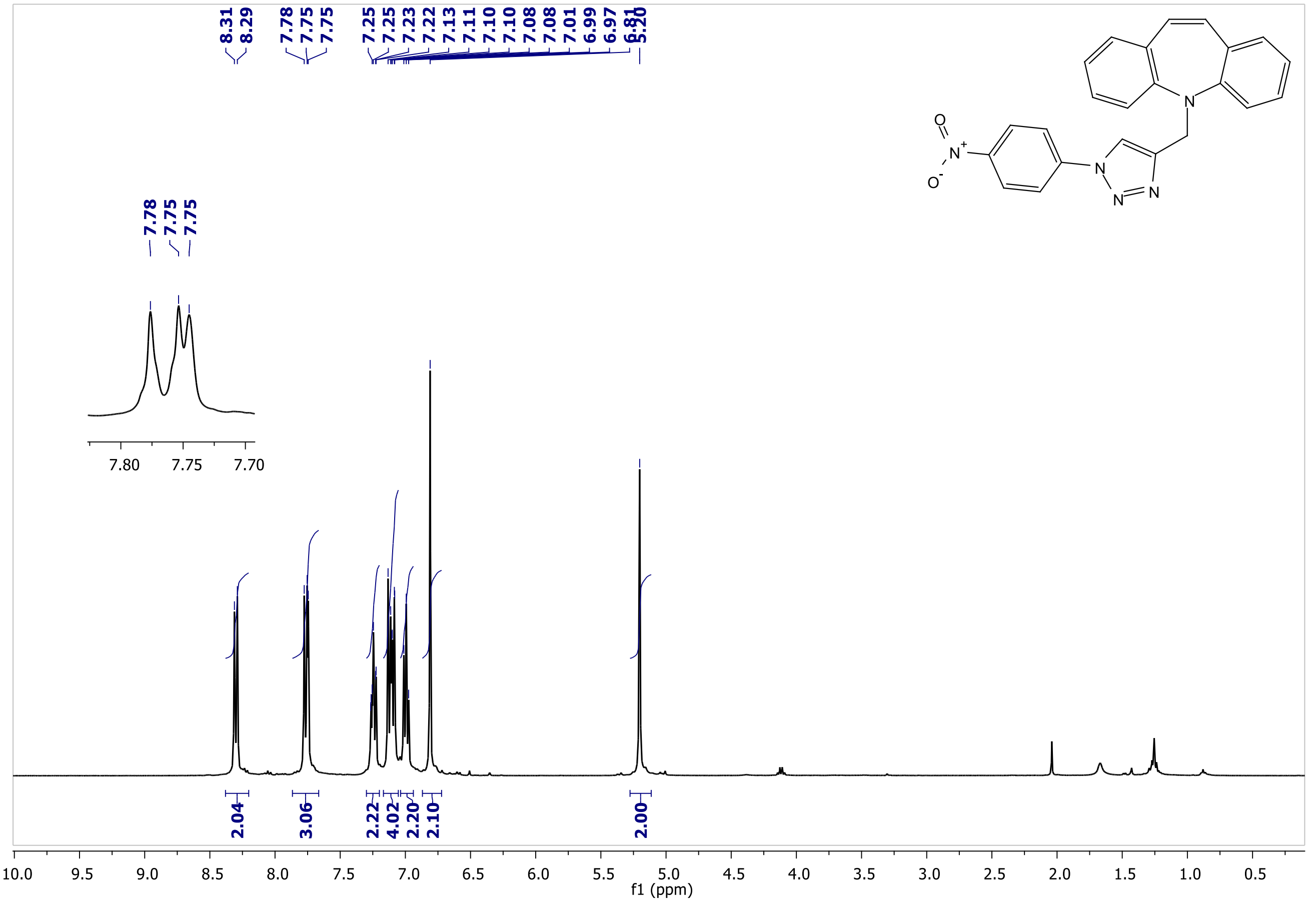

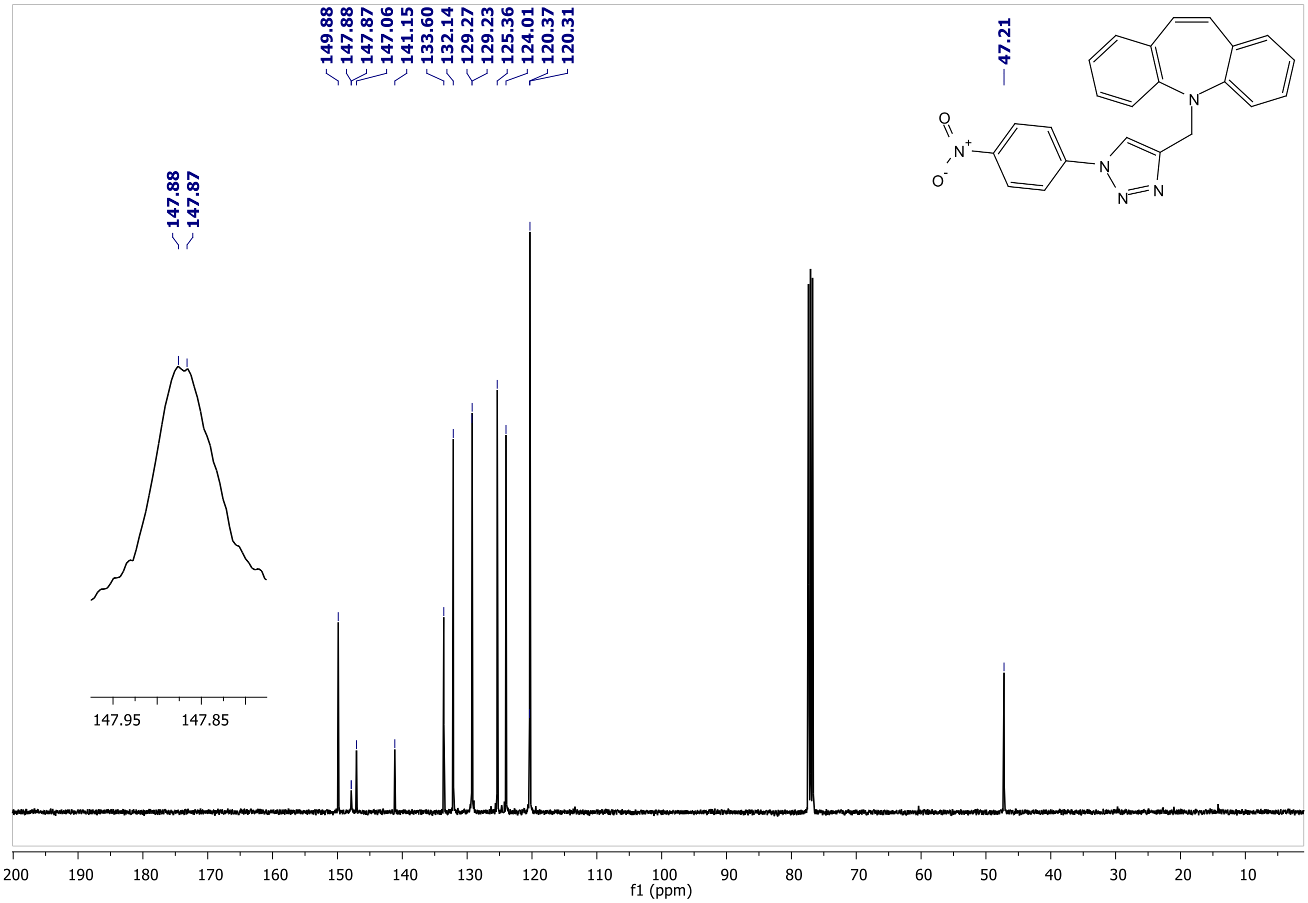

Fig. S8. ^{1}H-NMR (400 MHz, $CDCl_3$) and ^{13}C-NMR (100 MHz, $CDCl_3$) spectrum of **18.**

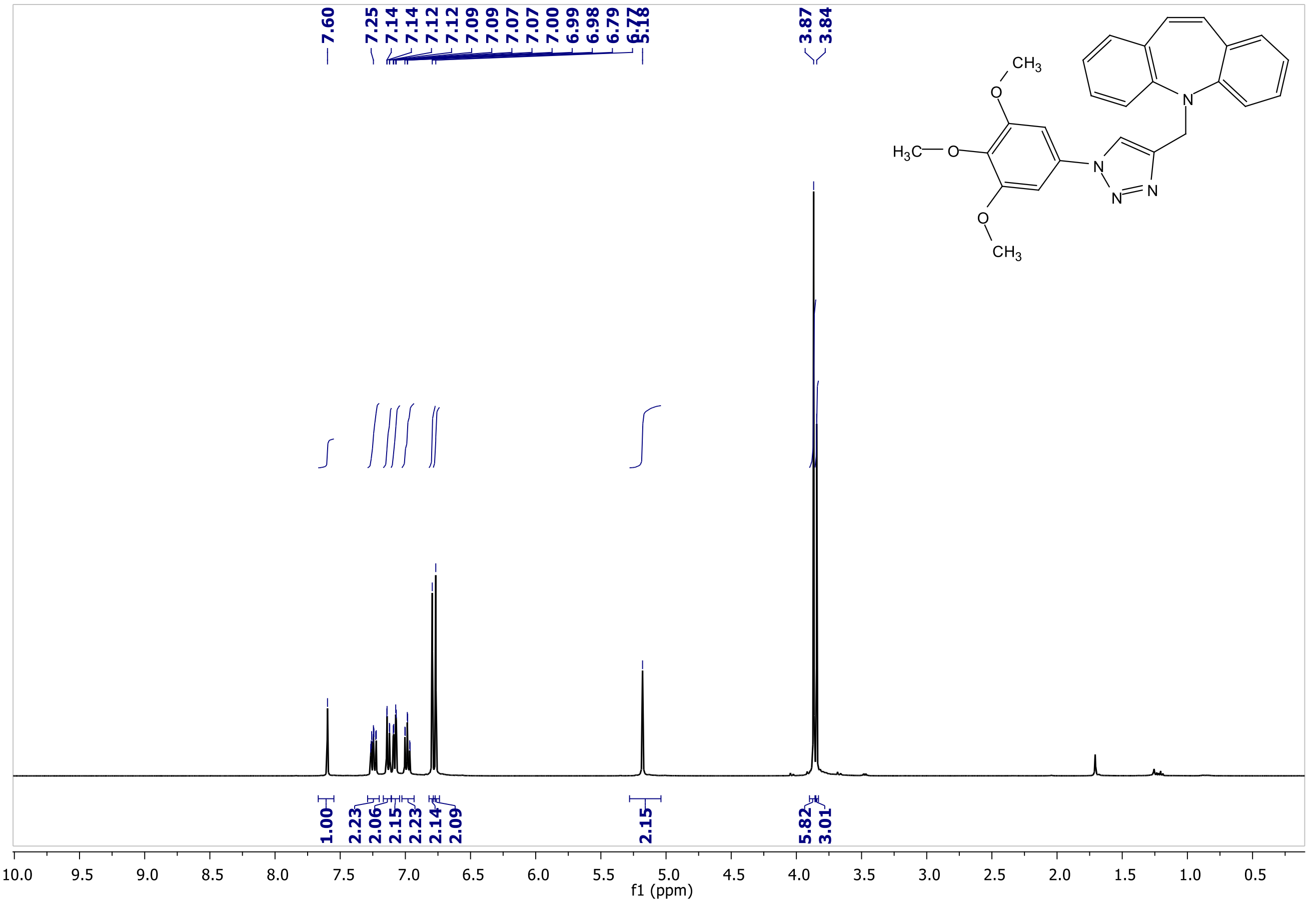

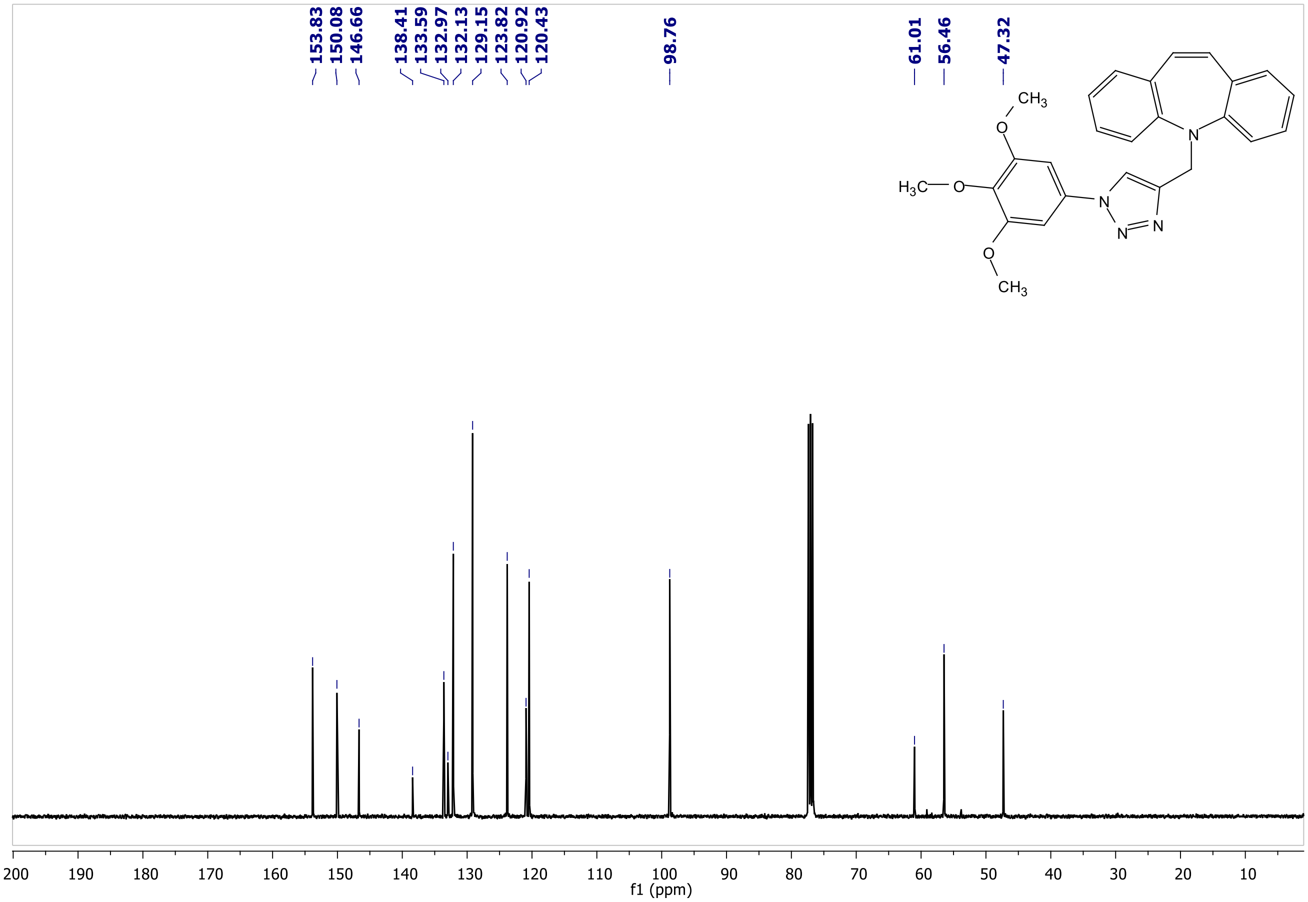

Fig. S9. ^{1}H-NMR (400 MHz, $CDCl_3$) and ^{13}C-NMR (100 MHz, $CDCl_3$) spectrum of **19.**

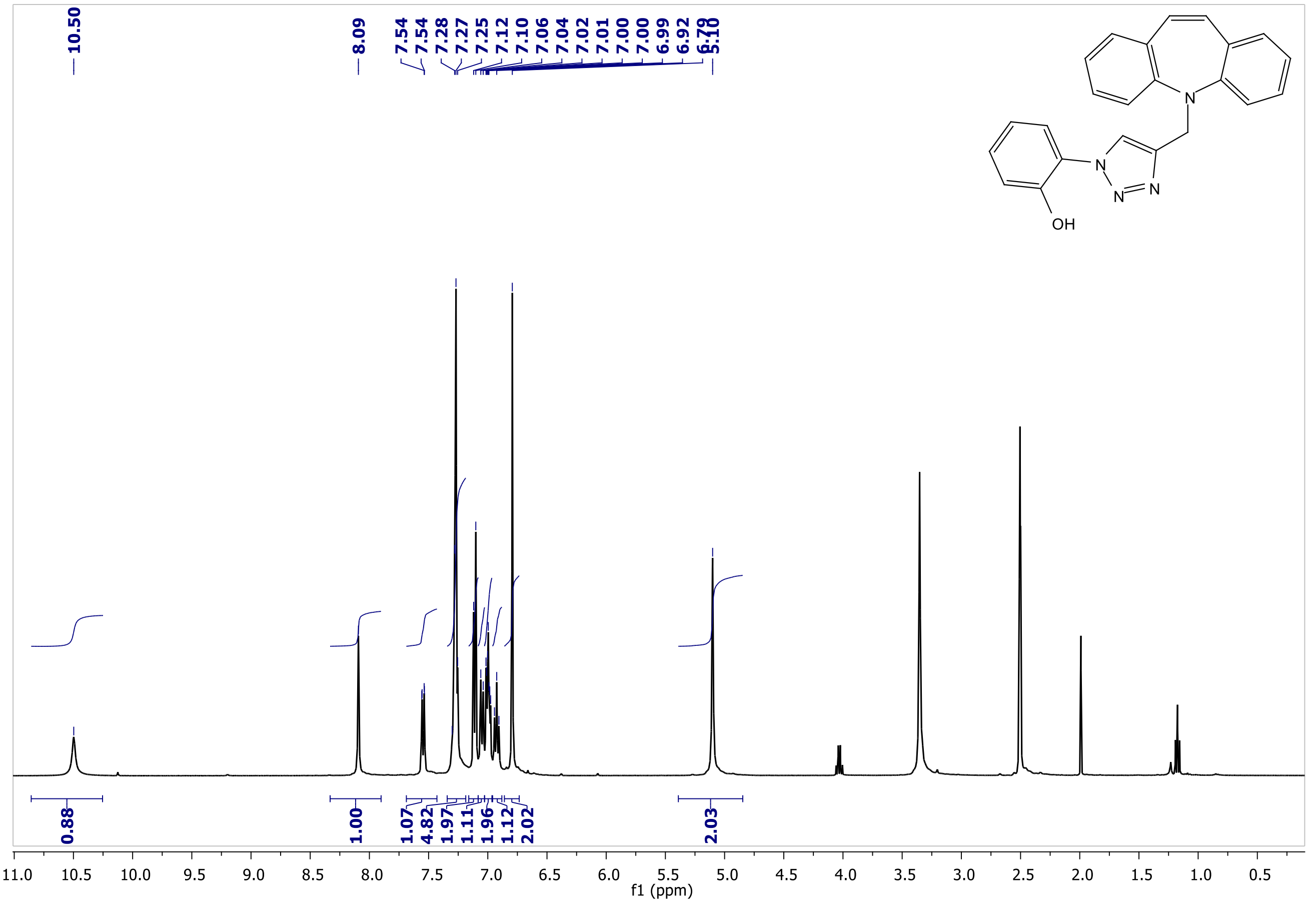

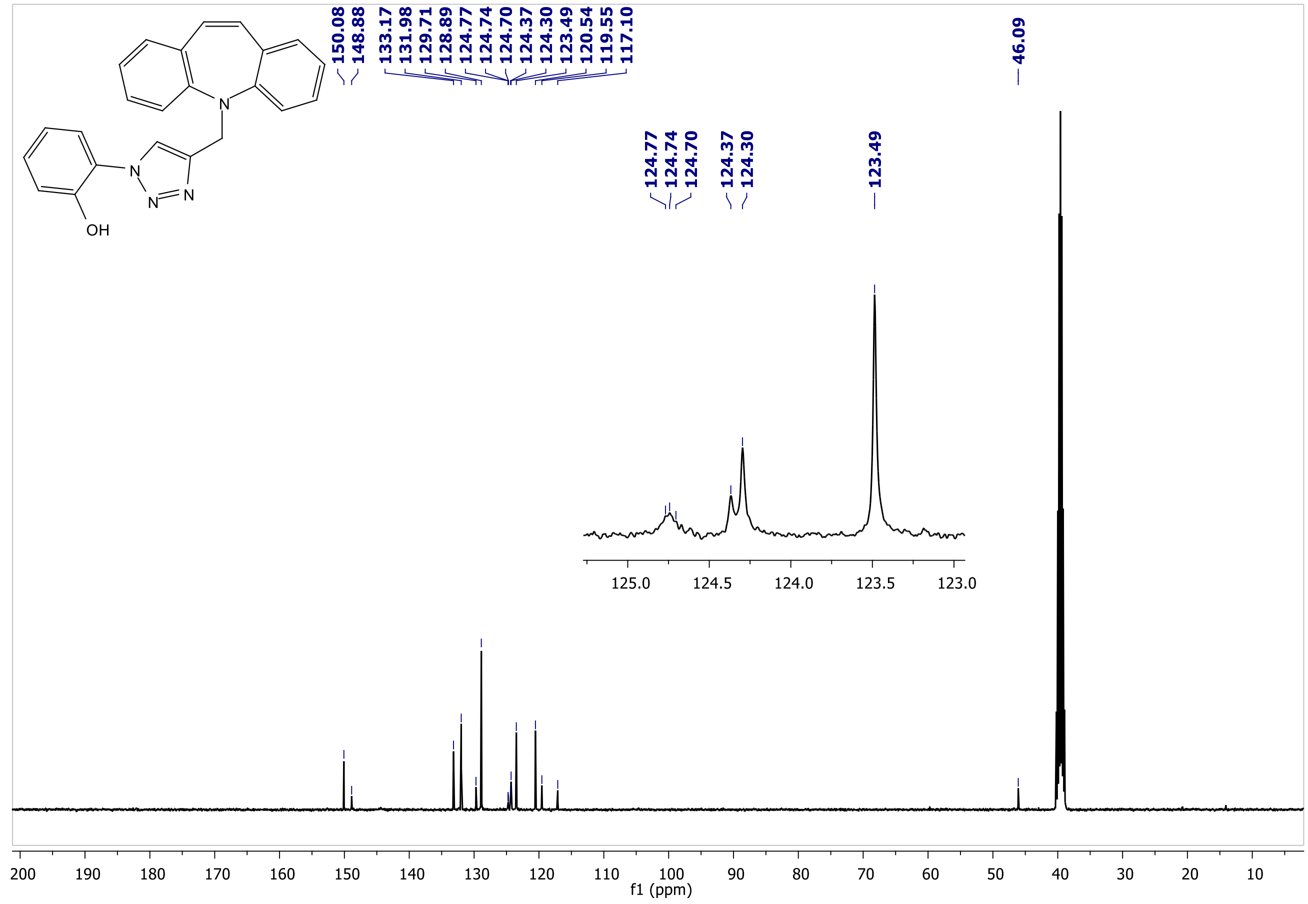

Fig. S10. ^{1}H-NMR (400 MHz, $CDCl_3$) and ^{13}C-NMR (100 MHz, $CDCl_3$) spectrum of **20.**

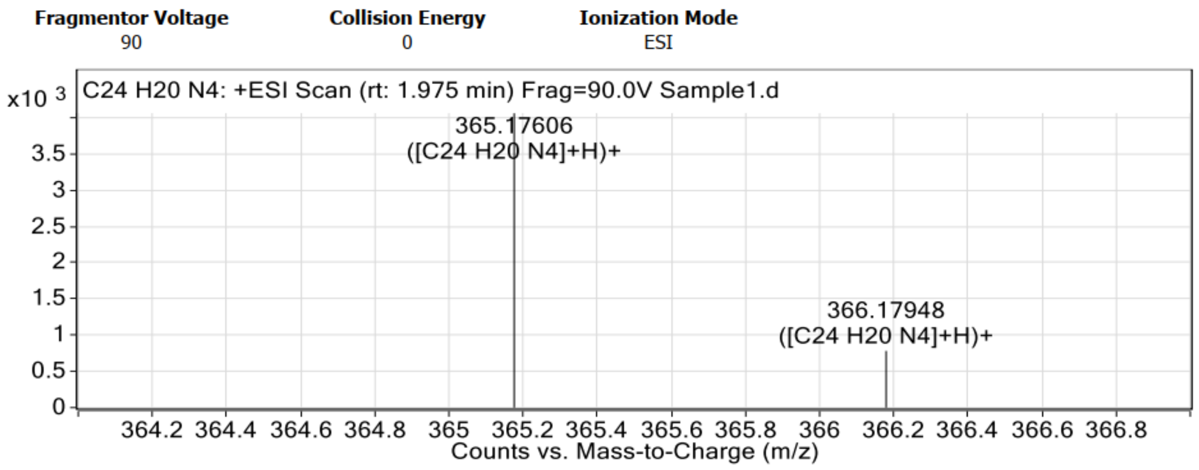

Fig. S11. HRMS spectrum of **12**.

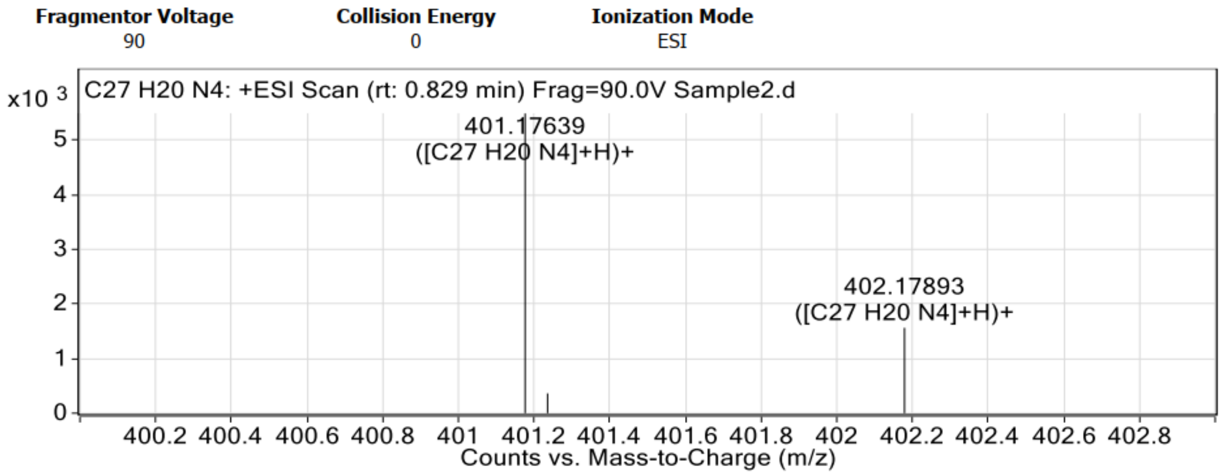

Fig. S12. HRMS spectrum of **13**.

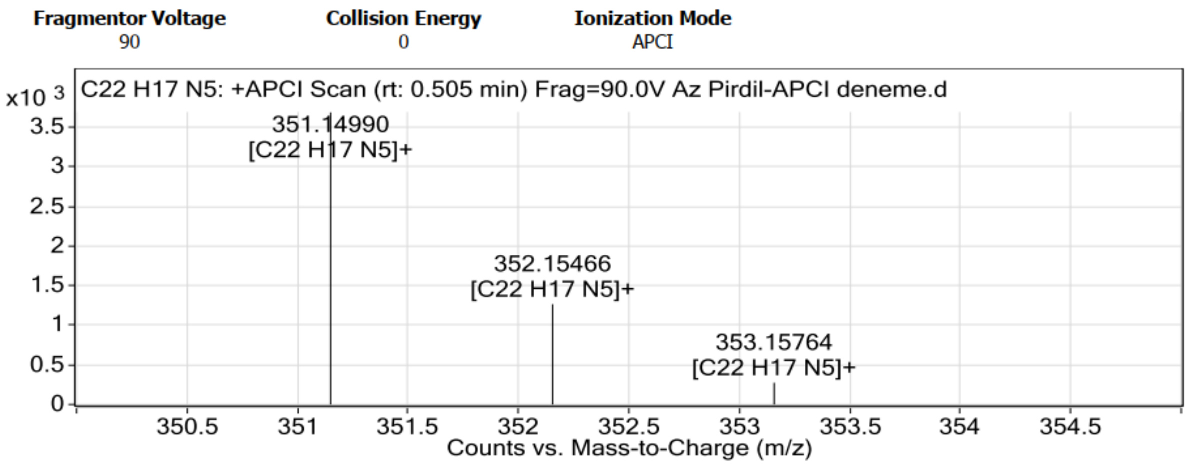

Fig. S13. HRMS spectrum of **14**.

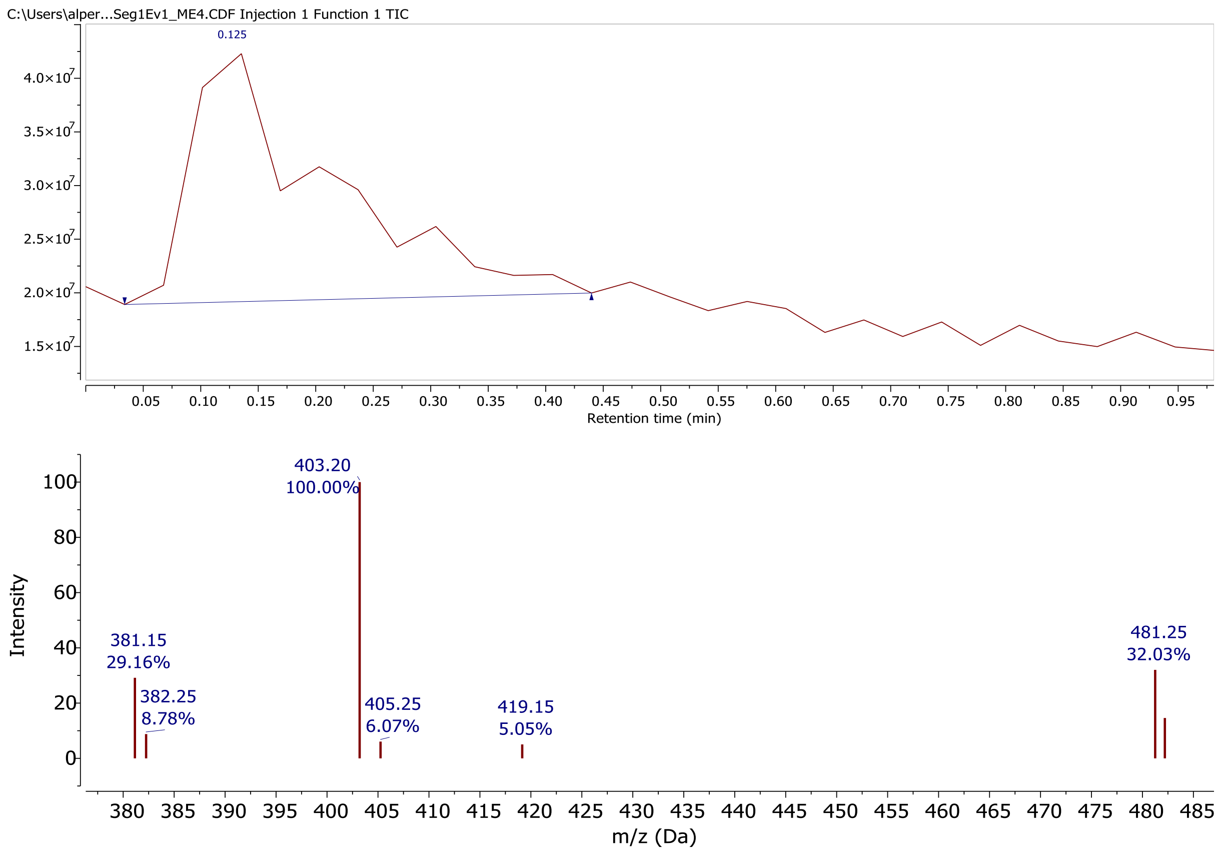

Fig. S14. LC MS/MS spectrum of **15**.

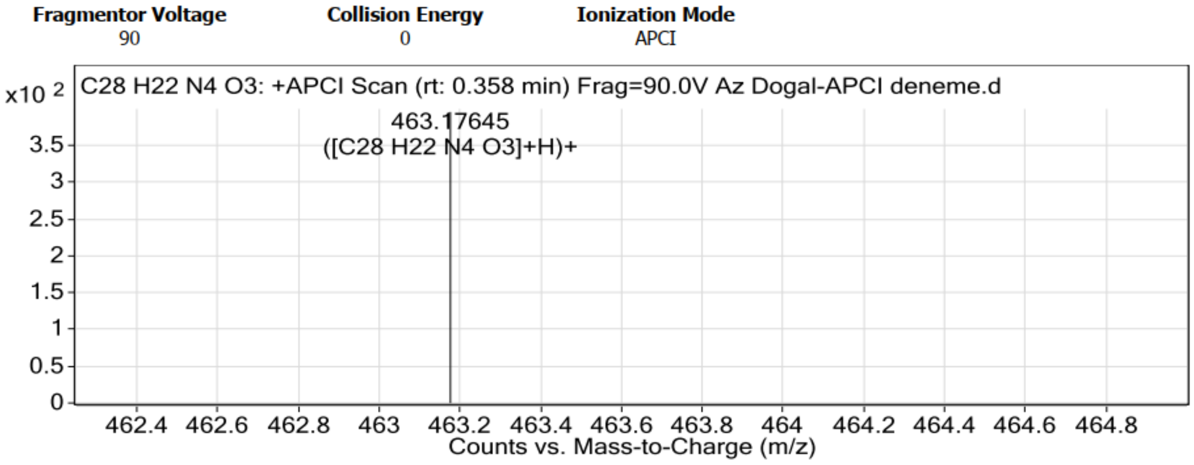

Fig. S15. HRMS spectrum of **16**.

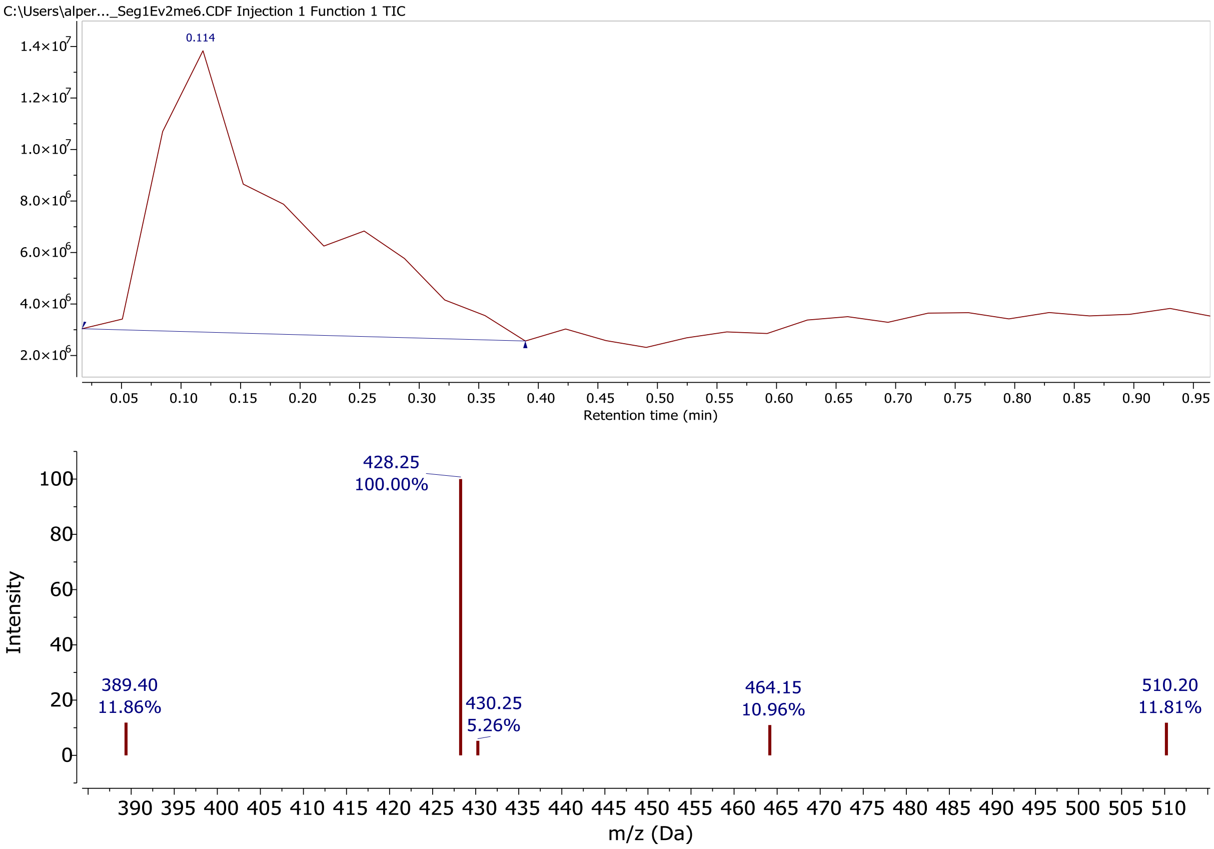

Fig. S16. LC MS/MS spectrum of **17**.

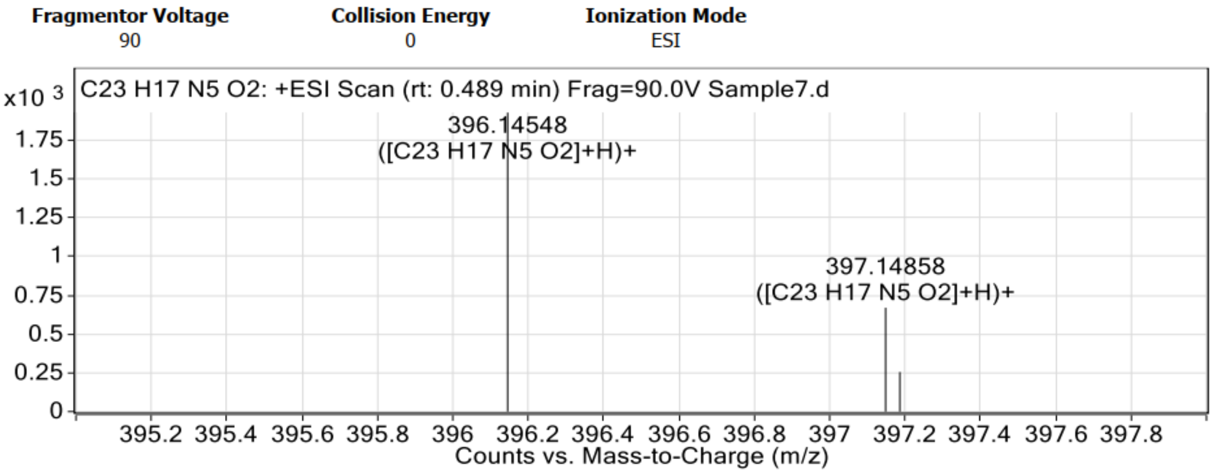

Fig. S17. HRMS spectrum of **18**.

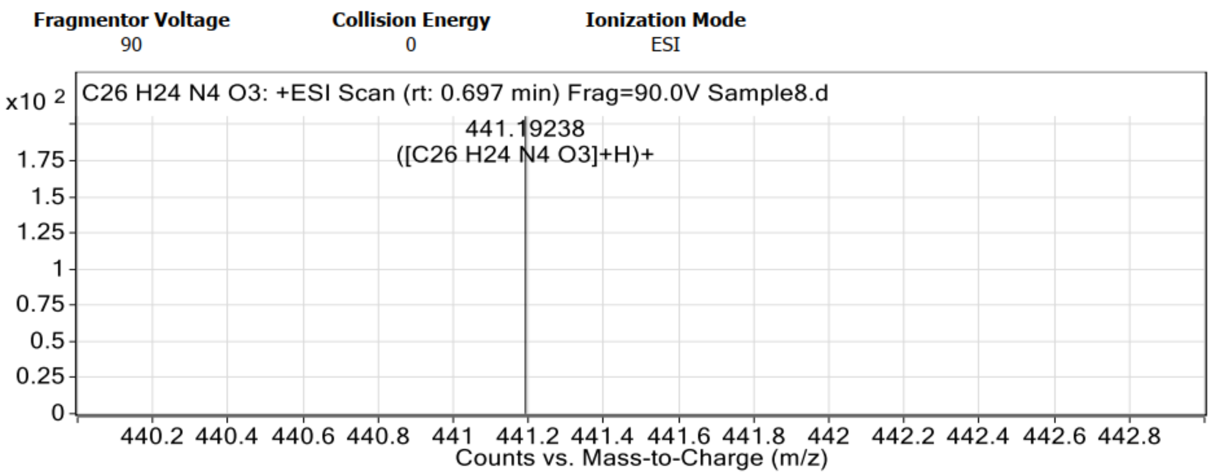

Fig. S18. HRMS spectrum of **19**.

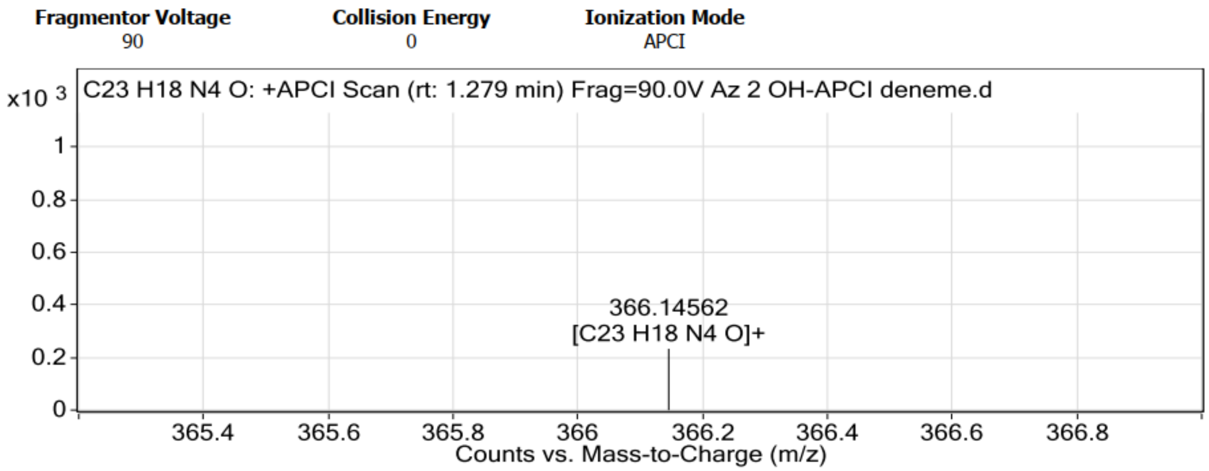

Fig. S19. HRMS spectrum of **20**.

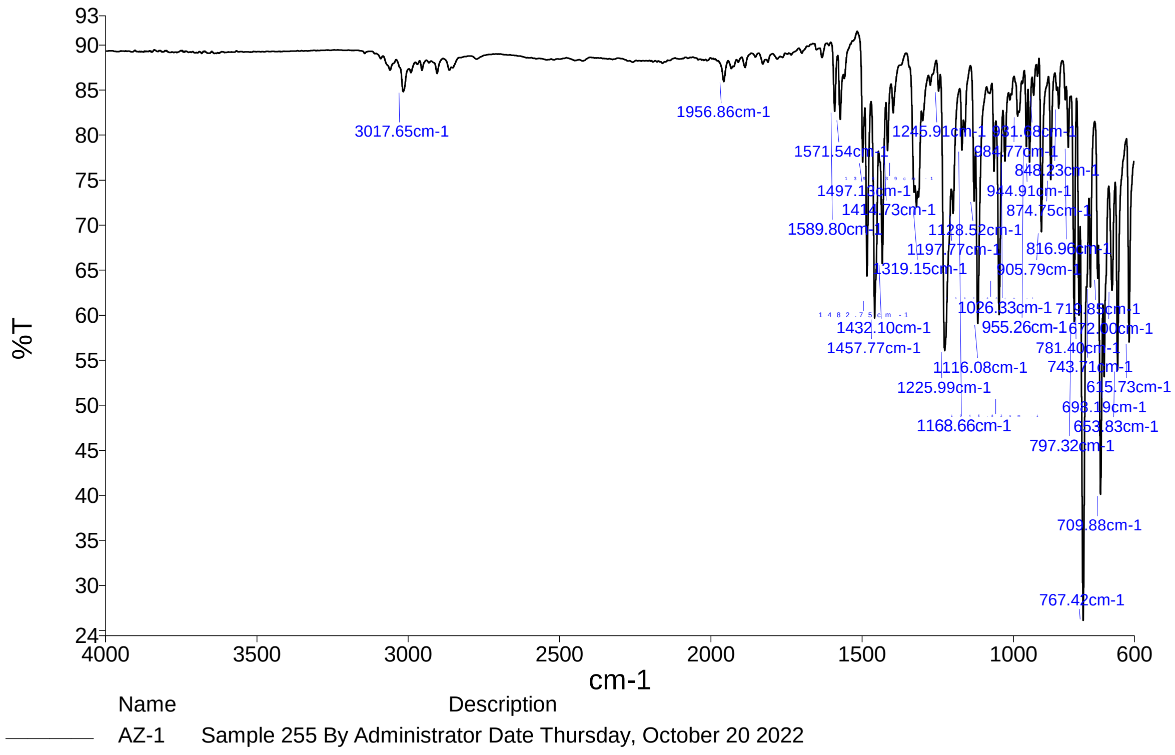

Fig. S20. FTIR spectrum of **12**.

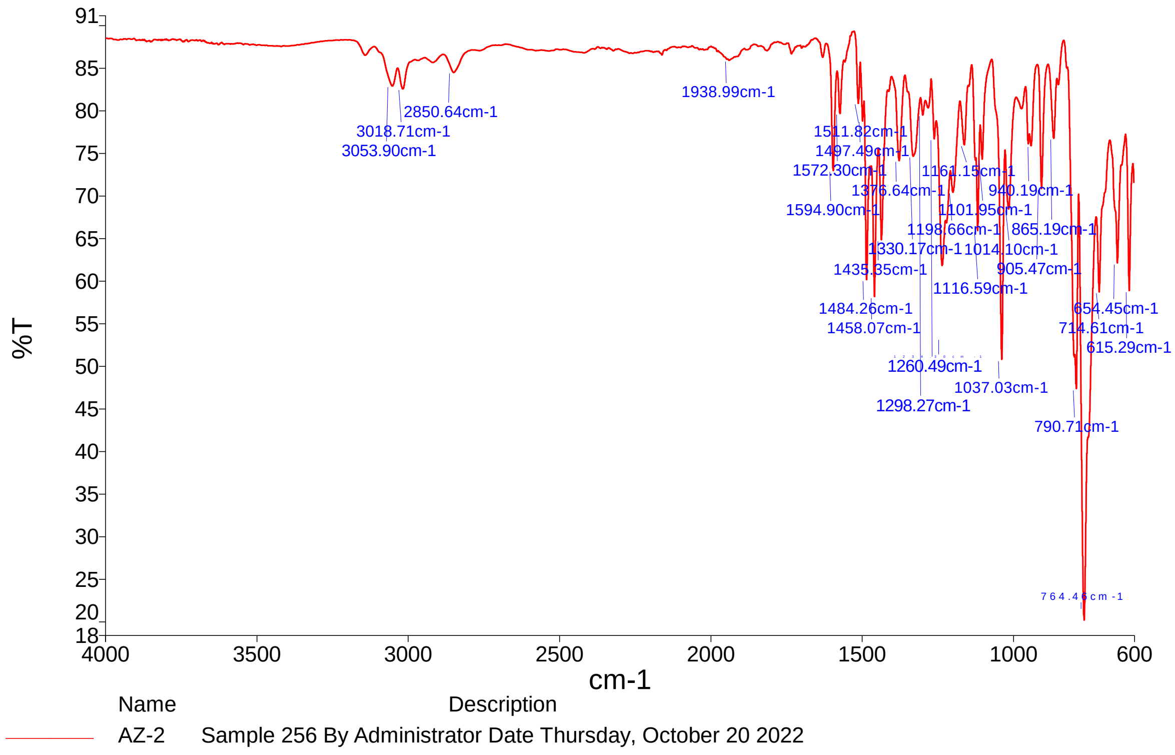

Fig. S21. FTIR spectrum of **13**.

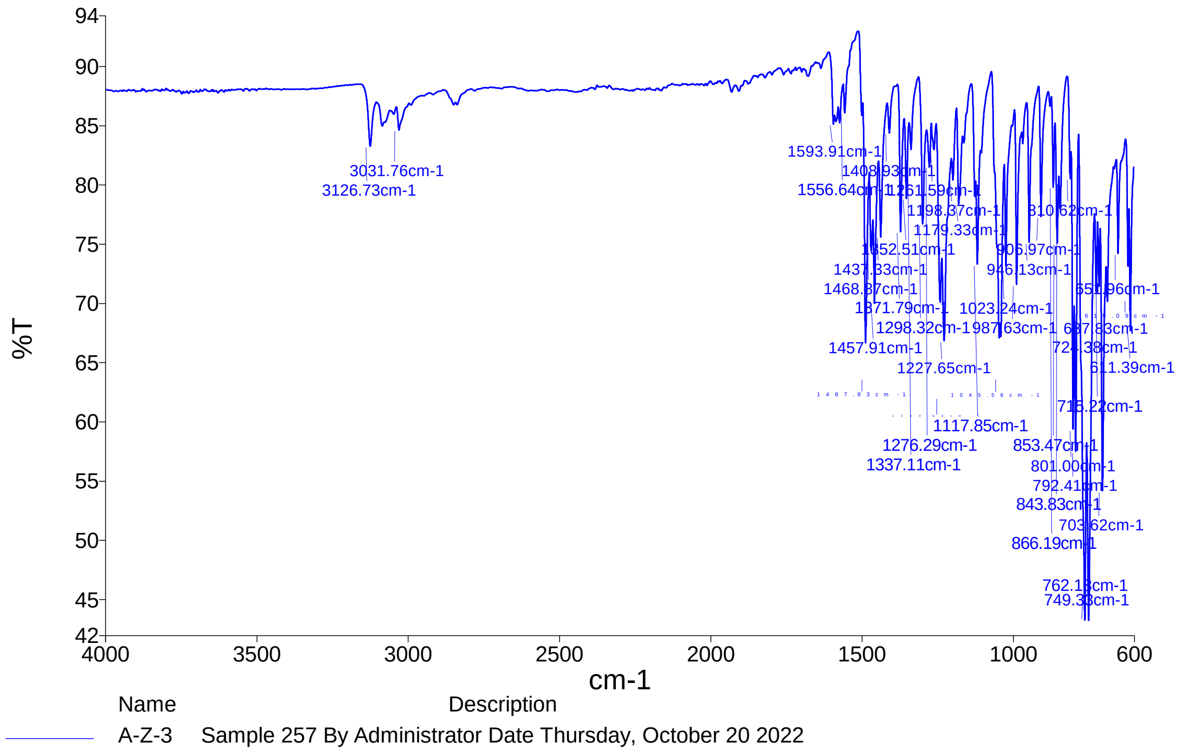

Fig. S22. FTIR spectrum of **14**.

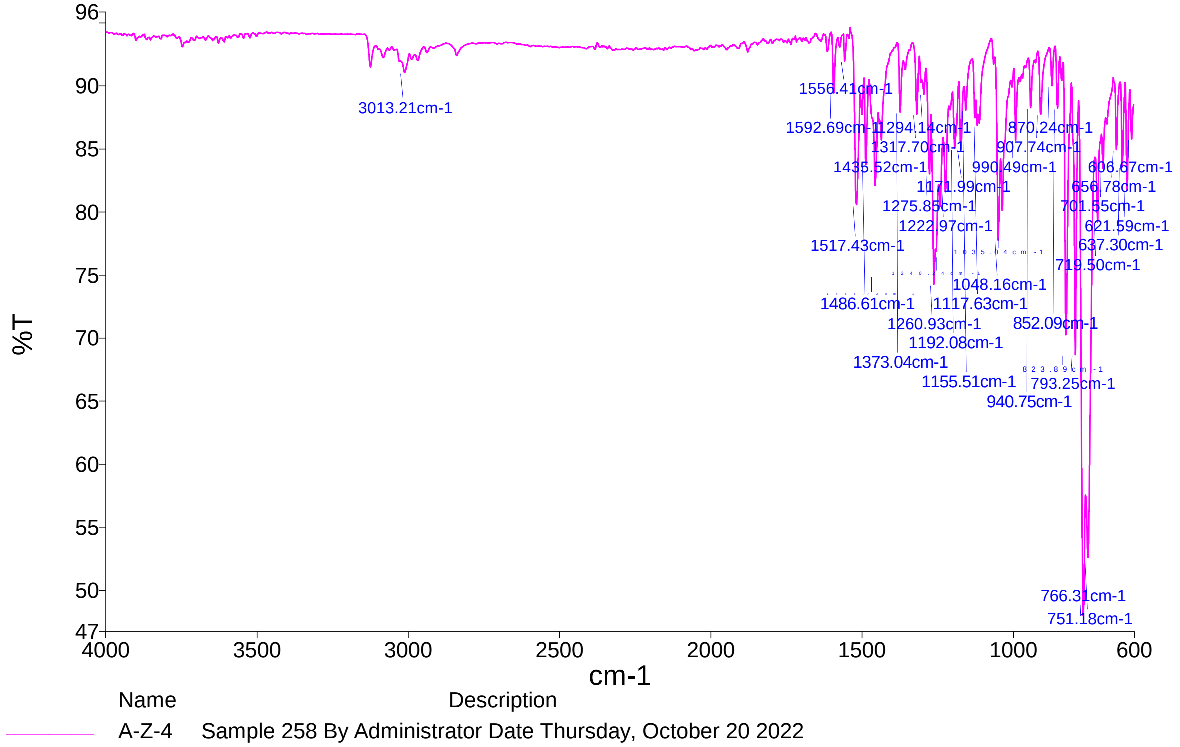

Fig. S23. FTIR spectrum of **15**.

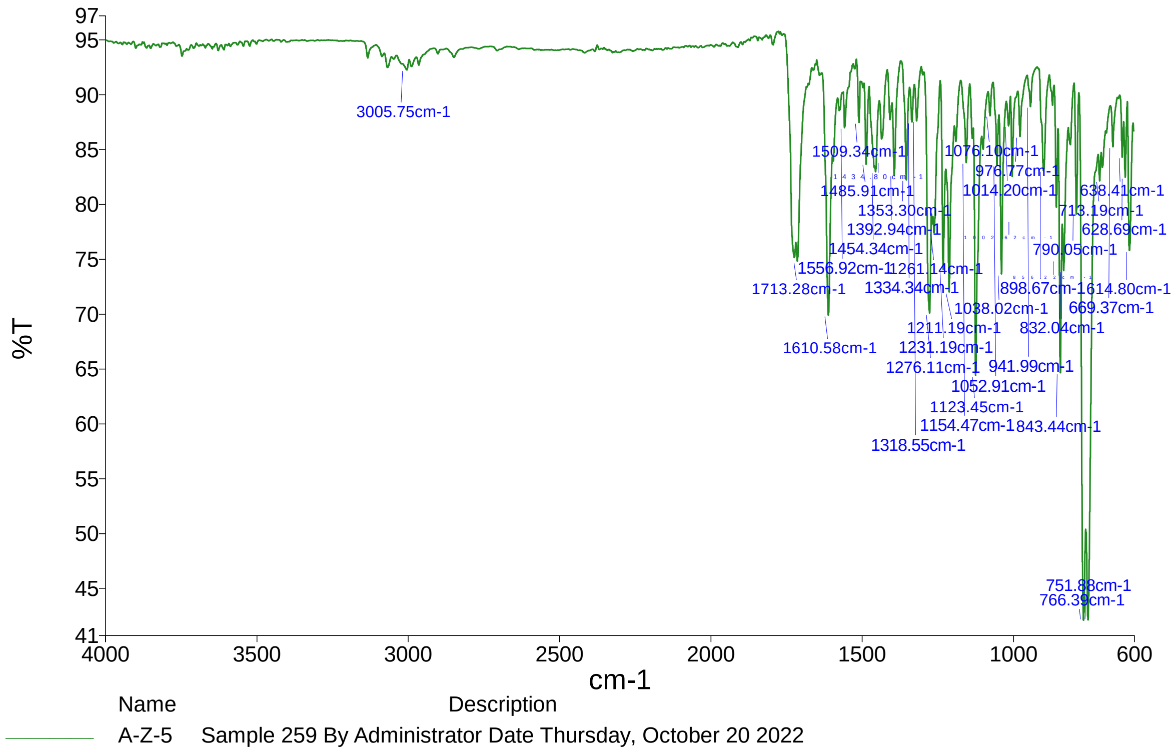

Fig. S24. FTIR spectrum of **16**.

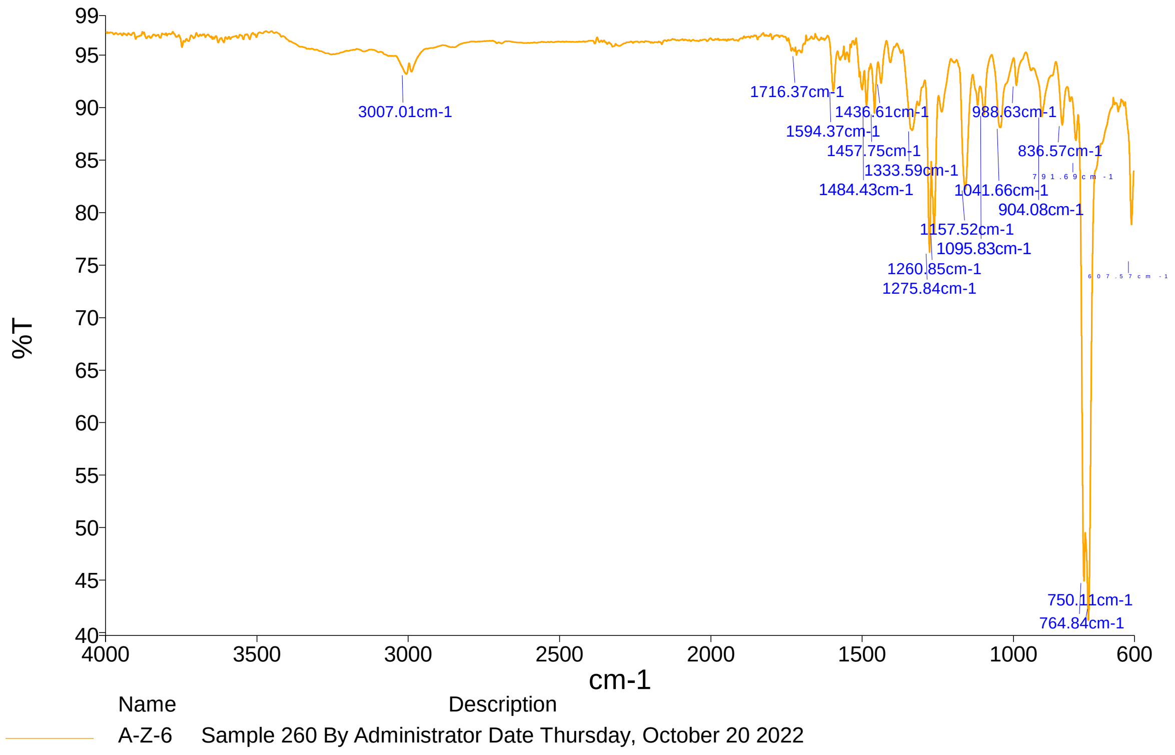

Fig. S25. FTIR spectrum of **17**.

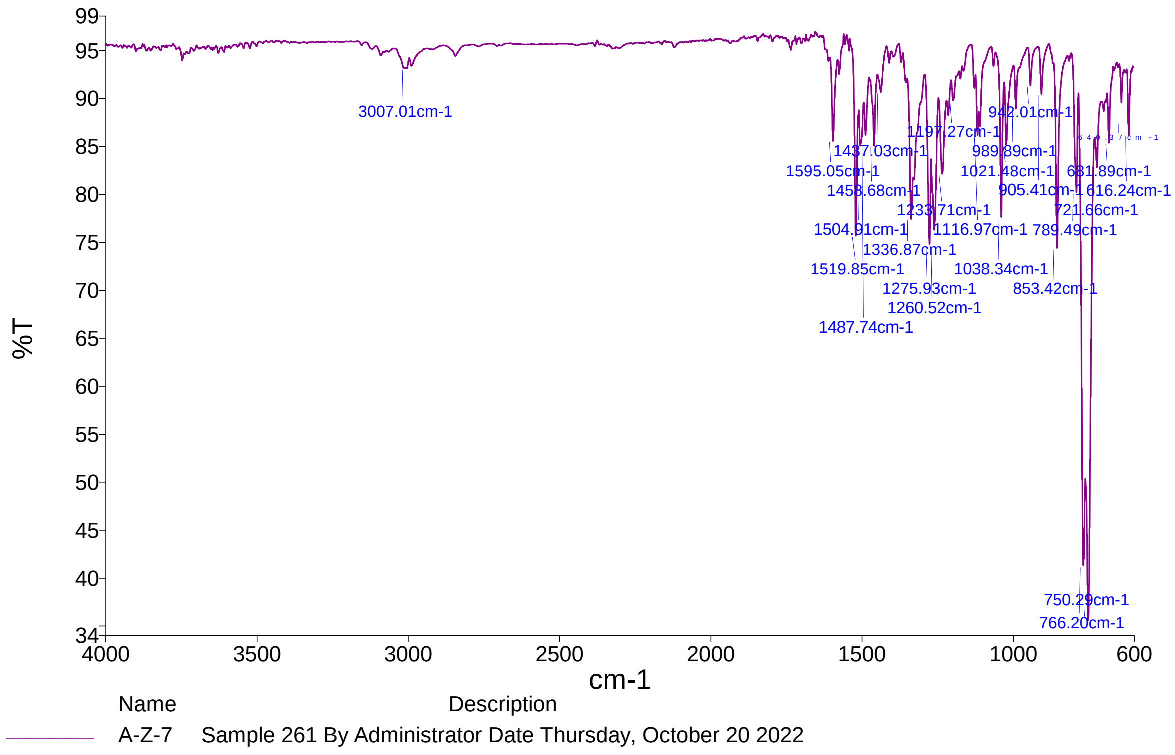

Fig. S26. FTIR spectrum of **18**.

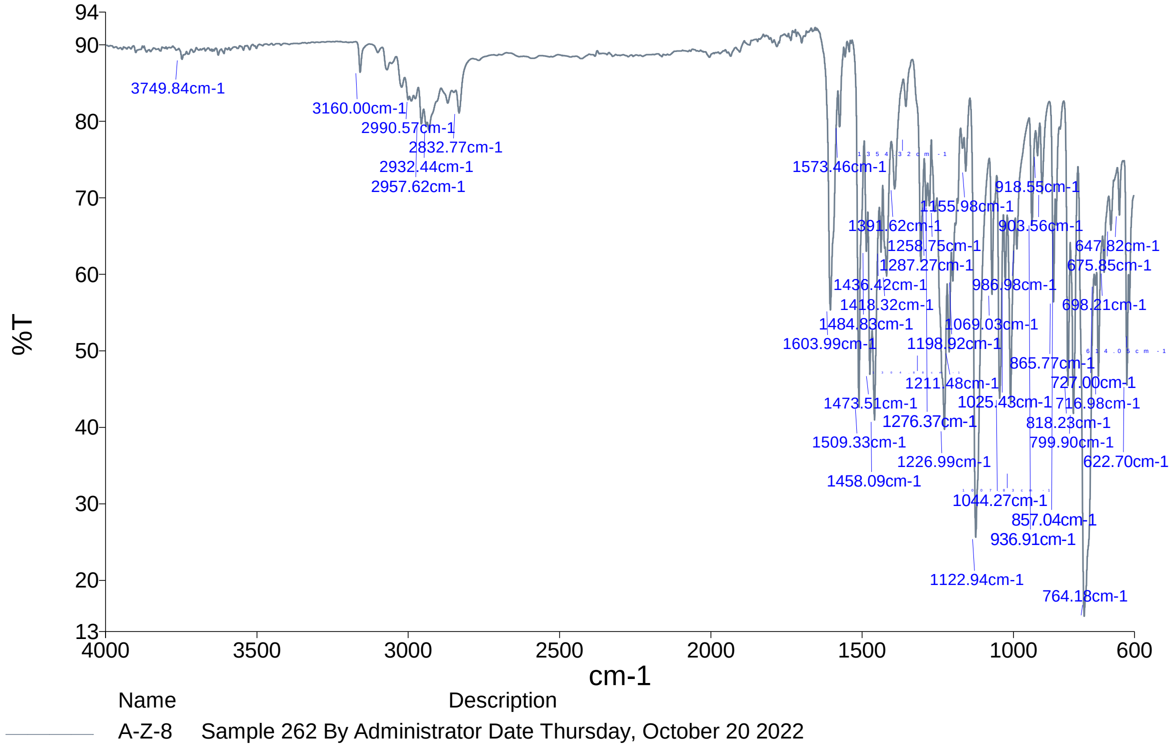

Fig. S27. FTIR spectrum of **19**.

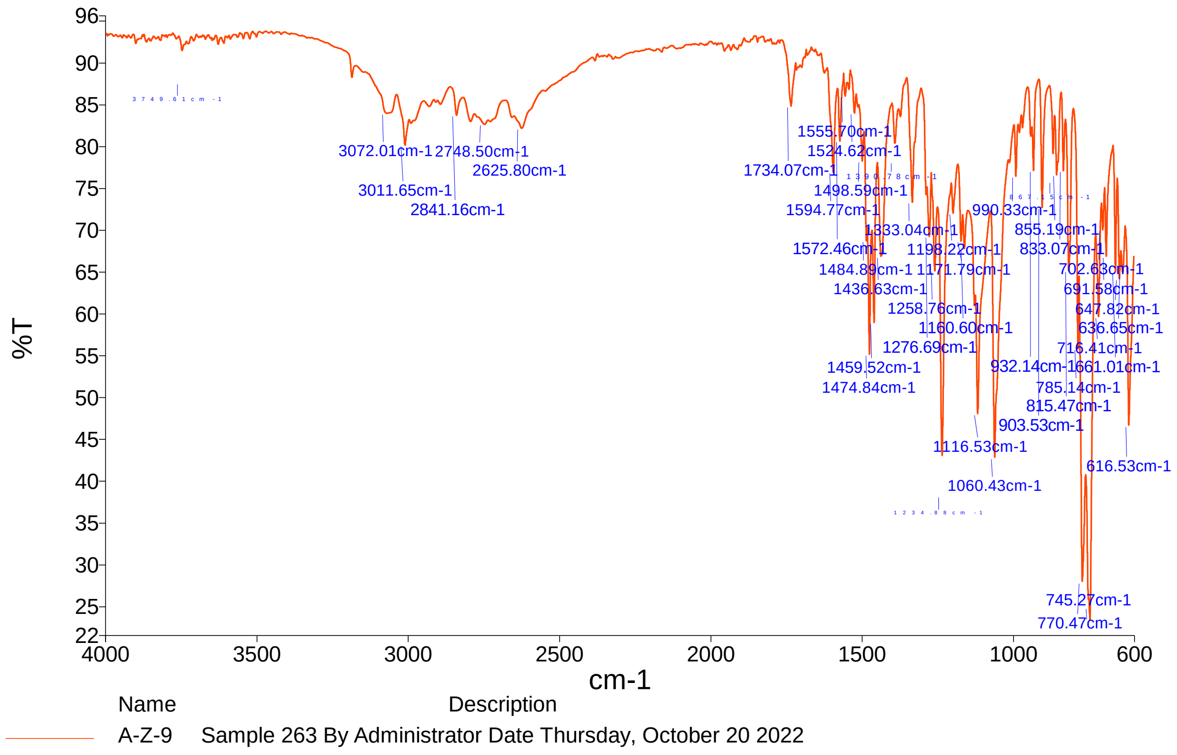

Fig. S28. FTIR spectrum of **20**.

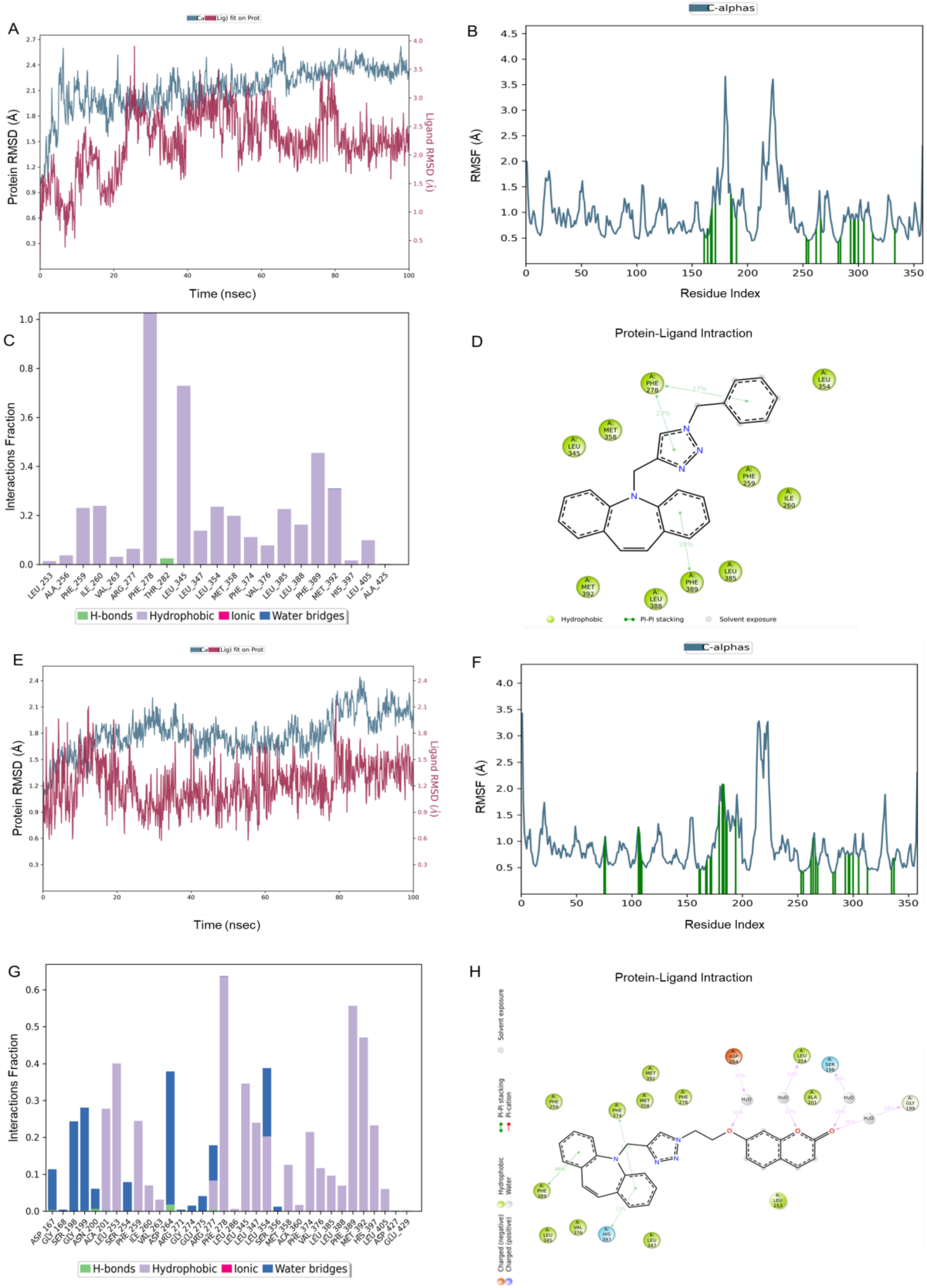

Fig. S29. (A-D) Compound **12** SphK1 (RMSD protein=2.12± 0.27 Å, RMSD lig = 2.22 ± 0.55 Å, ΔG= -78.23 ± 5.37 kcal/mol). (E-H) Compound **16** SphK1 (RMSD protein=1.79± 0.23 Å RMSD ligand = 1.22 ± 0.26 Å, ΔG= -91.81 ± 3.86 kcal/mol).

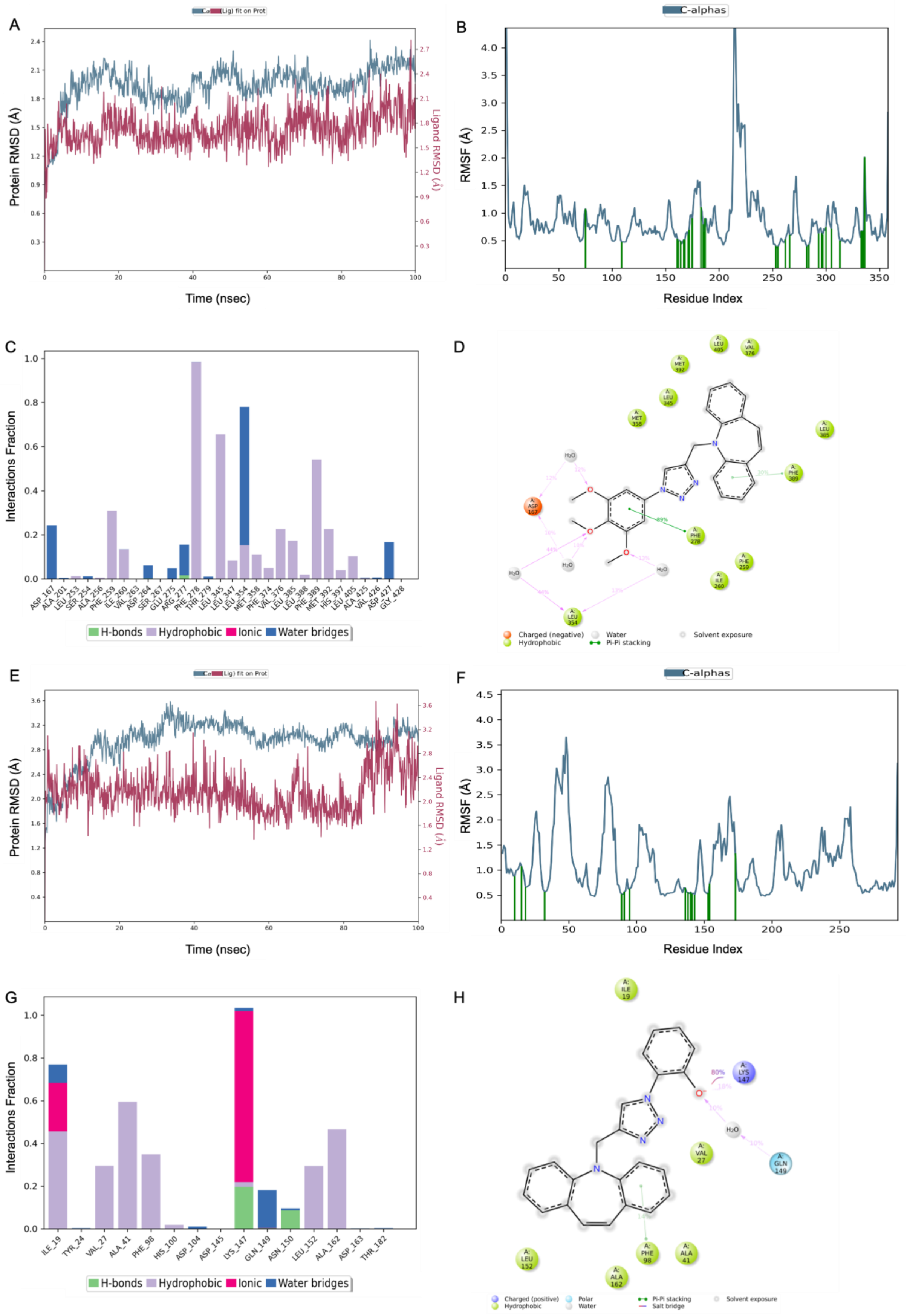

Fig. S30. (A-D) Compound **19** SphK1 (RMSD protein=1.94± 0.22 Å, RMSD ligand = 1.72 ± 0.22 Å, ΔG= -88.10 ± 5.37 kcal/mol). (E-H) Compound **20** CDK6 (RMSD protein=2.94± 0.35 Å RMSD ligand = 2.17 ± 0.35 Å, ΔG= -77.06 ± 2.69 kcal/mol).
